# Transcriptome analysis and differential gene expression profiling of wucai (*Brassica campestris* L.) in response to cold stress

**DOI:** 10.1186/s12864-022-08311-3

**Published:** 2022-02-15

**Authors:** Chenggang Wang, Mengyun Zhang, Jiajie Zhou, Xun Gao, Shidong Zhu, Lingyun Yuan, Xilin Hou, Tongkun Liu, Guohu Chen, Xiaoyan Tang, Guolei Shan, Jinfeng Hou

**Affiliations:** 1 College of Horticulture, Vegetable Genetics and Breeding Laboratory, Anhui Agricultural University, 130 West Changjiang Road, Hefei, Anhui, 230036 China; 2 Provincial Engineering Laboratory for Horticultural Crop Breeding of Anhui, 130 West of Changjiang Road, Hefei, Anhui, 230036 China; 3 Wanjiang Vegetable Industrial Technology Institute, Maanshan, 238200 Anhui China; 4 Department of Horticulture, Nanjing Agricultural University, Nanjing, Jiangsu, 210095 China

**Keywords:** *Brassica campestris*, RNA-seq, Cold stress, α-linolenic acid metabolism

## Abstract

**Background:**

Wucai suffers from low temperature during the growth period, resulting in a decline in yield and poor quality. But the molecular mechanisms of cold tolerance in wucai are still unclear.

**Results:**

According to the phenotypes and physiological indexes, we screened out the cold-tolerant genotype “W18” (named CT) and cold-sensitive genotype “Sw-1” (named CS) in six wucai genotypes. We performed transcriptomic analysis using seedling leaves after 24 h of cold treatment. A total of 3536 and 3887 differentially expressed genes (DEGs) were identified between the low temperature (LT) and control (NT) comparative transcriptome in CT and CS, respectively, with 1690 DEGs specific to CT. The gene ontology (GO) analysis showed that the response to cadmium ion (GO:0,046,686), response to jasmonic acid (GO:0,009,753), and response to wounding (GO:0,009,611) were enriched in CT (LT vs NT). The DEGs were enriched in starch and sucrose metabolism and glutathione metabolism in both groups, and α-linolenic acid metabolism was enriched only in CT (LT vs NT). DEGs in these processes, including *glutathione S-transferase*s (*GST*s), 13S lipoxygenase (*LOX*), and *jasmonate ZIM-domain* (*JAZ*), as well as transcription factors (TFs), such as the ethylene-responsive transcription factor 53 (ERF53), basic helix-loop-helix 92 (bHLH92), WRKY53, and WRKY54.We hypothesize that these genes play important roles in the response to cold stress in this species.

**Conclusions:**

Our data for wucai is consistent with previous studies that suggest starch and sucrose metabolism increased the content of osmotic substances, and the glutathione metabolism pathway enhance the active oxygen scavenging. These two pathways may participated in response to cold stress. In addition, the activation of α-linolenic acid metabolism may promote the synthesis of methyl jasmonate (MeJA), which might also play a role in the cold tolerance of wucai.

**Supplementary Information:**

The online version contains supplementary material available at 10.1186/s12864-022-08311-3.

## Background

Low temperature is one of the abiotic stresses that directly affect plant growth and development [1]. It also affects the geographical distribution of plants and limits their growth [2]. In response to adverse environmental conditions, plants have evolved a series of physiological and biochemical mechanisms. In plants, there are many receptors for different stress signals, which are involved in various stress responses and form a complex response and regulation network in response to stress [3]. When plants are subjected to low temperature stress, a large number of soluble substances accumulate in their tissues to improve cold resistance. For example, starch will be hydrolyzed into simple sugars and other derived metabolites to increase the osmotic pressure of cells to prevent freezing [4]. Plants accumulate a large amount of reactive oxygen species (ROS) under stress. ROS mainly include superoxide anions (O_2_·^−^), hydrogen peroxide (H_2_O_2_), hydroxyl radicals (·OH), and singlet oxygen (O_2_^1^) [5]. In order to maintain the intracellular ROS balance at a harmless concentration, plants have evolved a series of enzymatic and non-enzymatic ROS-scavenging mechanisms [6]. Under stress, plants can regulate the activity of these enzymes or antioxidants through a series of physiological and biochemical mechanisms [7]. In this way, the excessive accumulation of ROS in plants can be reduced and the stress resistance of plants can be improved.

Plant hormones such as cytokinin, abscisic acid, ethylene and jasmonic acid (JA) also play an important direct or indirect role in plant response to abiotic stress. Jasmonic acid and its derivatives, such as MeJA and cis-jasmone, are collectively referred to as jasmonates (JAs). They are fatty acids derived from cyclopentanone. They belong to the oxidized lipid family and are collectively referred to as oxidized lipids [8]. In addition to responding to biological stress, they can also regulate the expression of many genes in response to abiotic stress, such as low temperature and salinity [9]. Studies have shown that MeJA can activate antioxidant metabolic pathways and defense mechanisms in various crops and enhance cold tolerance [10]. In addition, some members of ERF, bHLH, MYB, DREB, and WRKY transcription factor families, such as ERF53 [11], ICE1, DREB1A [12], MYB4 [13], and WRKY19 [14], are also involved in regulating the expression of cold-stress response genes.

High-throughput RNA-sequencing (RNA-seq) can precisely measure the transcription level and provide gene sequence information at the same time. It has high efficiency, high cost performance with high reliability, and is widely used in plant transcriptome analysis, especially in non-model plants that lack genome sequencing data [15]. Over the last decade, RNA-seq has been used in many plant species to investigate plant responses to cold stress, including winter rapeseed [16], strawberry [17], and cotton [18], and has been confirmed as a powerful tool for plant genetics research [19].

Wucai (*Brassica campestris* L. ssp. *chinensis* var. *rosularis* Tsen) is a genotype of Chinese cabbage and is native to China [20]. It is one of the main vegetables cultivated in the Yangtze–Huaihe River basin [21]. Wucai is a semi-cold-tolerant vegetable, and the optimum growth temperature is 10 °C-20°C. It is a popular vegetable in autumn and winter for its nutritional value and good taste. However, different genotypes of the same species have different tolerance levels to cold stress [22]. With the expansion of the planting area, it is an important task to select cold-tolerant genotypes of wucai.

The response of different genotypes to cold varies from tolerant to extremely sensitive. On the basis of previous studies, we screened six genotypes of wucai for their cold tolerance at the seedling stage [23]. Through a study of the morphological, physiological, and biochemical properties of wucai under both control and cold stress conditions, cold-tolerant and sensitive genotypes were selected. We further conducted complete transcriptome sequencing on the cold-tolerant and sensitive wucai genotypes, providing valuable insights into the molecular mechanisms of this variety. The results lay a foundation for identifying genes with the potential to improve the cold tolerance of wucai, as well for developing cold-tolerant wucai varieties in future breeding programs.

## Results

### Phenotypic and physiological differences between various genotypes under cold stress

**Fig. 1:**
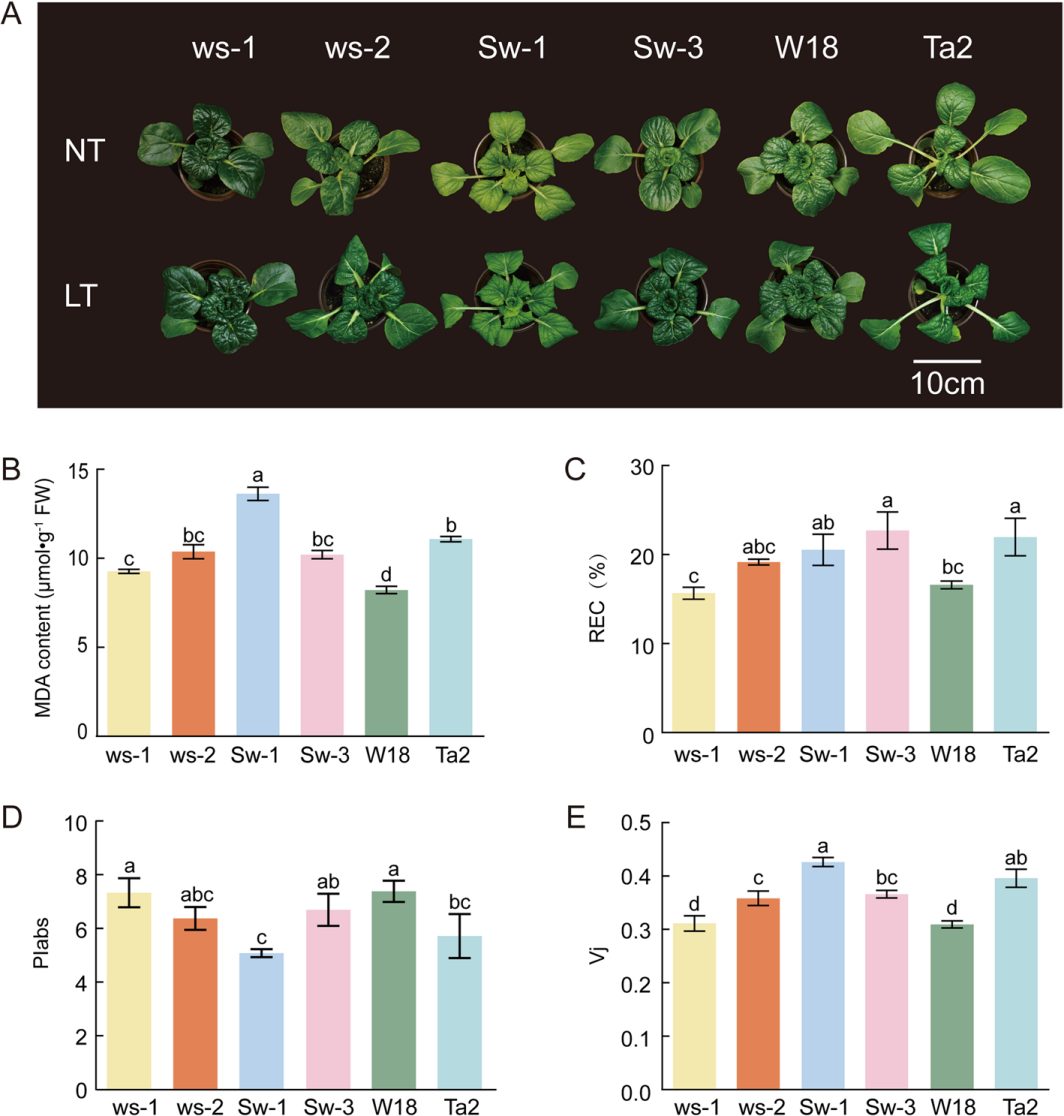
Measurement of plant physiological parameters in wucai seedlings at the 7-leaf stage subjected to 3-day cold stress. **A**: Phenotypic change under cold stress in six wucai genotypes, ws-1, ws-2, Sw-1, Sw-3, W18 and Ta2. **B**: MDA content under cold stress in six wucai genotypes. **C**: Relative electrical conductivity under cold stress in six wucai genotypes. **D**: PI_abs_ under cold stress in six wucai genotypes. **E**: V_j_ under cold stress in six wucai genotypes. The data are presented as the mean ± SD of three replicates. Bars with different letters are significantly different at *P* < 0.05 (ANOVA followed by Tukey’s test)

The morphological characteristics of seedlings of six wucai genotypes were determined to select cold-tolerant and cold-sensitive genotypes (Fig. 1A). Based on measurements of the MDA content in leaves under stress and normal conditions, changes in the leaf lipid hydrogen peroxide production rate were studied. After cold treatment, the MDA content in Sw-1 reached the highest level, whereas that in W18 was at the lowest level (Fig. 1B). Relative electrolyte conductivity (REC) in Sw-3 was the highest, followed by Ta2, Sw-1, ws-2, W18 and ws-1 (Fig. 1C).

The performance index on an absorption basis (PI_abs_) of W18 was the highest and was significantly higher than that of Sw-1 (Fig. 1D). V_j_ increased the most in Sw-1, followed by Ta2, Sw-3, ws-2, ws-1 and W18 (Fig. 1E). These indexes indicated that W18 is a cold-tolerant wucai genotype and Sw-1 is a cold-sensitive genotype.

### Differential responses of two wucai genotypes to cold stress

**Fig. 2:**
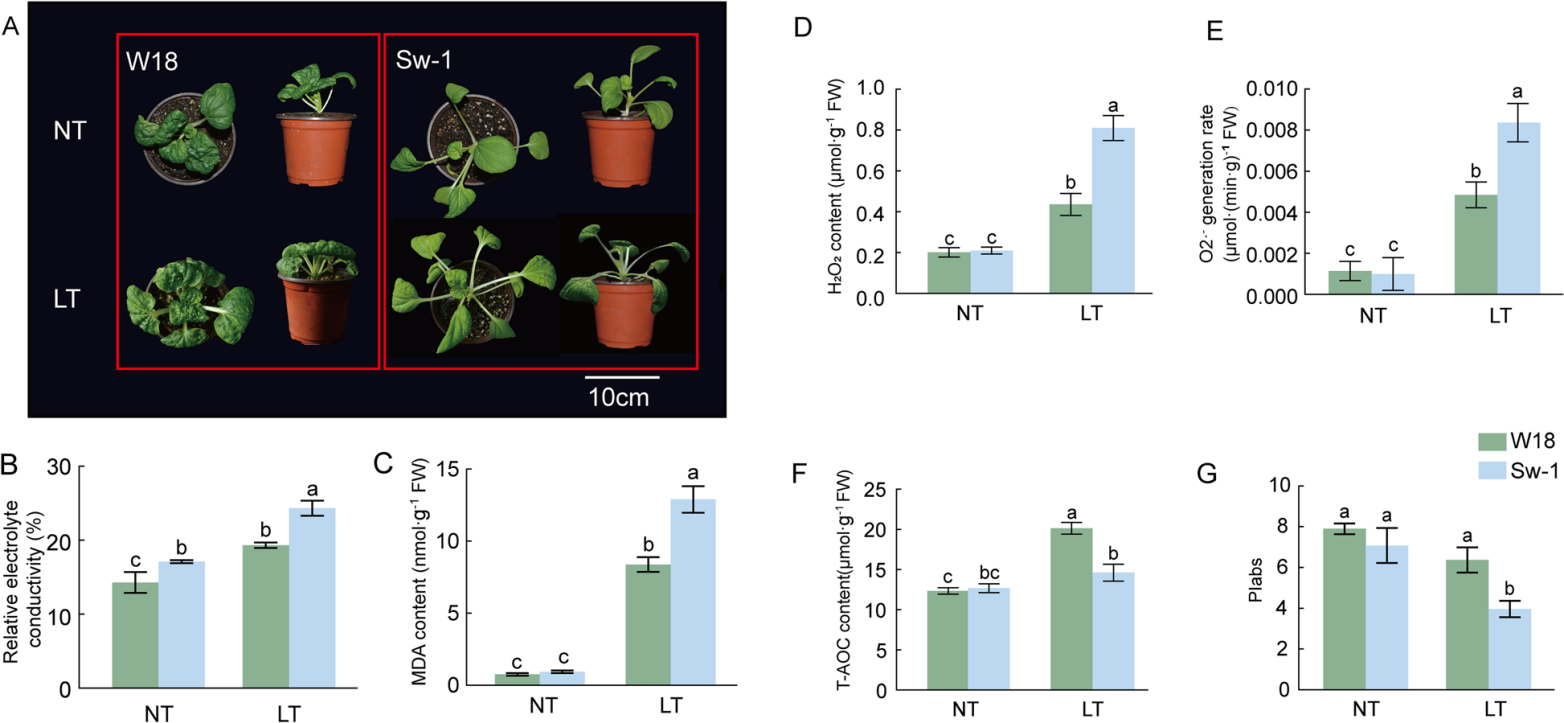
Measurement of plant physiological parameters in wucai seedlings at the 7-leaf stage subjected to 24-h cold stress. **A**: Phenotypic change in W18 and Sw-1 under cold stress. **B**: Relative electrical conductivity in W18 and Sw-1 under control and cold stress conditions. **C**: MDA content in W18 and Sw-1 under control and cold stress conditions. **D**: H_2_O_2_ content in W18 and Sw-1 under control and cold stress conditions. **E**: O_2_·^−^ generation rate in W18 and Sw-1 under control and cold stress conditions. **F**: T-AOC content in W18 and Sw-1 under control and cold stress conditions. **G**: PI_abs_ in W18 and Sw-1 under control and cold stress conditions. The data are presented as the mean ± SD of three replicates. Bars with different letters are significantly different at *P* < 0.05 (ANOVA followed by Tukey’s test)

The phenotypes and physiological changes of two genotypes were significantly different under low temperature stress (Fig. 2). The REC and MDA content in W18 and Sw-1 were significantly increased under cold stress. However, the increase in W18 was smaller than that in Sw-1 (Fig. 2B, C). Under cold stress, the H_2_O_2_ content and O_2_·^−^ generation rate of the two genotypes were significantly increased compared to those under NT. In addition, the H_2_O_2_ content and O_2_·^−^ generation rate of W18 were lower than those of Sw-1 (Fig. 2D, E). At low temperature, the total antioxidant capacity (T-AOC) of both genotypes increased, but the T-AOC of W18 was higher than that of Sw-1 (Fig. 2F). Compared with the control condition, the PI_abs_ of Sw-1 declined, while the PI_abs_ of W18 had no significant change (Fig. 2G). These results indicated that W18 was more tolerant than Sw-1.

### Mapping and quantitative assessment of Illumina sequences

The two comparison groups were named CT (LT vs NT) and CS (LT vs NT). Twelve cDNA libraries (three per treatment group) were constructed from total RNA. As indicated in Table S1, among the million raw reads obtained from the libraries, approximately 580.21 million clean reads were identified, ranging from 46.63 million to 49.78 million reads per library. More than 92% of the clean reads had quality scores higher than the Q30 level (an error probability of 0.1%), and the GC content was approximately 48% in each sample. The qualitied clean reads of each library were mapped to the *B. rapa* reference genome, and the mapping rates in each library exceeded 89% (Table S2). All of the RNA-seq data in this article have been deposited in the NCBI Sequence Read Archive (SRA) database and are accessible under accession number PRJNA735896.

### Different transcriptome profiles of CT and CS under LT and NT conditions

After calculating the gene expression, we found that samples of CT and CS in NT had fewer genes than the samples in LT at the expression level of FPKM (the expected number of fragments per kilobase of transcripts per million mapped fragments) > 10, and CS was less than CT in LT (Fig. S1A). The results of the expression density distribution were similar (Fig. S1B). The relationship among the samples was analyzed by principle component analysis (PCA). The results showed that samples in the same treatment in each genotype clustered together, and there was significant distinction between the samples of the two genotypes and the different treatments (Fig. S1C).

### DEG identification and analysis

**Fig. 3:**
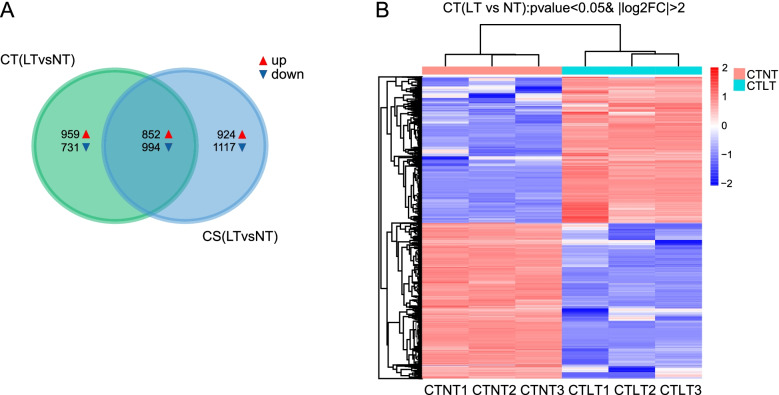
Analysis of DEGs in CT and CS. **A**: Number of DEGs in the pairwise group. The red indicates upregulated genes, while the blue indicates downregulated genes. **B**: Hierarchical clustering DEGs in CT (LT vs NT), each column represents a comparison group, and each row represents a gene

There were 3536 DEGs in CT (LT vs NT) and 3887 DEGs in CS (LT vs NT). Although there were more DEGs in CS (LT vs NT) than in CT (LT vs NT), there were more downregulated DEGs in CS (LT vs NT) than in CT (LT vs NT). A total of 1846 DEGs (852 up-regulated DEGs and 994 down-regulated DEGs) were shared between CT (LT vs NT) and CS (LT vs NT), and 1690 DEGs were specific to CT (LT vs NT) (Fig. 3A). All DEGs were hierarchically clustered with respect to gene expression patterns and were evaluated using log_10_RPKMs (reads per kilobase per million mapped reads) of the two groups (Fig. 3B and Fig S2).

Although different with respect to cold tolerance, the two groups shared common genes that regulated cold tolerance, including dehydration-responsive element-binding proteins (*DREB*s), cold-regulated protein (*COR15A*), *GST*s, and beta-glucosidase (*BGLU*) (Table S3). Several transcription factors, such as bHLH61, WRKY25, and MYB29 were also identified in both groups.

### GO enrichment analysis of DEGs

**Fig. 4:**
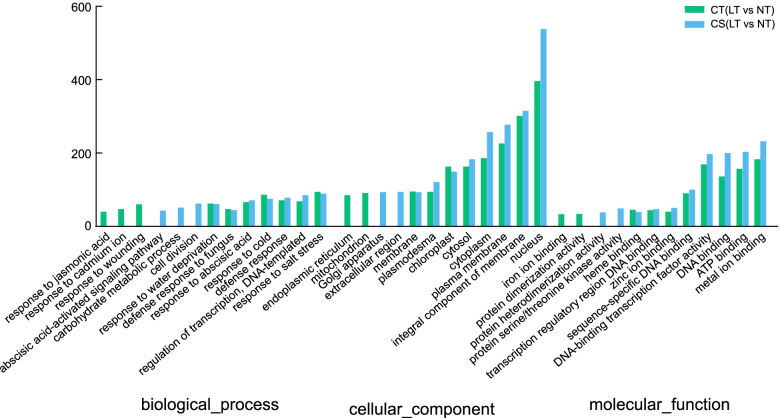
GO distribution of DEGs under 24-h cold stress in the two groups. The cold tolerance-related DEGs were based on GO categories that were grouped into three levels: biological process, cellular component, and molecular function. the left y-axis shows the number of genes, and the x-axis indicates specific categories of genes

Based on the corrected *P*-values, we selected the 10 most enriched GO terms in each category (Fig. 4, Table S4 and Table S5). In the biological process category, response to salt stress (GO:0,009,651), response to cold (GO:0,009,409), and defense response (GO:0,006,952) were common in both groups, and response to cadmium ion (GO:0,046,686), response to jasmonic acid (GO:0,009,753), and response to wounding (GO:0,009,611) were unique to CT (LT vs NT). In the cellular component, common DEGs were significantly enriched in the nucleus (GO:0,005,634), the integral component of membrane (GO:0,016,021), and the plasma membrane (GO:0,005,886), whereas mitochondrion (GO:0,005,739) and endoplasmic reticulum (GO:0,005,783) were enriched only in CT (LT vs NT). Metal ion binding (GO:0,046,872), DNA-binding transcription factor activity (GO:0,003,700), ATP binding (GO:0,005,524), and DNA binding (GO:0,003,677) were the top four in the molecular function category, and protein dimerization activity (GO:0,046,983) and iron ion binding (GO:0,005,506) were enriched only in CT (LT vs NT).

### KEGG pathway enrichment analysis of DEGs

**Fig. 5:**
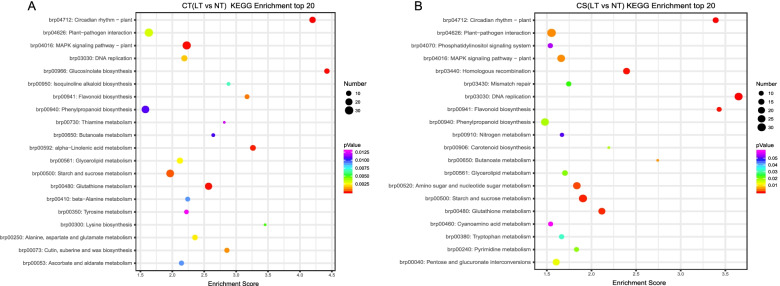
KEGG enrichment analysis with the 20 most enriched KEGG terms in CT (LT vs NT) (**A**) and CS (LT vs NT) (**B**). High and low *P*-values are represented by blue and red, respectively

To understand the function of DEGs, we mapped the DEGs to the reference specification path in the KEGG database. The core genes were aligned with the KEGG database and were assigned to 20 KEGG pathways. KEGG pathway enrichment results showed that the significantly enriched KEGG pathways in both groups were starch and sucrose metabolism (brp00500) and glutathione metabolism (brp00480) (Fig. 5A, B). Based on a comparison of the KEGG pathways of CT (LT vs NT) and CS (LT vs NT), we were interested in pathways with higher enrichment in CT but lower enrichment in CS. One such way was α-linolenic acid metabolism.

### DEGs involved in common KEGG pathways in CT (LT vs NT) and CS (LT vs NT)

**Fig. 6:**
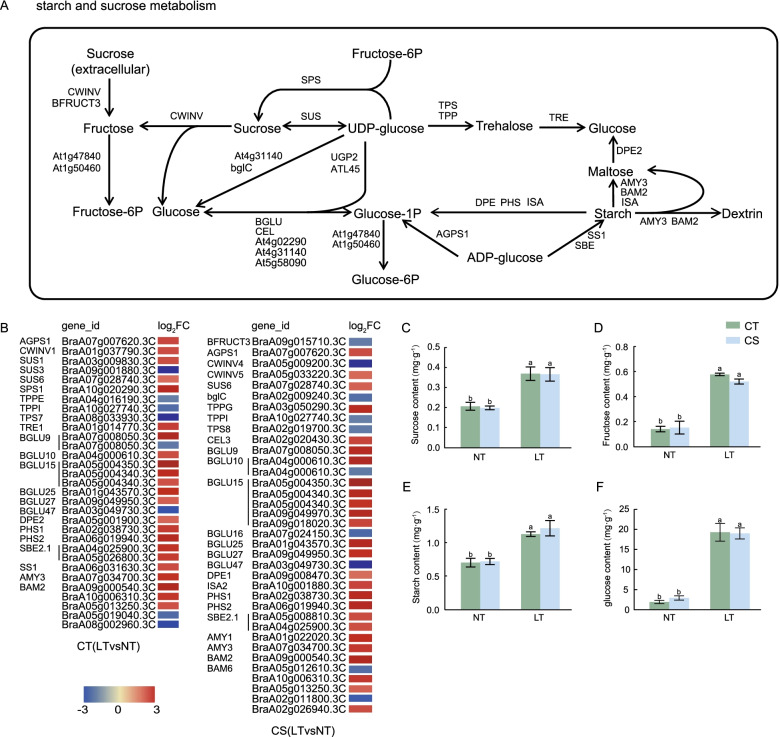
DEGs relevant to the starch and sucrose metabolism pathway and carbohydrate content of the two wucai genotypes under cold stress. **A**: pathway diagram of starch and sucrose metabolism. **B**: The heat map of the expression of DEGs related to the starch and sucrose metabolism pathway in CT (LT vs NT) and CS (LT vs NT). **C**: Starch content of CT and CS under control and cold stress conditions. **D**: Sucrose content of CT and CS under control and cold stress conditions. **E**: Fructose content of CT and CS under control and cold stress conditions. **F**: Glucose content of CT and CS under control and cold stress conditions

The starch and sucrose metabolism network changed significantly in the acclimation to cold stress of both groups (Fig. 6A). We identified 31 DEGs in CT (LT vs NT) and 36 DEGs in CS (LT vs NT), with 23 and 26 up-regulated DEGs, respectively. The expressions of beta-amylase 2 (*BAM2*), sucrose-phosphate synthase1 (*SPS1*), *BGLUs*, and *BraA01g006680.3C* were the most significant in CT (LT vs NT) (Fig. 6B); these genes are mainly involved in the transformation of fructose, sucrose, and glucose and the hydrolysis of starch to maltose and finally to glucose. The starch, fructose, sucrose, and glucose contents of CT and CS increased significantly under cold stress, and there was no significant difference between the two groups (Fig. 6C-F).

**Fig. 7:**
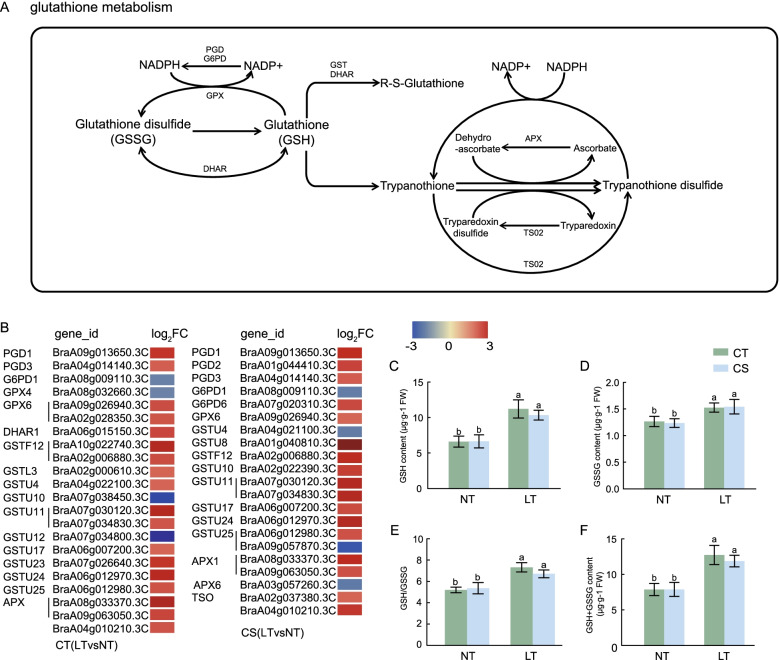
DEGs relevant to the glutathione metabolism pathway and intermediate metabolites of the two wucai genotypes under cold stress. **A**: Pathway diagram of the glutathione metabolism. **B**: The heat map of the expression of DEGs related to glutathione metabolism pathway CT (LT vs NT) and CS (LT vs NT). **C**: GSH content under control and cold stress conditions. **D**: GSSG content under control and cold stress conditions. **E**: GSH/GSSG under control and cold stress conditions. **F**: Sum of the GSH and GSSG contents under control and cold stress conditions

In glutathione metabolism, 22 DEGs in CT (LT vs NT) and 21 DEGs in CS (LT vs NT) were identified, with 18 and 17 up-regulated genes, respectively (Fig. 7A, B). Ascorbate peroxidase 1 (*APX1*), phospholipid hydroperoxide glutathione peroxidase 6 (*GPX6*), and most of the GST gene family members were up-regulated. Compared with NT, the contents of reduced glutathione (GSH), oxidized glutathione (GSSG), and total glutathione (GSH + GSSG) in CT and CS were significantly increased under cold stress. The GSH content was increased by 69.86% and 55.54%, respectively, and the GSSG content was increased by 20.66% and 24.89%, respectively. The (GSH + GSSG) content increased in the two groups by 61.94% and 50.73%, respectively. The glutathione content and GSH/GSSG ratio under cold stress were higher than those of the control (Fig. 7C-F).

### DEGs involved in the KEGG pathway specific to CT (LT vs NT)

**Fig. 8:**
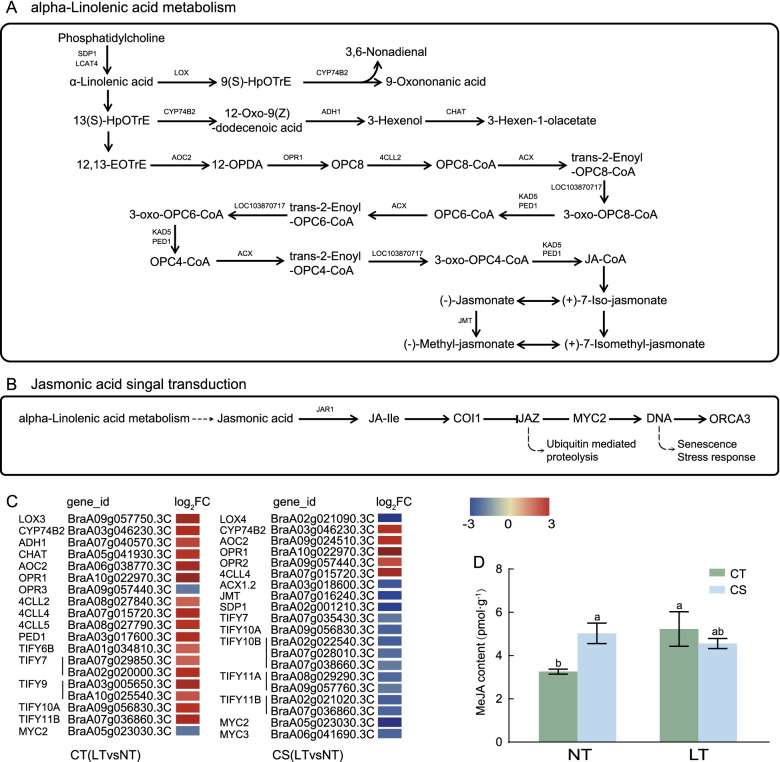
DEGs relevant to the α-linolenic acid metabolism pathway and JA signal transduction pathway in CT (LT vs NT) and CS (LT vs NT). **A**: Pathway diagram of the α-Linolenic acid metabolism. **B**: Pathway diagram of jasmonic acid signal transduction. **C**: The heat map of the expression of DEGs related to α-Linolenic acid metabolism pathway and jasmonic acid signal transduction pathway. **D**: MeJA content of CT and CS under control and cold stress conditions

KEGG enrichment analysis showed that the α-linolenic acid metabolism pathway was enriched in the CT group. Based on the metabolism pathway and heat map analysis (Fig. 8A, C), we found that 4-coumarate–CoA ligase (*4CLL*), allene oxide cyclase (*AOC*), and 12-oxophytodienoate reductase (*OPR*) were up-regulated in both groups, but the key gene, *LOX*, was up-regulated only in CT. JA is catabolized by JA carboxyl methyltransferase (JMT) to form its volatile counterpart MeJA. JMT in CS was down-regulated. The above GO enrichment analysis showed that the response to jasmonic acid was enriched only in CT; therefore, we studied the jasmonic acid signal transduction pathway (Fig. 8B), and the results showed that members of JAZ (TIFYs), an important gene family in this pathway, were up-regulated in CT (LT vs NT) and down-regulated in CS (LT vs NT) (Fig. 8C). Compared with NT, the MeJA content in CT under LT increased by 60.33%, while it was down-regulated by 9.38% in CS (Fig. 8D).

### Identification of transcription factors in response to cold stress

**Fig. 9:**
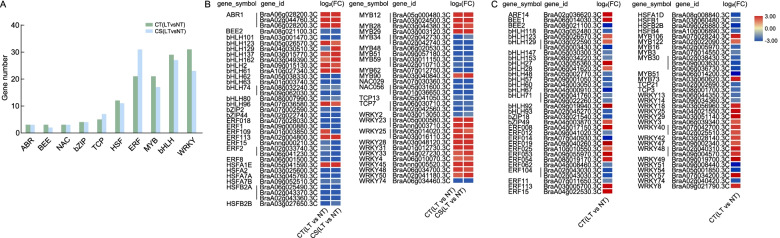
Statistical analysis of TFs under cold stress. **A**: Numbers of top 10 TF families in CT (LT vs NT) and CS (LT vs NT). **B**: The heatmap of TFs common to the two groups. **C**: The heatmap of TFs unique to CT (LT vs NT)

The RNA-seq results showed that a total of 206 and 208 DEGs were annotated as TFs in CT (LT vs NT) and CS (LT vs NT), and were classified into 47 and 48 families, respectively. The top 10 TF families are shown in Fig. 9A. Among the recognized TF families, WRKY and bHLH represented the most abundant category, followed by MYB and ERF in CT (LT vs NT) and CS (LT vs NT). The trend of the fold change of common TFs between the two groups was similar. In the TFs unique to CT (LT vs NT), bHLH and WRKY were the top two TF families; most of the bHLHs were down-regulated and the WRKYs were up-regulated (Fig. 9B, C).

### Validation of gene expression patterns by qRT-PCR

**Fig. 10:**
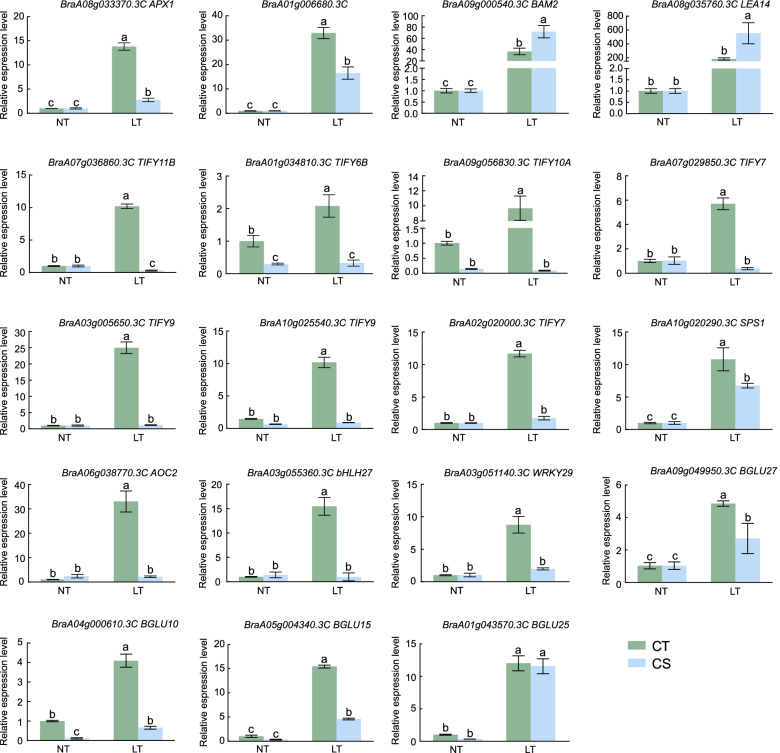
Validation of gene expression patterns in CT and CS under cold stress by qRT-PCR. qRT-PCR analysis of 19 selected DEGs. Bars with different letters are significantly different at *P* < 0.05 (ANOVA followed by Tukey’s test)

To validate the data obtained from RNA-seq, qRT-PCR was performed. Nineteen DEGs were selected for qRT-PCR, including two transcription factors (one bHLH and one WRKY), seven members (TIFYs) of the *JAZ* gene family, seven genes involved in starch and sucrose metabolism (*BraA01g006680.3C*, *BAM2*, *SPS1*, *BGLUs*), and three other genes (Fig. 10). The qRT-PCR results were strongly correlated with RNA-seq data for both CT (*R*^*2*^ = 0.8202) (Fig. S3A) and CS (*R*^*2*^ = 0.8230) (Fig. S3B), demonstrating the reliability of the RNA-seq expression profile.

## Discussion

In order to resist cold, plants change their morphological, physiological and biochemical properties, which involve molecular changes [24]. In this study, we used the RNA-seq technique to analyze the transcriptome of two wucai genotypes after 24 h cold treatment and 24 h normal treatment to determine the response to cold stress. The DEGs common to both genotypes and unique to CT (LT vs NT) were identified and further analyzed, which could provide insights into the candidate genes and metabolic pathways underlying cold tolerance in wucai.

Based on our team’s research results, six genotypes of wucai were screened for tolerance, and two divergent varieties were chosen for further study. REC was negatively correlated with plasma membrane integrity and was related to the strength of plant stress resistance [25, 26]. MDA is a marker to measure lipid peroxidation in plant cells, which is often used to evaluate plant tolerance to biological or abiotic stresses [27]. REC and MDA are commonly used to measure membrane damage and cell stability [28]. Plant photosynthesis is very sensitive to cold stress, and PI_abs_ is a multi-parametric expression that combines the three main functional steps taking place in PSII (light energy absorption, excitation energy trapping, and conversion of excitation energy to electron transport). PI_abs_ reflect the capture of light energy by PSII reaction center and the ability of photosynthetic electron transfer between the two photosystems [29]. PI_abs_ could be used to select individuals for analysis or to screen wucai genotypes for enhanced cold tolerance, and it is positively correlated with the cold tolerance of plants [23]. The change of relative variable fluorescence V_j_ reflects the transfer of electrons on the PSII electron receptor side from QA to QB. The change of V_j_ further showed that PSII receptor was affected under cold stress. V_j_ reflects the photosynthetic capacity which is negatively correlated with the photosynthetic capacity of plants [20]. The changes of these indexes in wucai were consistent with the above research.

A previous study reported that ROS has a dual function in plants [6]. Under normal conditions, the intracellular ROS level is low, which regulates plant growth and development and stress responses [30]. Under abiotic stress, ROS accumulates and has a toxic effect on cells [31]. The H_2_O_2_ content and O_2_·^−^ generation rate of CS were higher than those of CT under cold stress (Fig. 2D, E). The T-AOC is one of the important indexes reflecting the capacity of the non-enzymatic intracellular antioxidant defense system [32]. The tolerance of species and genotypes to abiotic stress is related to the antioxidant capacity of leaves [33]. The increase in the T-AOC of CT was greater than that of CS under cold stress (Fig. 2F). It was consistent with research results on the resistance of Brassica napus to salt and cold stress, and T-AOC might be a potentially useful phenotypic marker of stress resistance [34]. These results indicated that CT has a stronger ability to protect cells from toxicity and has a stronger defense against cold damage. There was no significant change of PI_abs_ in CT, while a significant decrease was observed in CS (Fig. 2E), indicating that the photosynthetic mechanism of CS was damaged. The changes in these parameters are consistent with W18 having greater cold-tolerance than Sw-1.

Gene function and expression studies are needed to identify the key genes for cold resistance in wucai. Based on a comparison of the DEGs of the two groups, the number of DEGs in CS (LT vs NT) was higher, while the number of up-regulated DEGs in CS (LT vs NT) was fewer than that in CT (LT vs NT) (Fig. 3A). In the DEGs specific to CT (LT vs NT), 959 genes were up-regulated, including TIFY, calmodulin, LOX, and transcription factors, WRKY and MYB, which might be involved in greater cold tolerance in CT. GO enrichment specifically in CT included the response to cadmium ion (GO:0,046,686), response to jasmonic acid (GO:0,009,753), and response to wounding (GO:0,009,611). JA and its derivatives play an important role in the resistance to abiotic stress [35]. Up-regulated DEGs in response to cadmium ion, such as APX1 and GPX6, have been reported to participate in cold response in Brassica juncea by scavenging ROS [36]. The DEGs involved in these three GO might participate in cold tolerance in wucai.

A large number of studies have confirmed that low-temperature stress can induce the accumulation of soluble sugars to response to stress tolerance [37, 38]. Low temperature also promotes increased levels of enzymes, such as sucrose-phosphate synthase (SPS), sucrose synthase (SUS), and invertase (INV), which ultimately increased sucrose levels [39]. The accumulation of soluble sugar in rice and cold-treated *Arabidopsis thaliana* [40] is associated with cold tolerance. The key genes involved in the conversion of fructose, sucrose, and glucose, such as *SPS1*, *SUS6*, beta-fructofuranosidase, insoluble isoenzyme 5 (*CWINV5*), and *BGLU*s, were up-regulated in CT and CS (Fig. 6A, B). In both comparison groups, starch synthesis was not inhibited (Fig. 6C, D), and a significant trend was observed that polysaccharides were degraded to disaccharides and soluble, simple sugars. Starch is hydrolyzed/decomposed into maltose/dextrin by alpha amylase 3 (*AMY3*) and *BAM2* and is then hydrolyzed into glucose by 4-alpha-glucanotransferase (*DPE2*). It was reported that sucrose and starch metabolism was significantly enriched and the content of soluble sugar was significantly increased after cold stress in Poa pratensis with different cold tolerance [41]. Poa pratensis is a cold tolerant species, and this result was consistent with ours. It might indicate that starch and sucrose metabolism was involved in the response of wucai with different cold tolerance to cold stress.

GSH is involved in ROS clearance in the non-enzymatic mode, but the possibility of direct ROS clearance by GSH is considered low [42]. GSH participates in the ROS scavenging enzymatic reaction by regulating the activities of related enzymes in the antioxidant enzyme system (APX, mono dehydroascorbate reductase, dehydroascorbate reductase, and glutathione reductase) and by indirectly scavenging ROS. In this study, *APX1* was significantly up-regulated in the glutathione metabolism pathway in both CT (LT vs NT) and CS (LT vs NT) (Fig. 7B). GSH is mainly involved in regulating the response of GPX (phospholipid hydroperoxide glutathione peroxidase) and GST. GPX is a member of the proline oxidase antioxidant enzyme family, which uses GSH to remove H_2_O_2_ and reduce the accumulation of lipids and organic hydroperoxides [43]. GST can also be coupled with GPX to activate GPX and participate in H_2_O_2_ scavenging. One transcription (*BraA09g026940.3C*) of *GPX6* was up-regulated in both groups, and the other transcription (*BraA08g032660.3C*) was down-regulated in CT (LT vs NT) but was not detected in CS (LT vs NT). Members of the *GST* family, specifically *GSTU11*, *GSTU24*, and *GSTF12*, were significantly up-regulated in both groups. These results suggested that the glutathione metabolism pathway, as an important component of the antioxidant process in plants, might play a role in both genotypes of wucai in response to cold stress. This may be a reason why wucai is a semi-hardy species.

*LOX* initiates the first step of α-linolenic acid oxidation, JA, and MeJA synthesis [44]. It has been reported that there are 13 *LOX* genes in *Brassica rapa*, and *LOX* gene participate in the response to stress [45]. In the present study, *LOX3* was differentially expressed and up-regulated in CT. *LOX4* was differentially expressed in CS and down-regulated (Fig. 8C). MeJA alleviates low temperature stress in tomato [46] and cowpea (*Vigna sinensis*) [47] by increasing antioxidant synthesis and the activity of some defense compounds. JMT is a key enzyme involved in the conversion of JA to MeJA [48]. It was down-regulated in CS and was not detected in CT. The MeJA content in CT was significantly increased compared with CS, indicating that MeJA might have a stronger response to cold stress than CS (Fig. 8D). JA-mediated signal transduction pathways play an important role in various stress responses [49]. Previous studies have shown that JA acts as a key upstream signal to regulate the ICE-CBF/DREB1 transcription pathway to prevent frost damage in *Arabidopsis thaliana* [50]. TIFY is a plant-specific transcription factor family that includes four subfamilies, TIFY, JAZ, PPD, and ZML. Studies have shown that members of the *JAZ* subgroup are involved in the response to abiotic stress by participating in the JA signal transduction pathway [51], and MeJA induces a defense response by activating JA-related genes, thus endowing pepper with tolerance to chilling injury [52]. And *JAZ*s encode the plant-specific proteins that are involved in JA signaling and stress tolerance [53]. In this study, *JAZ* genes identified in CT (LT vs NT) were up-regulated and were down-regulated in CS (LT vs NT) (Fig. 8C). MYC2 regulates downstream transcription factors in JA signal transduction, and its downregulation in CT is lower than that in CS. Therefore, we hypothesize that α-linolenic acid metabolism and the signal transduction pathway of JA might be the main reasons for the difference in cold tolerance between CT and CS. Thus, the molecular mechanisms of these JA-related genes need to be further investigated.

TFs are important upstream regulators that are key to the regulation of gene expression in plants under abiotic stress [54, 55]. Previous studies have reported that overexpression of bHLH92 can improve the cold tolerance of Arabidopsis thaliana [56]. The induction of AtERF53 [11] and expression of WRKY53 and WRKY54 [57] increased biotic and abiotic stress tolerance in transgenic Arabidopsis. These TFs were identified only in CT, indicating that that they may play a role in the cold tolerance of wucai (Fig. 9B). Thus, future research could focus on the role of these transcription factors in metabolic pathways to understand the regulatory mechanism of genes in wucai.

## Conclusions

In this study, through phenotypic observation and physiological index measurements, we identified the cold-tolerant (W18) and cold-sensitive (Sw-1) genotypes among the six wucai genotypes. Transcriptome analysis was used to screen DEGs related to different cold tolerance levels of wucai, which has a certain reference value for cold-tolerance breeding of wucai. DEGs in the two groups might response to cold stress by participating in starch and sucrose metabolism and glutathione metabolism. The cold tolerance of CT was mainly related to α-linolenic acid metabolism and the JA signal transduction pathway and involved transcription factors, such as bHLH92, ERF53, WRKY53, and WRKY54. These findings provide genetic resources and a theoretical basis for subsequent studies on the improvement of gene function and cold tolerance of wucai and also have a certain reference value for the analysis of the cold tolerance of wucai and other similar species of winter rapeseed (*Brassica juncea* and *Brassica napus*).

## Methods

### Plant material and treatment

We selected six wucai genotypes, ws-1, ws-2, Sw-1, Sw-3, W18, and Ta2, which were provided by the Vegetable Genetics and Breeding Laboratory of Anhui Agricultural University. Seeds were sown in plug trays and then transplanted into pots containing substrate and vermiculite with a volume ratio of 2:1. Seedlings were grown in a growth chamber at 25 °C (day) and 15 °C (night) with 300 μmol·m^−2^·s^−1^ photon flux density and 70% relative humidity under a 16 /8 h (light/night) photoperiod. For cold treatment, seedlings at the 7-leaf stage were transferred to the following conditions: 9 °C (day) and 4 °C (night) with 300 μmol·m^−2^·s^−1^ photon flux density and 70% relative humidity under a 16 /8 h light/dark photoperiod, whereas the control plants were cultured under the normal environmental conditions mentioned above. After 3 d cold treatment, biochemical parameters, such as REC, the MDA content, PI_abs_, and V_j_, were measured and compared with the control.

### Measurement of MDA, REC, O_2_·^−^, and H_2_O_2_

The MDA content was assessed following the methods previously described by Mohammadi et al. with minor modifications [58]. Fresh leaves (0.2 g) were homogenized in 2 mL 10% trichloroacetic acid (TCA) and centrifuged at 4000 rpm for 10 min. Then, 2-mL supernatant was mixed with 2 mL thiobarbituric acid (TBA) (0.6%), heated at 100 °C for 15 min, and cooled at room temperature. The absorbance of each aliquot was measured at 450, 532, and 600 nm. The MDA content was calculated using the equation:

MDA (μmol·g^−1^ FW) = 6.45 × (A_532_ − A_600_) − 0.56 × A_450_.

Relative electrolyte leakage was determined according to the method reported by Bajji et al. with minor modifications [59]. The leaves were perforated with a hole punch with a radius of 9.5 mm. Three discs were placed in a 20 mL tube containing 10 mL of ultrapure water and then placed in a thermostated water bath at 25 °C for 30 min. After this, the conductivity (EC1) of water was measured using a Thermo OrionSTARA HB conductivity meter (Thermo Orion., Waltham, MA, USA). The tube was heated in boiling water for 30 min, then cooled to room temperature, and the conductivity (EC2) was measured again. The final REC was equal to the percentage of EC1/EC2.

The O_2_·^−^ generation rate and the H_2_O_2_ content were measured using Solarbio reagent kits (Cat#BC3595 and Cat#BC1290, Beijing Solarbio Science & Technology Co. Ltd, Beijing, China).

### Analysis of Chlorophyll Fluorescence Parameters

PI_abs_ = (RC/ABS) • [φ_Po_/(1–φ_Po_)] [ψ_o_/(1–ψ_o_)] and is a performance index with an absorption basis. V_j_ = (F_j_–F_o_)/(F_m_–F_o_), which is the relative variable fluorescence intensity at the J-step. To measure PI_abs_ and V_j_, the leaves were dark-adapted for 30 min by special clips before being illuminated with saturating light (1 s), after which they were measured using a continuous excitation fluorometer Pocket Plant Efficiency Analyzer (Pocket PEA, Hansatech, UK).

### RNA extraction, cDNA library construction, and sequencing

A total of four group samples [two materials (leaves of W18 and Sw-1) under two treatments (control and 10 °C/4 °C 24 h)] with three biological replications were used for RNA-seq. The total RNA of samples mentioned above was extracted using a mir-Vana miRNA isolation kit (Ambion, TX, USA) according to the manufacturer’s instructions. The total RNA was quantified using an Agilent 2100 Bioanalyzer (Agilent Technologies, Santa Clara, CA, USA). Samples with an RNA Integrity Number ≥ 7.0 and 28S/18S ratio ≥ 0.7 were subjected to subsequent analysis. The libraries were constructed using the TruSeq Stranded mRNA LT Sample Prep Kit (Illumina, San Diego, CA, USA) following the manufacturer’s instructions. Then, these libraries were sequenced on the Illumina HiSeqTM 2500 platform (BiomarkerBiotech, Beijing, China) to generate 125 bp/150 bp paired-end reads.

### Sequence assembly, annotation, and identification of the DEGs

Clean reads were obtained by removing adaptor sequences, more than 10% N bases, and low quality (Q ≤ 20) reads with more than 50% bases. The reads were mapped to the *B. rapa* reference genome [60] by hisat2 [61]. Read counts per gene were expressed as FPKM, and unigene abundance differences between the samples were calculated based on the ratio of the FPKM values and false discovery rate (FDR). DESeq software [62] was used to standardize the counts of each sample gene (use basemean value to estimate the expression), calculate the difference multiple, and use NB (negative binomial distribution test) to test the difference significance of the reads number. Finally, screen the differential protein coding genes according to the difference multiple and difference significance test results. Genes with FDR ≤ 0.05 and |log_2_ (Fold Change) |≥ 2 were considered DEGs.

### Enrichment analysis

Hierarchical clustering of the DEGs was analyzed by HemI software [63]. Statistical enrichment of the DEGs was carried out in KEGG pathways using KOBAS software [64], and log_2_ (Fold Change) values were used in the gene expression heatmap.

### Measurement of carbohydrate, glutathione metabolism products, and MeJA content

The contents of sucrose, fructose, and starch were determined by anthrone colorimetry [65] with minor modifications. Glucose, GSH, and GSSG contents were measured using a biochemical reagent kit (Cat#BC2500, Cat#BC1175, and Cat#BC1180, respectively; Beijing Solarbio Science & Technology Co. Ltd, Beijing, China). MeJA was determined by enzyme-linked immunosorbent assay.

### Identification of transcription factors

TFs were identified by analyzing InterProScan domain patterns in sequences with high coverage, and the sensitivity was analyzed using PlantTFcat tools [66].

### qRT-PCR validation

A total of 15 transcripts were selected to verify the RNA-seq analysis. The transcript-specific primers used in this study were designed using Primer6 and were then synthesized by General Biosystems (Chuzhou, China). The primers used for qRT-PCR are listed in Table S6. The qRT-PCR reactions were performed with the AceQ qPCR SYBR GREEN Master Mix (Vazyme Biotechnology Co., Nanjing, China). *BnaActin* [21] was selected as the internal standard to calculate the relative expression levels as follows: FC = 2^−△△CT^ [67]. Three biological repeats for each sample for each gene were performed.

## Supplementary Information

**Additional file 1: Fig. S1.** Regional distribution, density distribution and principal component analysis of gene expression. **Fig. S2.** Hierarchical clustering DEGs in CS (LTvsNT). Each column represents a comparison group, and each row represents a gene.** Fig. S3.** qRT-PCR validation of expression profiles obtained by RNA-Seq in CT and CS under cold stress.** Table S1.** Summary of sequence assembly after illumine sequencing.** Table S2.** Number of reads sequenced and mapped to the *Brassica rapa* genome.** Table S3.** List of the core genes set involved in CS response to cold stress.** Table S4.** Top30 up GO enrichment analysis in CT(LTvsNT).** Table S5.** Top30 up GO enrichment analysis in CS(LTvsNT).** Table S6.** Primer pairs used to detect the expression of selected genes.

## Data Availability

The raw RNA-Seq datasets are available in the Sequence Read Archive of National Center for Biotechnology Information (https://dataview.ncbi.nlm.nih.gov/object/PRJNA735896?reviewer=fnp3ih4ojrq5ohq5vmstrr5koe; accession number: PRJNA735896).

## Abbreviations

OH: Hydroxyl radicals
4CLL: 4-Coumarate–CoA ligase
AMY3: Alpha-amylase 3
AOC: Allene oxide cyclase
APX: Ascorbate peroxidase
BAM: Beta-amylase 2
BGLU: Beta-glucosidase
bHLH: Basic helix-loop-helix
CBF: C-repeat binding factor
COR15A: Cold-regulated protein
CS: Cold-sensitive
CSLT: Cold sensitivity in low temprature
CT: Cold-tolerant
CTLT: Cold tolerance in low temprature
CWINV: Beta-fructofuranosidase, insoluble isoenzyme
CYP75B1: Cytochrome P450 superfamily protein
DEGs: Differentially expressed genes
DPE2: 4-Alpha-glucanotransferase
DREB1: Dehydration-responsive element-binding proteins 1
ERF: Ethylene-responsive transcription factor
FDR: False discovery rate
FPKM: The expected number of fragments per kilobase of transcripts per million mapped fragments
GO: Gene ontology
GPX: Phospholipid hydroperoxide glutathione peroxidase
GSH: Reduced glutathione
GSSG: Oxidized glutathione
GST: Glutathione S-transferase
H_2_O_2_: Hydrogen peroxide
JA: Jasmonic acid
MeJA: Methyl jasmonate;
JAZ: Jasmonate ZIM-domain
JMT: JA carboxyl methyltransferase
KEGG: Kyoto encyclopedia of genes and genomes
KIN2: Serine/threonine protein kinase 2
LOX: 13S lipoxygenase
LT: Low temperature
MDA: Malondialdehyde
MeJA: Methyl jasmonate
NT: Control
O_2_·^−^: Superoxide anions
O_2_^1^: Singlet oxygen
OPR: 12-Oxophytodienoate reductase
PCA: Princinple component analysis
PCC: Pearson correlation coefficient
PHS: Alpha-glucan phosphorylase
PI_abs_: The performance index on an absorption basis
qRT-PCR: Quantitative real-time PCR
REC: Relative electrolyte conductivity
RNA-seq: RNA-sequencing
ROS: Reactive oxygen species
RPKM: Reads per kilobase per million mapped reads
SPS: Sucrose-phosphate synthase
SUS: Sucrose synthase
T-AOC: Total antioxidant capacity
TF: Transcription factor
TBA: Thiobarbituric acid
