# Supplementary material for: Transcriptome analysis and differential gene expression profiling of wucai (Brassica campestris L.) in response to cold stress
Source: BMC Genomics. 2022 Feb 15;23:137. doi: 10.1186/s12864-022-08311-3 (PMC8848729; doi:10.1186/s12864-022-08311-3)
Supplement: Supplementary file 1 — Additional file 1: Fig. S1. Regional distribution, density distribution and principal component analysis of gene expression. Fig. S2. Hierarchical clustering DEGs in CS (LTvsNT). Each column represents a comparison group, and each row represents a gene. Fig. S3. qRT-PCR validation of expression profiles obtained by RNA-Seq in CT and CS under cold stress. Table S1. Summary of sequence assembly after illumine sequencing. Table S2. Number of reads sequenced and mapped to the Brassica rapa genome. Table S3. List of the core genes set involved in CS response to cold stress. Table S4. Top30 up GO enrichment analysis in CT(LTvsNT). Table S5. Top30 up GO enrichment analysis in CS(LTvsNT). Table S6. Primer pairs used to detect the expression of selected genes. [file 12864_2022_8311_MOESM1_ESM.docx]

**Transcriptome analysis and differential gene expression profiling of wucai (*Brassica campestris* L.) in response to cold stress**

Chenggang Wang ^a, b, c, 1^, Mengyun Zhang ^a, 1^, Jiajie Zhou ^a^, Xun Gao ^a^, Shidong Zhu ^a, b, c^, Lingyun Yuan ^a, b, c^, Xilin Hou ^d^, Tongkun Liu ^d^, Guohu Chen ^a, b, c^, Xiaoyan Tang ^a,^ ^b, c^, Guolei Shan ^a, b^, Jinfeng Hou ^a, b, c, *^

a College of Horticulture, Vegetable Genetics and Breeding Laboratory, Anhui Agricultural University, 130 West Changjiang Road, 230036 Hefei, Anhui, China

b Provincial Engineering Laboratory for Horticultural Crop Breeding of Anhui, 130 West of Changjiang Road, 230036 Hefei, Anhui, China

c Wanjiang Vegetable Industrial Technology Institute, Maanshan, Anhui, 238200, China

d Department of Horticulture, Nanjing Agricultural University, 210095 Nanjing, Jiangsu, China

1 These authors contributed equally to this work.

* Corresponding author: Jinfeng Hou

E-mail: houjinfeng@ahau.edu.cn

Tel./Fax: +86 0551-65786212


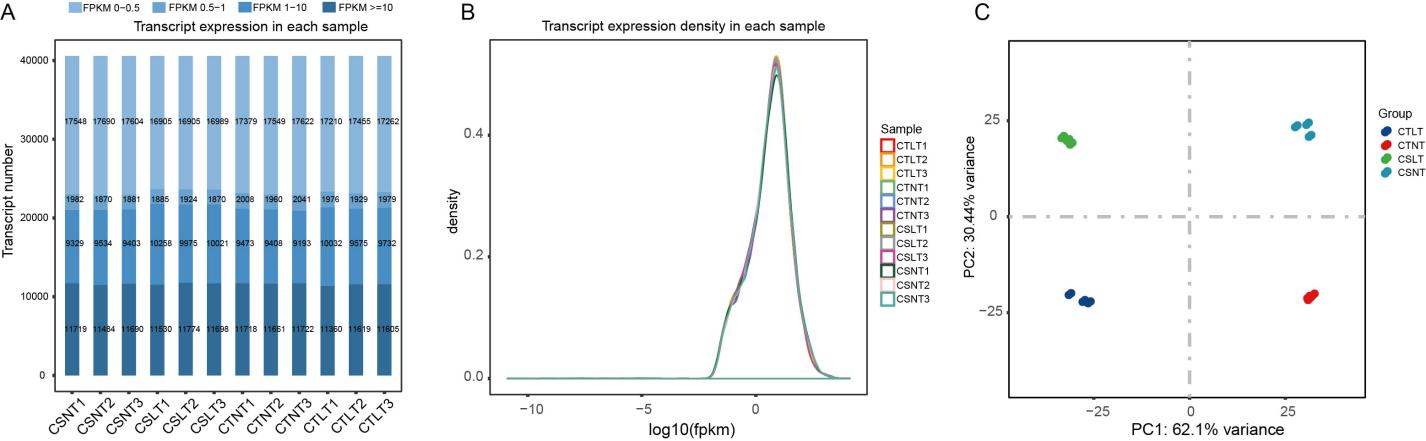


**Fig. S1.** Regional distribution, density distribution and principal component analysis of gene expression. A: Different colors represent different ranges of FPKM values, the horizontal axis is the sample, and the vertical axis is the number of protein-coding genes. B: The curves of different colors in the figure represent different samples, the abscissa of the points on the curve represents the logarithm of the corresponding sample FPKM, and the ordinate of the points represents the probability density. C: Different colors in the figure represent different samples. The closer the sample clustering distance or PCA distance is, the more similar the samples are. The samples of each group are distributed in different areas of two-dimensional space, and the samples of the same group are more concentrated in spatial distribution.

C
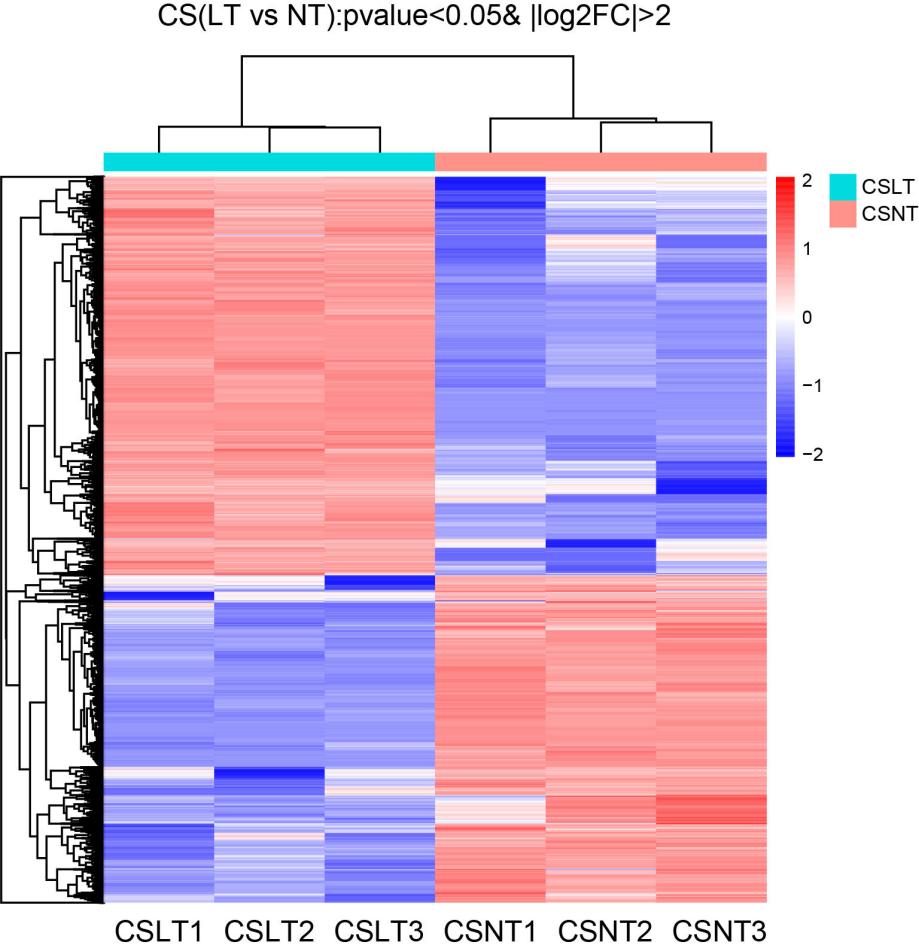


**
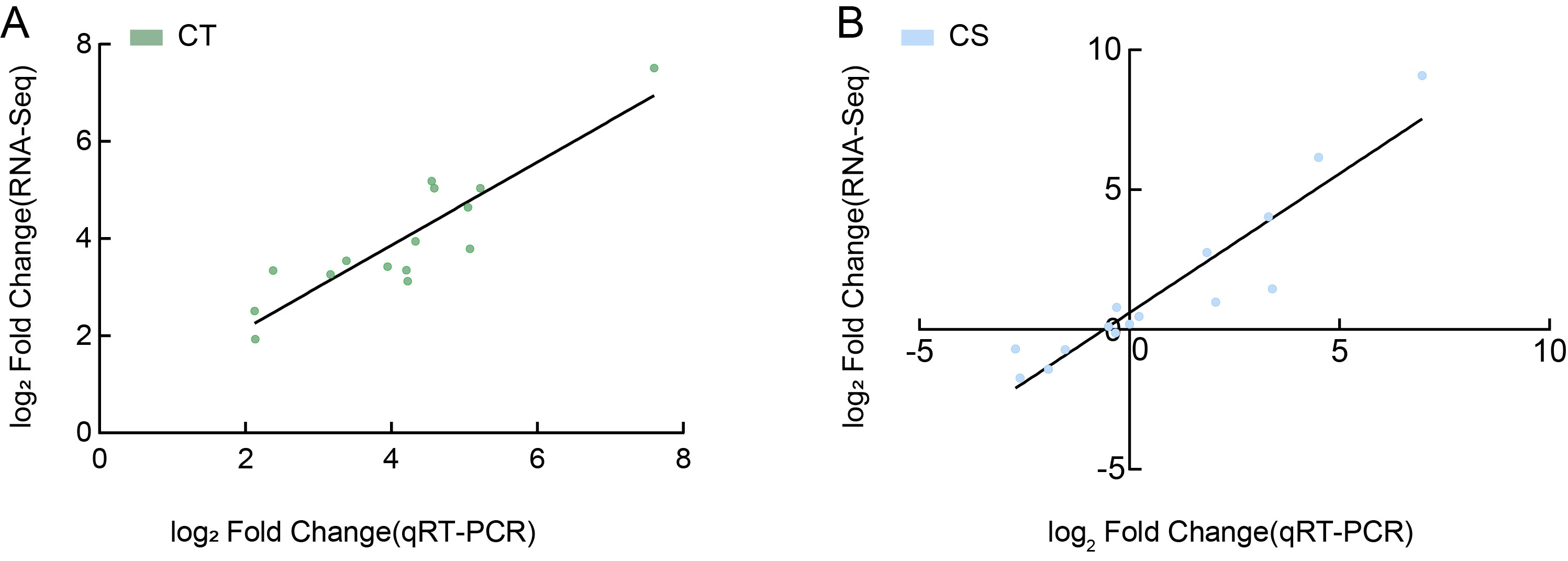
Fig. S2.** Hierarchical clustering DEGs in CS (LTvsNT). Each column represents a comparison group, and each row represents a gene. Red and blue represent high or low expression levels, respectively, than those shown in white.


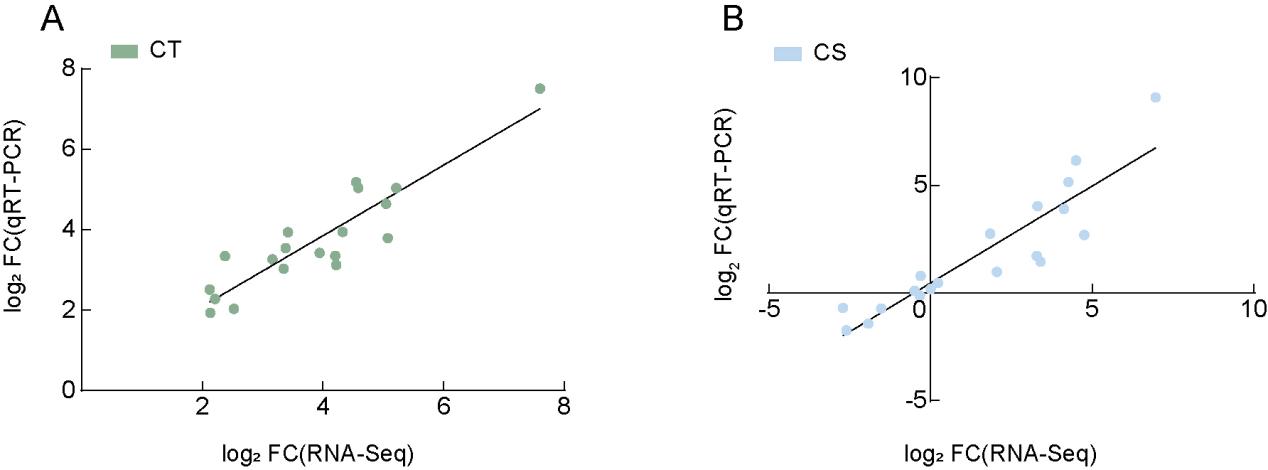


**Fig. S3.** qRT-PCR validation of expression profiles obtained by RNA-Seq in CT and CS under cold stress. A: Validation of gene expression patterns in CT. B: Validation of gene expression patterns in CS. Each point represents a value of fold change of expression level at LT with that at NT. The fold change values were converted by log_2_ standardization.

**Table S1** Summary of sequence assembly after illumine sequencing.

| **Sample** | **CTLT1** | **CTLT2** | **CTLT3** | **CTNT1** | **CTNT2** | **CTNT3** | **CSLT1** | **CSLT2** | **CSLT3** | **CSNT1** | **CSNT2** | **CSNT3** |
| --- | --- | --- | --- | --- | --- | --- | --- | --- | --- | --- | --- | --- |
| RawReads | 50.97M | 48.19M | 48.12M | 49.46M | 50.48M | 49.49M | 49.48M | 51.14M | 50.82M | 49.60M | 47.89M | 49.68M |
| RawBases | 7.65G | 7.23G | 7.22G | 7.42G | 7.57G | 7.42G | 7.42G | 7.67G | 7.62G | 7.44G | 7.18G | 7.45G |
| CleanReads | 49.66M | 46.93M | 46.94M | 48.21M | 49.24M | 48.38M | 48.17M | 49.78M | 49.61M | 48.42M | 46.63M | 48.24M |
| CleanBases | 6.87G | 6.53G | 6.56G | 6.70G | 6.88G | 6.78G | 6.71G | 6.80G | 6.84G | 6.79G | 6.55G | 6.75G |
| ValidBases | 89.89% | 90.31% | 90.92% | 90.33% | 90.82% | 91.33% | 90.39% | 88.68% | 89.76% | 91.21% | 91.18% | 90.54% |
| Q30 | 92.74% | 92.82% | 92.92% | 92.69% | 92.98% | 93.21% | 92.67% | 92.47% | 93.02% | 93.10% | 92.71% | 92.53% |
| GC | 48.44% | 48.50% | 48.39% | 48.44% | 48.30% | 48.32% | 48.03% | 48.48% | 48.47% | 48.00% | 47.87% | 47.85% |

**Table S2** Number of reads sequenced and mapped to the *Brassica rapa* genome.

| **Sample** | **NsLt1** | **NsLt2** | **NsLt3** | **NsNt1** | **NsNt2** | **NsNt3** | **SLt1** | **SLt2** | **SLt3** | **SNt1** | **SNt2** | **SNt3** |
| --- | --- | --- | --- | --- | --- | --- | --- | --- | --- | --- | --- | --- |
| Total reads | 49657242 | 46934120 | 46942996 | 48212172 | 49236114 | 48375018 | 48169904 | 49778518 | 49611330 | 48419582 | 46630510 | 48238286 |
| Total mapped reads | 44576539(89.77%) | 42153379(89.81%) | 42230779(89.96%) | 43298605(89.81%) | 44112287(89.59%) | 43480795(89.88%) | 43390267(90.08%) | 44843875(90.09%) | 44702605(90.11%) | 43398624(89.63%) | 41845712(89.74%) | 43213911(89.58%) |
| Multiple mapped | 1257985(2.53%) | 1191540(2.54%) | 1240742(2.64%) | 1256605(2.61%) | 1272113(2.58%) | 1241962(2.57%) | 1229184(2.55%) | 1311902(2.64%) | 1314224(2.65%) | 1281986(2.65%) | 1251968(2.68%) | 1270908(2.63%) |
| Uniquely mapped | 43318554(87.24%) | 40961839(87.28%) | 40990037(87.32%) | 42042000(87.20%) | 42840174(87.01%) | 42238833(87.32%) | 42161083(87.53%) | 43531973(87.45%) | 43388381(87.46%) | 42116638(86.98%) | 40593744(87.05%) | 41943003(86.95%) |
| Read-1 | 21670338(43.64%) | 20495440(43.67%) | 20515373(43.70%) | 21035819(43.63%) | 21431430(43.53%) | 21130049(43.68%) | 21115831(43.84%) | 21777608(43.75%) | 21706351(43.75%) | 21091133(43.56%) | 20336207(43.61%) | 21017598(43.57%) |
| Read-2 | 21648216(43.60%) | 20466399(43.61%) | 20474664(43.62%) | 21006181(43.57%) | 21408744(43.48%) | 21108784(43.64%) | 21045252(43.69%) | 21754365(43.70%) | 21682030(43.70%) | 21025505(43.42%) | 20257537(43.44%) | 20925405(43.38%) |
| Reads map to '+' | 21650603(43.60%) | 20476378(43.63%) | 20491645(43.65%) | 21018100(43.60%) | 21417924(43.50%) | 21118561(43.66%) | 21080665(43.76%) | 21764083(43.72%) | 21693412(43.73%) | 21053559(43.48%) | 20293599(43.52%) | 20970586(43.47%) |
| Reads map to '-' | 21667951(43.64%) | 20485461(43.65%) | 20498392(43.67%) | 21023900(43.61%) | 21422250(43.51%) | 21120272(43.66%) | 21080418(43.76%) | 21767890(43.73%) | 21694969(43.73%) | 21063079(43.50%) | 20300145(43.53%) | 20972417(43.48%) |
| Non-splice reads | 25002146(50.35%) | 23356015(49.76%) | 23306897(49.65%) | 24892533(51.63%) | 25260110(51.30%) | 24731155(51.12%) | 24258865(50.36%) | 25309366(50.84%) | 25167026(50.73%) | 24300821(50.19%) | 23287717(49.94%) | 24126961(50.02%) |
| Splice reads | 18316408(36.89%) | 17605824(37.51%) | 17683140(37.67%) | 17149467(35.57%) | 17580064(35.71%) | 17507678(36.19%) | 17902218(37.16%) | 18222607(36.61%) | 18221355(36.73%) | 17815817(36.79%) | 17306027(37.11%) | 17816042(36.93%) |
| Reads mapped in proper pairs | 41206196(82.98%) | 38892784(82.87%) | 38926602(82.92%) | 39946328(82.86%) | 40723048(82.71%) | 40171214(83.04%) | 40054072(83.15%) | 41443012(83.25%) | 41264262(83.18%) | 40031802(82.68%) | 38514816(82.60%) | 39731516(82.37%) |

**Table S3** List of the core genes set involved in CS response to cold stress.

| **gene_id** | **gene_symbol** | **description** | **up_down** |
| --- | --- | --- | --- |
| BraA09g051320.3C | ARF18 | auxin response factor 18 | Up |
| BraA01g038880.3C | CYS6 | cysteine proteinase inhibitor 6 | Up |
| BraA05g010440.3C | PID | protein kinase PINOID | Up |
| BraA02g010660.3C | ZAT12 | zinc finger protein ZAT12 | Up |
| BraA09g040290.3C | GI | protein GIGANTEA | Up |
| BraA06g039880.3C | PDF2.3 | defensin-like protein 2 | Up |
| BraA06g039840.3C | PP2B1 | F-box protein PP2-B1-like | Up |
| BraA06g040000.3C |  | uncharacterized LOC103827540 | Up |
| BraA09g022970.3C | LPP1 | lipid phosphate phosphatase 1 | Up |
| BraA01g023480.3C | PSK3 | phytosulfokines 3 | Up |
| BraA06g041210.3C | NUDT8 | nudix hydrolase 8 | Up |
| BraA06g041330.3C | PBL19 | probable receptor-like protein kinase At5g47070 | Up |
| BraA06g041730.3C |  | uncharacterized LOC103827715 | Up |
| BraA06g041810.3C | NAC096 | NAC domain-containing protein 86-like | Up |
| BraA06g042180.3C | PYL11 | abscisic acid receptor PYL11 | Up |
| BraA06g042940.3C | MCM6 | DNA replication licensing factor MCM6 | Up |
| BraA06g043450.3C | CRRSP56 | cysteine-rich repeat secretory protein 56 | Up |
| BraA01g023150.3C |  | uncharacterized LOC103827959 | Up |
| BraA06g043750.3C |  | uncharacterized LOC103827973 | Up |
| BraA06g043860.3C |  | uncharacterized LOC103827985 | Up |
| BraA06g043870.3C | AZF3 | zinc finger protein AZF3 | Up |
| BraA06g044080.3C |  | uncharacterized LOC103828008 | Up |
| BraA07g000180.3C |  | uncharacterized LOC103828156 | Up |
| BraA07g000450.3C |  | uncharacterized LOC103828180 | Up |
| BraA07g000490.3C | ELI5 | tyrosine decarboxylase 1-like | Up |
| BraA01g043570.3C | BGLU25 | probable inactive beta-glucosidase 25 | Up |
| BraA07g001810.3C | ADO2 | adagio protein 2-like | Up |
| BraA07g006020.3C |  | thymocyte nuclear protein 1 | Up |
| BraA07g007620.3C | AGPS1 | glucose-1-phosphate adenylyltransferase small subunit, chloroplastic | Up |
| BraA07g007640.3C | DTX6 | protein DETOXIFICATION 6 | Up |
| BraA01g003020.3C | BIG4 | brefeldin A-inhibited guanine nucleotide-exchange protein 4 | Up |
| BraA07g008050.3C | BGLU9 | beta-glucosidase 9-like | Up |
| BraA07g009880.3C | MYB15 | myb-related protein Myb4 | Up |
| BraA07g010350.3C | At1g30760 | flavin-dependent oxidoreductase FOX2-like | Up |
| BraA07g010700.3C | GA2OX2 | gibberellin 2-beta-dioxygenase 2 | Up |
| BraA07g011300.3C |  | uncharacterized LOC103828973 | Up |
| BraA01g003070.3C |  | uncharacterized LOC103828983 | Up |
| BraA07g011440.3C | TBL38 | protein trichome birefringence-like 38 | Up |
| BraA07g011560.3C | SPBC1703.11 | OPA3-like protein | Up |
| BraA07g013060.3C | PDCB4 | carbohydrate-binding X8 domain-containing protein | Up |
| BraA07g015360.3C |  | early nodulin-like protein 1 | Up |
| BraA07g015400.3C |  | uncharacterized LOC103829395 | Up |
| BraA07g015720.3C | 4CLL4 | 4-coumarate--CoA ligase-like 4 | Up |
| BraA07g015740.3C | COR47 | dehydrin ERD10-like | Up |
| BraA07g016510.3C |  | uncharacterized LOC103829506 | Up |
| BraA07g016840.3C | POLA | DNA polymerase alpha catalytic subunit | Up |
| BraA07g017160.3C |  | uncharacterized LOC103829566 | Up |
| BraA07g017800.3C | SPAC17H9.04c | zinc finger Ran-binding domain-containing protein 2 | Up |
| BraA07g018020.3C |  | uncharacterized LOC103829651 | Up |
| BraA07g018130.3C | SEN102 | ervatamin-B | Up |
| BraA07g018560.3C |  | uncharacterized LOC103829711 | Up |
| BraA07g018870.3C | LBD11 | LOB domain-containing protein 11-like | Up |
| BraA07g021860.3C | GATA8 | GATA transcription factor 8-like | Up |
| BraA07g021880.3C | PIP2-5 | probable aquaporin PIP2-5 | Up |
| BraA07g022010.3C | ABCG18 | ABC transporter G family member 18-like | Up |
| BraA07g022210.3C | FER3 | ferritin-3, chloroplastic | Up |
| BraA07g022650.3C | SSL9 | protein STRICTOSIDINE SYNTHASE-LIKE 9-like | Up |
| BraA07g023480.3C | RH52 | DEAD-box ATP-dependent RNA helicase 52 | Up |
| BraA07g023870.3C | PTI13 | PTI1-like tyrosine-protein kinase 3 | Up |
| BraA07g024250.3C |  | uncharacterized LOC103830256 | Up |
| BraA07g024550.3C |  | O-glucosyltransferase rumi homolog | Up |
| BraA07g024690.3C | SLD1 | delta(8)-fatty-acid desaturase 1 | Up |
| BraA07g024820.3C | At3g61750 | cytochrome b561 and DOMON domain-containing protein At3g61750-like | Up |
| BraA07g024830.3C | DRP1B | dynamin-related protein 1B | Up |
| BraA07g025300.3C | GRXC14 | glutaredoxin-C14-like | Up |
| BraA07g025480.3C | At1g79820 | probable plastidic glucose transporter 3 | Up |
| BraA07g025540.3C | RTEL1 | regulator of telomere elongation helicase 1 | Up |
| BraA07g025740.3C | AATP1 | ADP,ATP carrier protein 1, chloroplastic-like | Up |
| BraA07g025840.3C | AVT6E | sodium-coupled neutral amino acid transporter 4-like | Up |
| BraA01g029070.3C | SD129 | G-type lectin S-receptor-like serine/threonine-protein kinase SD1-29 | Up |
| BraA07g027310.3C | CML38 | calcium-binding protein CML38-like | Up |
| BraA02g022580.3C | At1g75040 | pathogenesis-related protein 5-like | Up |
| BraA07g028740.3C | SUS6 | sucrose synthase 6-like | Up |
| BraA07g029480.3C | PER12 | peroxidase 12-like | Up |
| BraA01g029380.3C | YPL109C | uncharacterized aarF domain-containing protein kinase 2 | Up |
| BraA02g019810.3C | ARF2-A | ADP-ribosylation factor 2-B-like | Up |
| BraA07g030120.3C | GSTU11 | glutathione S-transferase U11 | Up |
| BraA07g030700.3C | NPF3.1 | protein NRT1/ PTR FAMILY 3.1-like | Up |
| BraA07g030710.3C | XYL1 | alpha-xylosidase 1-like | Up |
| BraA07g031750.3C | YSL7 | probable metal-nicotianamine transporter YSL7 | Up |
| BraA07g031850.3C | GAD2 | glutamate decarboxylase 2 | Up |
| BraA07g031860.3C | PRXIIB | peroxiredoxin-2B-like | Up |
| BraA07g032120.3C | CAM1 | calmodulin-1 | Up |
| BraA07g032210.3C | PXMT1 | probable S-adenosylmethionine-dependent methyltransferase At5g38100 | Up |
| BraA07g032400.3C | At1g66830 | probable inactive leucine-rich repeat receptor-like protein kinase At1g66830 | Up |
| BraA01g029590.3C | GPT2 | glucose-6-phosphate/phosphate translocator 2, chloroplastic | Up |
| BraA07g033580.3C | GT17 | probable xyloglucan galactosyltransferase GT17 | Up |
| BraA07g033610.3C |  | uncharacterized LOC103831261 | Up |
| BraA07g033680.3C | At4g39670 | ACD11 homolog protein-like | Up |
| BraA07g034640.3C |  | uncharacterized LOC103831516 | Up |
| BraA07g034700.3C | AMY3 | alpha-amylase 3, chloroplastic | Up |
| BraA07g034830.3C | GSTU11 | glutathione S-transferase U11-like | Up |
| BraA07g034850.3C | Os05g0239150 | zinc finger BED domain-containing protein RICESLEEPER 1-like | Up |
| BraA08g018710.3C | LECRK52 | putative L-type lectin-domain containing receptor kinase V.2 | Up |
| BraA07g035390.3C |  | uncharacterized LOC103831599 | Up |
| BraA07g035580.3C | MLP31 | MLP-like protein 31 | Up |
| BraA07g035610.3C | ATHB-X | homeobox-leucine zipper protein ATHB-X | Up |
| BraA07g035930.3C | SUN4 | uncharacterized protein slp1 | Up |
| BraA07g036160.3C | IP5P3 | type IV inositol polyphosphate 5-phosphatase 3 | Up |
| BraA07g036560.3C |  | E3 ubiquitin-protein ligase RNF170 | Up |
| BraA07g036580.3C | BHLH96 | transcription factor bHLH96-like | Up |
| BraA07g036860.3C | TIFY11B | protein TIFY 11B | Up |
| BraA07g037030.3C | ALA5 | probable phospholipid-transporting ATPase 5 | Up |
| BraA07g037810.3C |  | uncharacterized LOC103831856 | Up |
| BraA07g028230.3C |  | uncharacterized LOC103831920 | Up |
| BraA07g038470.3C | MYB96 | myb-related protein 306-like | Up |
| BraA07g038820.3C | SPCC23B6.04c | random slug protein 5 | Up |
| BraA07g038900.3C | At1g75280 | isoflavone reductase homolog P3-like | Up |
| BraA07g040340.3C | At1g21140 | vacuolar iron transporter homolog 2-like | Up |
| BraA09g035550.3C | ERD2A | ER lumen protein-retaining receptor A-like | Up |
| BraA07g041250.3C | TTL3 | inactive TPR repeat-containing thioredoxin TTL3 | Up |
| BraA07g042400.3C | ALDH5F1 | succinate-semialdehyde dehydrogenase, mitochondrial | Up |
| BraA07g042860.3C | At1g80640 | probable receptor-like protein kinase At1g80640 | Up |
| BraA08g002630.3C |  | uncharacterized LOC103832845 | Up |
| BraA08g002860.3C |  | golgin subfamily A member 5-like | Up |
| BraA08g003790.3C | JGB | myosin heavy chain kinase B | Up |
| BraA08g003840.3C | CCS1 | cytochrome c biogenesis protein CCS1, chloroplastic | Up |
| BraA08g004210.3C |  | non-specific lipid-transfer protein 2-like | Up |
| BraA01g003390.3C | ADF9 | actin-depolymerizing factor 9 | Up |
| BraA08g005640.3C | TRX5 | thioredoxin H5 | Up |
| BraA08g005680.3C | MCM2 | DNA replication licensing factor MCM2-like | Up |
| BraA08g005780.3C | At1g44800 | WAT1-related protein At1g44800 | Up |
| BraA10g022520.3C |  | uncharacterized LOC103833177 | Up |
| BraA01g003410.3C | NFD4 | protein NUCLEAR FUSION DEFECTIVE 4 | Up |
| BraA08g010590.3C | At3g50710 | putative FBD-associated F-box protein At3g50710 | Up |
| BraA08g010890.3C | PBD1 | proteasome subunit beta type-2-A-like | Up |
| BraA08g011620.3C |  | proline-rich antigen homolog | Up |
| BraA08g007890.3C | ANN1 | annexin D1 | Up |
| BraA01g030520.3C | DTX19 | protein DETOXIFICATION 19-like | Up |
| BraA01g003440.3C | XDH1 | xanthine dehydrogenase 1 | Up |
| BraA08g009620.3C | GT14 | probable xyloglucan galactosyltransferase GT14 | Up |
| BraA09g026940.3C | GPX6 | probable phospholipid hydroperoxide glutathione peroxidase 6, mitochondrial | Up |
| BraA09g027580.3C | LACS5 | long chain acyl-CoA synthetase 5 | Up |
| BraA08g012870.3C |  | uncharacterized LOC103834054 | Up |
| BraA08g013370.3C | CCD4 | probable carotenoid cleavage dioxygenase 4, chloroplastic | Up |
|  |  | glycine-rich protein A3-like | Up |
| BraA08g013500.3C |  | uncharacterized protein DDB_G0290587 | Up |
| BraA01g031210.3C |  | uncharacterized LOC103834190 | Up |
| BraA01g031260.3C | ACA13 | putative calcium-transporting ATPase 13, plasma membrane-type | Up |
| BraA01g031350.3C | ELIP1 | early light-induced protein 1, chloroplastic | Up |
| BraA03g059240.3C | ASMT | (R,S)-reticuline 7-O-methyltransferase-like | Up |
| BraA08g016160.3C | UGT73B3 | UDP-glucosyl transferase 73B2-like | Up |
| BraA08g016300.3C |  | uncharacterized LOC103834447 | Up |
| BraA08g016550.3C |  | uncharacterized LOC103834473 | Up |
| BraA01g031650.3C |  | uncharacterized LOC103834564 | Up |
| BraA08g017890.3C | ASHR3 | histone-lysine N-methyltransferase ASHR3 | Up |
| BraA08g018490.3C | SPBC725.05c | venom phosphodiesterase 2-like | Up |
| BraA08g019610.3C | RBP45C | polyadenylate-binding protein RBP45C-like | Up |
| BraA08g019640.3C |  | uncharacterized LOC103834827 | Up |
| BraA08g020060.3C | DREB1A | dehydration-responsive element-binding protein 1B-like | Up |
| BraA08g020480.3C | VEP1 | 3-oxo-Delta(4,5)-steroid 5-beta-reductase | Up |
| BraA08g020550.3C | ACO1 | aconitate hydratase 1-like | Up |
| BraA01g001490.3C | CYP81F4 | cytochrome P450 81F4-like | Up |
| BraA08g021830.3C | CAD8 | cinnamyl alcohol dehydrogenase 8 | Up |
| BraA01g032130.3C | UGT84A2 | UDP-glycosyltransferase 84A2 | Up |
| BraA08g022270.3C | PLAT1 | PLAT domain-containing protein 2 | Up |
| BraA08g022450.3C | TKI1 | uncharacterized LOC103835124 | Up |
| BraA01g000350.3C | GRP10 | glycine-rich RNA-binding protein 10-like | Up |
| BraA08g022650.3C | RD19A | cysteine protease RD19A-like | Up |
| BraA08g022800.3C | Os04g0620700 | nucleolin 2-like | Up |
| BraA08g022960.3C | HIPP26 | heavy metal-associated isoprenylated plant protein 26 | Up |
| BraA08g023120.3C | CBSDUF2 | DUF21 domain-containing protein At4g14230 | Up |
| BraA01g032280.3C | SRG1 | protein SRG1-like | Up |
| BraA08g024790.3C | CYCT1-3 | cyclin-T1-3 | Up |
| BraA07g013000.3C | TTM2 | uridine-cytidine kinase C | Up |
| BraA08g025960.3C | ORC6 | origin of replication complex subunit 6 | Up |
| BraA08g026200.3C | At1g27200 | glycosyltransferase family 92 protein At1g27200-like | Up |
| BraA09g040490.3C | NPF5.16 | protein NRT1/ PTR FAMILY 5.15 | Up |
| BraA08g026970.3C | TGA3 | transcription factor TGA3 | Up |
| BraA08g027170.3C | PDIL1-1 | protein disulfide isomerase-like 1-1 | Up |
| BraA08g027710.3C | HMGB2 | high mobility group B protein 2 | Up |
| BraA08g028540.3C | SEC31A | protein transport protein SEC31 homolog A | Up |
| BraA08g028630.3C | IBS1 | probable serine/threonine-protein kinase At1g54610 | Up |
| BraA08g028770.3C | LIP2 | lipase 1 | Up |
| BraA08g028780.3C | CSE | monoglyceride lipase | Up |
| BraA01g003660.3C | SQS2 | squalene synthase 1 | Up |
| BraA06g011560.3C | AATP2 | ADP,ATP carrier protein 2, chloroplastic-like | Up |
| BraA08g030030.3C | PMEI11 | 21 kDa protein-like | Up |
| BraA08g030410.3C | NRP1 | nodulin-related protein 1-like | Up |
| BraA06g008930.3C |  | uncharacterized LOC103836137 | Up |
| BraA08g031200.3C | EARLI1 | 14 kDa proline-rich protein DC2.15 | Up |
| BraA08g032720.3C |  | uncharacterized LOC103836340 | Up |
| BraA10g027510.3C |  | histone H3.2 | Up |
| BraA08g032930.3C | ERD6 | sugar transporter ERD6-like | Up |
| BraA08g033370.3C | APX1 | L-ascorbate peroxidase 1, cytosolic-like | Up |
| BraA09g063090.3C | hh4 | histone H4-like | Up |
| BraA08g033900.3C | At1g06470 | probable sugar phosphate/phosphate translocator At1g06470 | Up |
| BraA08g034280.3C | IP5P13 | type I inositol polyphosphate 5-phosphatase 13 | Up |
| BraA08g034430.3C | rhp16 | DNA repair protein RAD16-like | Up |
| BraA08g034690.3C |  | uncharacterized LOC103836558 | Up |
| BraA08g035430.3C | BG | basic 7S globulin-like | Up |
| BraA08g035560.3C | RALF1 | protein RALF-like 1 | Up |
| BraA08g035650.3C | ABCB11 | ABC transporter B family member 11 | Up |
| BraA08g035760.3C | LEA14 | probable desiccation-related protein LEA14 | Up |
| BraA09g001840.3C | PME41 | probable pectinesterase/pectinesterase inhibitor 41 | Up |
| BraA09g002350.3C | Cht10 | chitinase 10 | Up |
| BraA09g002470.3C | NAC69 | NAC domain-containing protein 69-like | Up |
| BraA09g000540.3C | BAM2 | beta-amylase 2, chloroplastic | Up |
| BraA09g003200.3C | At3g27400 | putative pectate lyase 11 | Up |
| BraA09g003260.3C | cdt2 | denticleless protein homolog | Up |
| BraA09g003330.3C | RPL12A | 50S ribosomal protein L12-3, chloroplastic-like | Up |
| BraA09g006120.3C | APRR5 | two-component response regulator-like APRR5 | Up |
| BraA09g005570.3C | STP13 | sugar transport protein 13 | Up |
| BraA09g005550.3C | GWD3 | phosphoglucan, water dikinase, chloroplastic | Up |
| BraA09g004520.3C | BZIP1 | basic leucine zipper 1-like | Up |
| BraA09g004550.3C | CP1 | calmodulin | Up |
| BraA09g006720.3C | DOF5.4 | dof zinc finger protein DOF5.4-like | Up |
| BraA09g006930.3C | APRR1 | two-component response regulator-like APRR1 | Up |
| BraA09g006940.3C | NEN2 | protein NEN2 | Up |
| BraA09g007270.3C |  | 21 kDa protein-like | Up |
| BraA09g007540.3C |  | uncharacterized LOC103837437 | Up |
| BraA09g007680.3C | RH30 | DEAD-box ATP-dependent RNA helicase 30 | Up |
| BraA09g007950.3C |  | pyruvate kinase, cytosolic isozyme-like | Up |
| BraA09g008350.3C | PME64 | probable pectinesterase/pectinesterase inhibitor 64 | Up |
| BraA09g008390.3C | At5g64700 | WAT1-related protein At5g64700 | Up |
| BraA06g028200.3C | ABR1 | ethylene-responsive transcription factor ABR1-like | Up |
| BraA10g027510.3C |  | histone H3.2 | Up |
| BraA09g009340.3C | Os10g0391300 | zinc finger CCCH domain-containing protein 62 | Up |
| BraA09g009020.3C | SBT1.7 | subtilisin-like protease SBT1.7 | Up |
| BraA09g054850.3C | AGP2 | classical arabinogalactan protein 2-like | Up |
| BraA09g055060.3C | PLAT2 | PLAT domain-containing protein 2-like | Up |
| BraA09g011660.3C | BHLH146 | uncharacterized protein At4g30180 | Up |
| BraA01g034310.3C | At4g11060 | single-stranded DNA-binding protein, mitochondrial-like | Up |
| BraA09g012210.3C | XI-G | myosin-13-like | Up |
| BraA01g034450.3C | CFM3 | CRM-domain containing factor CFM3, chloroplastic/mitochondrial | Up |
| BraA09g015130.3C | BHLH2 | transcription factor EGL1 | Up |
| BraA09g013650.3C | PGD1 | 6-phosphogluconate dehydrogenase, decarboxylating 1, chloroplastic | Up |
| BraA09g012960.3C | RFC2 | replication factor C subunit 2 | Up |
| BraA01g003850.3C | ERF109 | ethylene-responsive transcription factor ERF109 | Up |
| BraA03g065120.3C | SD129 | G-type lectin S-receptor-like serine/threonine-protein kinase SD1-29 | Up |
| BraA09g018130.3C | SOT18 | cytosolic sulfotransferase 18-like | Up |
| BraA09g018290.3C | CYP96A15 | alkane hydroxylase MAH1-like | Up |
| BraA09g019200.3C | CML37 | calcium-binding protein CML37 | Up |
| BraA09g028520.3C | MES12 | putative methylesterase 12, chloroplastic | Up |
| BraA09g036640.3C |  | uncharacterized LOC103838992 | Up |
| BraA09g036800.3C | At1g27930 | probable methyltransferase At1g27930 | Up |
| BraA09g036820.3C | PUB45 | U-box domain-containing protein 45-like | Up |
| BraA05g029950.3C | AGD15 | probable ADP-ribosylation factor GTPase-activating protein AGD15 | Up |
| BraA09g020290.3C | ABCI21 | ABC transporter I family member 21 | Up |
| BraA09g019530.3C |  | uncharacterized LOC103839269 | Up |
| BraA09g019290.3C |  | uncharacterized LOC103839296 | Up |
| BraA01g035280.3C | MSAMS2 | S-adenosylmethionine synthase 2 | Up |
| BraA09g023260.3C | PMEI10 | 21 kDa protein-like | Up |
| BraA09g023250.3C | HMGA | HMG-Y-related protein A-like | Up |
| BraA09g022670.3C | HAT2 | homeobox-leucine zipper protein HAT2-like | Up |
| BraA09g022420.3C | JMJ706 | lysine-specific demethylase JMJ706-like | Up |
| BraA09g022280.3C |  | uncharacterized LOC103839583 | Up |
| BraA01g035530.3C |  | phylloplanin-like | Up |
| BraA01g035500.3C |  | phylloplanin-like | Up |
| BraA09g021780.3C | FLS2 | LRR receptor-like serine/threonine-protein kinase FLS2 | Up |
| BraA01g035460.3C | C/VIF1 | cell wall / vacuolar inhibitor of fructosidase 1-like | Up |
| BraA09g024210.3C | SOT12 | cytosolic sulfotransferase 12-like | Up |
| BraA09g021780.3C | FLS2 | LRR receptor-like serine/threonine-protein kinase FLS2 | Up |
| BraA09g025040.3C | ENT3 | equilibrative nucleotide transporter 3 | Up |
| BraA09g025830.3C | CRK36 | cysteine-rich receptor-like protein kinase 36 | Up |
| BraA09g025810.3C | CRK36 | cysteine-rich receptor-like protein kinase 36 | Up |
| BraA09g026300.3C | At4g12490 | lipid transfer protein EARLI 1 | Up |
| BraA09g026310.3C |  | uncharacterized LOC103839888 | Up |
| BraA09g026600.3C |  | TVP38/TMEM64 family membrane protein slr0305 | Up |
| BraA05g030730.3C | NSP2 | nitrile-specifier protein 2-like | Up |
| BraA09g032260.3C | SBT3.5 | subtilisin-like protease SBT3.5 | Up |
| BraA09g032320.3C | At1g32860 | glucan endo-1,3-beta-glucosidase 11 | Up |
| BraA09g033530.3C | HCBT1 | anthranilate N-benzoyltransferase protein 1 | Up |
| BraA09g033930.3C | OSB4 | protein OSB4, chloroplastic | Up |
| BraA09g034270.3C | At1g30730 | reticuline oxidase-like protein | Up |
| BraA01g036450.3C |  | uncharacterized LOC103840517 | Up |
| BraA01g036560.3C | UVR3 | (6-4)DNA photolyase | Up |
| BraA09g038590.3C |  | uncharacterized LOC103840646 | Up |
| BraA09g038710.3C | ORC6 | origin of replication complex subunit 6-like | Up |
| BraA09g038920.3C | ATHB-54 | homeobox-leucine zipper protein ATHB-54 | Up |
| BraA09g038970.3C | GALT4 | hydroxyproline O-galactosyltransferase GALT4-like | Up |
| BraA01g004070.3C | PGDH1 | D-3-phosphoglycerate dehydrogenase 1, chloroplastic | Up |
| BraA09g039930.3C | DYN2 | dynein light chain 1, cytoplasmic-like | Up |
| BraA09g040000.3C | CAR8 | protein C2-DOMAIN ABA-RELATED 8 | Up |
| BraA09g040080.3C | LPR1 | multicopper oxidase LPR1 | Up |
| BraA09g041640.3C | DOF3.4 | dof zinc finger protein DOF3.4-like | Up |
| BraA09g042200.3C | HSR4 | protein HYPER-SENSITIVITY-RELATED 4 | Up |
| BraA09g042420.3C | FHT | naringenin,2-oxoglutarate 3-dioxygenase | Up |
| BraA09g042930.3C |  | macrophage migration inhibitory factor homolog | Up |
| BraA09g043250.3C |  | uncharacterized LOC103841142 | Up |
| BraA09g043390.3C | DSP4 | phosphoglucan phosphatase DSP4, chloroplastic | Up |
| BraA09g043520.3C |  | uncharacterized LOC103841167 | Up |
| BraA09g043810.3C | BIC1 | uncharacterized LOC103841203 | Up |
| BraA09g043900.3C | PAP22 | purple acid phosphatase 22 | Up |
| BraA09g044420.3C | CP29A | 29 kDa ribonucleoprotein, chloroplastic-like | Up |
| BraA09g044780.3C | FES1 | hsp70-binding protein 1 | Up |
| BraA03g019670.3C | LECRK42 | L-type lectin-domain containing receptor kinase IV.2 | Up |
| BraA09g044840.3C | AKR4C11 | aldo-keto reductase family 4 member C11 | Up |
| BraA09g045350.3C | EP3 | endochitinase EP3 | Up |
| BraA09g046060.3C | CHI | chalcone--flavonone isomerase | Up |
| BraA09g046410.3C | UVR8 | ultraviolet-B receptor UVR8 | Up |
| BraA09g046430.3C | P5CSB | delta-1-pyrroline-5-carboxylate synthase B | Up |
| BraA09g046670.3C | PLC7 | phosphoinositide phospholipase C 7 | Up |
| BraA09g046800.3C | HTH | protein HOTHEAD-like | Up |
| BraA09g046810.3C | HTH | protein HOTHEAD-like | Up |
| BraA09g046800.3C | HTH | protein HOTHEAD-like | Up |
| BraA09g047040.3C | TRM1 | probable tRNA (guanine(26)-N(2))-dimethyltransferase | Up |
| BraA01g004150.3C | UGT73B1 | UDP-glycosyltransferase 73B1 | Up |
|  |  | uncharacterized LOC103841621 | Up |
| BraA04g029750.3C | ZAR1 | receptor protein kinase-like protein ZAR1 | Up |
| BraA09g048450.3C | GIG1 | protein GIGAS CELL1 | Up |
| BraA09g048820.3C | RH52 | DEAD-box ATP-dependent RNA helicase 52-like | Up |
| BraA01g036960.3C | MORF8 | multiple organellar RNA editing factor 8, chloroplastic/mitochondrial-like | Up |
| BraA09g049380.3C | RLP6 | receptor-like protein 12 | Up |
| BraA09g049660.3C | mdm28 | LETM1 and EF-hand domain-containing protein 1, mitochondrial | Up |
| BraA09g049950.3C | BGLU27 | beta-glucosidase 27 | Up |
| BraA09g050370.3C |  | uncharacterized LOC103841906 | Up |
| BraA09g050410.3C | At3g60660 | spindle and kinetochore-associated protein 1 homolog | Up |
| BraA09g050760.3C | BAP1 | BON1-associated protein 1 | Up |
| BraA08g016200.3C | CBSX2 | CBS domain-containing protein CBSX2, chloroplastic-like | Up |
| BraA04g000610.3C | BGLU10 | beta-glucosidase 10-like | Up |
| BraA09g052110.3C | UXS2 | UDP-glucuronic acid decarboxylase 2-like | Up |
| BraA09g052960.3C | MOT1 | molybdate transporter 1 | Up |
| BraA09g052980.3C | At2g25620 | probable protein phosphatase 2C 22 | Up |
| BraA09g047900.3C | BG2 | glucan endo-1,3-beta-glucosidase, acidic isoform-like | Up |
| BraA09g054400.3C |  | uncharacterized LOC103842441 | Up |
| BraA09g054500.3C | VQ29 | VQ motif-containing protein 29 | Up |
| BraA09g055100.3C |  | uncharacterized LOC103842478 | Up |
| BraA09g055350.3C |  | glycine-rich RNA-binding protein GRP1A | Up |
| BraA09g055360.3C | RL2 | protein RADIALIS-like 2 | Up |
| BraA09g055500.3C |  | uncharacterized LOC103842522 | Up |
| BraA09g055610.3C | BBX19 | B-box zinc finger protein 18 | Up |
| BraA09g056720.3C | At1g75040 | pathogenesis-related protein 5-like | Up |
| BraA09g056830.3C | TIFY10A | protein TIFY 10A-like | Up |
| BraA06g012720.3C |  | uncharacterized LOC103842825 | Up |
| BraA01g004240.3C |  | uncharacterized LOC103842877 | Up |
| BraA09g058530.3C | CAP10A | uncharacterized LOC103842880 | Up |
| BraA09g059120.3C | At1g14780 | MACPF domain-containing protein At1g14780-like | Up |
| BraA09g059250.3C | XRI1 | uncharacterized LOC103842955 | Up |
| BraA06g008050.3C | PYM | partner of Y14 and mago-like | Up |
| BraA09g060530.3C | At1g13200 | putative F-box protein At1g12855 | Up |
| BraA09g061530.3C | GWD1 | alpha-glucan water dikinase 1, chloroplastic | Up |
| BraA09g061760.3C | At1g48100 | polygalacturonase At1g48100-like | Up |
| BraA09g061790.3C | BHLH167 | uncharacterized LOC103843290 | Up |
| BraA09g062080.3C | AAP1 | amino acid permease 8-like | Up |
| BraA09g062120.3C | DOGL4 | TGACG-sequence-specific DNA-binding protein TGA-2.1-like | Up |
| BraA09g062410.3C | GOLS3 | galactinol synthase 3 | Up |
| BraA09g063050.3C | APX1 | L-ascorbate peroxidase 1, cytosolic | Up |
| BraA09g063060.3C | MPK13 | mitogen-activated protein kinase 13 | Up |
| BraA09g063470.3C | NUCL1 | nucleolin 1-like | Up |
| BraA09g063490.3C | UGT71C4 | UDP-glycosyltransferase 71C4 | Up |
| BraA09g063510.3C | rps18 | uncharacterized LOC103843475 | Up |
| BraA09g061370.3C | At1g11330 | G-type lectin S-receptor-like serine/threonine-protein kinase At1g11330 | Up |
| BraA09g064310.3C |  | protein FAM136A | Up |
| BraA09g065100.3C | JASON | uncharacterized LOC103843673 | Up |
| BraA09g065680.3C | PLIP2 | uncharacterized LOC103843744 | Up |
| BraA09g066150.3C | At1g01500 | uncharacterized protein At1g01500-like | Up |
| BraA10g006370.3C | At1g47710 | serpin-ZX | Up |
| BraA10g006310.3C | At1g47840 | hexokinase-like 1 protein | Up |
| BraA10g005200.3C | At1g06470 | probable sugar phosphate/phosphate translocator At1g06470 | Up |
| BraA10g005190.3C | HSP17.7 | uncharacterized LOC103844038 | Up |
| BraA10g004890.3C | MYB13 | myb-related protein Myb4 | Up |
| BraA10g003690.3C | MAPKKK18 | mitogen-activated protein kinase kinase kinase 2 | Up |
| BraA01g043700.3C |  | uncharacterized LOC103844182 | Up |
| BraA10g003570.3C | At1g04990 | zinc finger CCCH domain-containing protein 3 | Up |
| BraA10g003250.3C | ctf18 | chromosome transmission fidelity protein 18 homolog | Up |
| BraA10g003120.3C | At1g04570 | probable folate-biopterin transporter 8, chloroplastic | Up |
| BraA10g002620.3C | SBT1.2 | subtilisin-like protease SBT1.2 | Up |
| BraA06g022370.3C | DTX9 | protein DETOXIFICATION 9 | Up |
| BraA10g001500.3C | BAH1 | E3 ubiquitin-protein ligase BAH1 | Up |
| BraA10g001470.3C | RPL22C | 60S ribosomal protein L22-3-like | Up |
| BraA10g001460.3C | LEA2 | protein SENESCENCE-ASSOCIATED GENE 21, mitochondrial | Up |
| BraA10g001450.3C | At5g01610 | uncharacterized LOC103844560 | Up |
| BraA10g001260.3C | At1g48100 | polygalacturonase At1g48100 | Up |
| BraA10g001050.3C | MAN1 | mannan endo-1,4-beta-mannosidase 1 | Up |
| BraA10g001040.3C | TOC33 | translocase of chloroplast 33, chloroplastic | Up |
| BraA10g000640.3C | APF2 | aspartyl protease family protein 2 | Up |
| BraA09g066150.3C | At1g01500 | uncharacterized protein At1g01500 | Up |
| BraA10g000370.3C | SPPL4 | signal peptide peptidase-like 4 | Up |
| BraA10g000170.3C | SBTI1.1 | subtilisin-like protease SBT1.1 | Up |
| BraA10g011230.3C | MAKR6 | probable membrane-associated kinase regulator 6 | Up |
| BraA03g014080.3C | OMT1 | flavone 3'-O-methyltransferase 1-like | Up |
| BraA10g012560.3C | MIP1B | putative zinc finger protein CONSTANS-LIKE 11 | Up |
| BraA10g012830.3C | THI1 | thiamine thiazole synthase, chloroplastic | Up |
| BraA10g013180.3C | At5g56590 | glucan endo-1,3-beta-glucosidase 13 | Up |
| BraA10g014240.3C | At4g26390 | probable pyruvate kinase, cytosolic isozyme | Up |
| BraA10g014950.3C | ABI2 | protein phosphatase 2C 77 | Up |
| BraA10g016480.3C | PMR5 | protein PMR5 | Up |
| BraA10g017140.3C | COPT1 | copper transporter 1-like | Up |
| BraA10g017200.3C |  | uncharacterized LOC103845360 | Up |
| BraA10g017830.3C | APRR3 | two-component response regulator-like APRR3 | Up |
| BraA10g018700.3C | At5g22880 | histone H2B.10 | Up |
| BraA10g019380.3C | YLMG2 | ylmG homolog protein 2, chloroplastic | Up |
| BraA10g020020.3C | GLP1 | germin-like protein 1 | Up |
| BraA10g020140.3C | EFR | LRR receptor-like serine/threonine-protein kinase EFR | Up |
| BraA10g020350.3C | BCB | umecyanin | Up |
| BraA10g020890.3C | CCR1 | cinnamoyl-CoA reductase 1 | Up |
| BraA10g021160.3C | LUL4 | probable E3 ubiquitin-protein ligase LUL3 | Up |
| BraA10g021340.3C | SPBC1683.06c | uncharacterized LOC103845800 | Up |
| BraA10g021580.3C | At5g18550 | zinc finger CCCH domain-containing protein 58-like | Up |
| BraA10g021680.3C |  | uncharacterized LOC103845833 | Up |
| BraA10g021800.3C | SIP1-2 | probable aquaporin SIP1-2 | Up |
| BraA07g003080.3C | ERD7 | senescence/dehydration-associated protein At3g51250-like | Up |
| BraA07g003970.3C | LYM2 | lysM domain-containing GPI-anchored protein 2 | Up |
| BraA01g039410.3C | At3g11740 | protein LURP-one-related 10 | Up |
| BraA07g004890.3C | COR413PM1 | cold-regulated 413 plasma membrane protein 1 | Up |
| BraA10g016090.3C | TIL | temperature-induced lipocalin-1-like | Up |
| BraA10g022970.3C | OPR1 | 12-oxophytodienoate reductase 1-like | Up |
| BraA10g023640.3C | KIN2 | stress-induced protein KIN2-like | Up |
| BraA10g024460.3C | SNL6 | cinnamoyl-CoA reductase-like SNL6 | Up |
| BraA10g024810.3C | LIP2 | triacylglycerol lipase 2 | Up |
| BraA10g024990.3C | CHS1 | chalcone synthase 1 | Up |
| BraA10g025320.3C | LKHA4 | leukotriene A-4 hydrolase homolog | Up |
| BraA10g026160.3C | CYS1 | cysteine proteinase inhibitor 1 | Up |
| BraA10g026270.3C | PUB51 | U-box domain-containing protein 51-like | Up |
| BraA10g026970.3C | APT5 | adenine phosphoribosyltransferase 5-like | Up |
| BraA10g027290.3C | FLP2 | flowering-promoting factor 1-like protein 2 | Up |
| BraA10g027340.3C | BHLH61 | transcription factor bHLH61 | Up |
| BraA10g027380.3C | AIL6 | AP2-like ethylene-responsive transcription factor AIL6 | Up |
| BraA10g028350.3C | PYL5 | abscisic acid receptor PYL5 | Up |
| BraA10g028920.3C | At5g06250 | B3 domain-containing protein At5g06250 | Up |
| BraA10g029400.3C | HAT14 | homeobox-leucine zipper protein HAT14 | Up |
| BraA10g029420.3C | PER54 | peroxidase A2 | Up |
| BraA03g002690.3C | LEA46 | 18 kDa seed maturation protein | Up |
| BraA10g029550.3C | PGIP1 | polygalacturonase inhibitor 1-like | Up |
| BraA10g029560.3C | PGIP1 | polygalacturonase inhibitor 1-like | Up |
| BraA10g029560.3C | PGIP1 | polygalacturonase inhibitor 1-like | Up |
| BraA01g039730.3C | CPRD49 | GDSL esterase/lipase CPRD49-like | Up |
| BraA10g029900.3C | BON2 | protein BONZAI 2 | Up |
| BraA10g030180.3C | AED3 | aspartyl protease AED3 | Up |
| BraA02g002210.3C | SOT15 | cytosolic sulfotransferase 15-like | Up |
| BraA10g030360.3C | CYP75B1 | flavonoid 3'-monooxygenase | Up |
| BraA10g030890.3C |  | pyruvate kinase, cytosolic isozyme | Up |
| BraA10g031980.3C | ZAT6 | zinc finger protein ZAT6 | Up |
| BraA10g032090.3C |  | OTU domain-containing protein 5-A | Up |
| BraA10g032960.3C | TOP6A | DNA topoisomerase 6 subunit A | Up |
| BraA10g033340.3C | ABCI20 | ABC transporter I family member 20 | Up |
| BraA10g033570.3C |  | uncharacterized LOC103847486 | Up |
| BraA01g026270.3C | ATJ6 | dnaJ homolog subfamily B member 8 | Up |
| BraA01g040420.3C | CML50 | probable calcium-binding protein CML49 | Up |
| BraA10g007880.3C | At1g43910 | AAA-ATPase At1g43910 | Up |
| BraA02g034850.3C | MLP328 | MLP-like protein 328 | Up |
| BraA09g035960.3C | TBL38 | protein trichome birefringence-like 38 | Up |
| BraA10g007070.3C |  | glycine-rich cell wall structural protein | Up |
| BraA02g030430.3C |  | uncharacterized LOC103848432 | Up |
| BraA05g039480.3C | RCI2A | hydrophobic protein RCI2A | Up |
| BraA05g039500.3C | AGL80 | agamous-like MADS-box protein AGL80 | Up |
| BraA01g001510.3C | GH3.3 | indole-3-acetic acid-amido synthetase GH3.2 | Up |
| BraA01g041130.3C | PLC2 | phosphoinositide phospholipase C 2 | Up |
| BraA01g041190.3C | PIP5K6 | phosphatidylinositol 4-phosphate 5-kinase 6 | Up |
| BraA05g042370.3C | GAPC | glyceraldehyde-3-phosphate dehydrogenase, cytosolic | Up |
| BraAnng005520.3C | WRKY45 | probable WRKY transcription factor 45 | Up |
| BraA01g042700.3C | RLP31 | receptor like protein 30 | Up |
| BraA01g042900.3C |  | uncharacterized LOC103849150 | Up |
| BraA04g009650.3C | At5g35735 | cytochrome b561 and DOMON domain-containing protein At5g35735 | Up |
| BraA07g019200.3C | OEP161 | outer envelope pore protein 16-1, chloroplastic | Up |
| BraA01g041630.3C | PAP15 | purple acid phosphatase 15-like | Up |
| BraA01g041690.3C | NSN1 | guanine nucleotide-binding protein-like NSN1 | Up |
| BraA06g030110.3C | ERD7 | senescence/dehydration-associated protein At3g51250-like | Up |
| BraA05g034230.3C |  | uncharacterized LOC103849716 | Up |
| BraA01g038310.3C | SPO11-1 | meiotic recombination protein SPO11-1 | Up |
| BraA01g038200.3C | ATJ11 | chaperone protein dnaJ 11, chloroplastic-like | Up |
| BraA01g042550.3C | At3g05730 | defensin-like protein 205 | Up |
| BraA01g042280.3C | RCI2B | hydrophobic protein RCI2B | Up |
| BraA01g042360.3C | LON4 | lon protease homolog 4, chloroplastic/mitochondrial-like | Up |
| BraA01g004860.3C |  | rho GTPase-activating protein 7 | Up |
| BraA01g043120.3C |  | uncharacterized LOC103850049 | Up |
| BraA07g020090.3C | HOX16 | homeobox-leucine zipper protein HAT5-like | Up |
| BraA07g020600.3C | At5g41680 | probably inactive receptor-like protein kinase At5g41680 | Up |
| BraA07g020690.3C |  | uncharacterized LOC103850235 | Up |
| BraA07g020860.3C | HIPP30 | heavy metal-associated isoprenylated plant protein 21 | Up |
| BraA01g005140.3C | SPAC19A8.14 | peptidyl-tRNA hydrolase 2, mitochondrial | Up |
| BraA02g000240.3C | GT-3A | trihelix transcription factor GT-3a-like | Up |
| BraA02g000630.3C | APRR7 | two-component response regulator-like APRR7 | Up |
| BraA02g000690.3C | TIM14-3 | mitochondrial import inner membrane translocase subunit TIM14-3 | Up |
| BraA02g000820.3C | IDH5 | isocitrate dehydrogenase [NAD] catalytic subunit 5, mitochondrial-like | Up |
| BraA02g001010.3C | At5g03610 | GDSL esterase/lipase At5g03610-like | Up |
| BraA02g001460.3C | DIVARICATA | transcription factor DIVARICATA-like | Up |
| BraA02g001840.3C | WOX7 | WUSCHEL-related homeobox 7-like | Up |
| BraA02g001870.3C | FPGS1 | folylpolyglutamate synthase | Up |
| BraA02g002210.3C | SOT15 | cytosolic sulfotransferase 15-like | Up |
| BraA02g002350.3C | GDH2 | glutamate dehydrogenase 2-like | Up |
| BraA02g002640.3C |  | uncharacterized LOC103850688 | Up |
| BraA02g002710.3C | AGAL1 | alpha-galactosidase 1 | Up |
| BraA02g002870.3C | NAC081 | protein ATAF2-like | Up |
| BraA02g004220.3C | LOG8 | cytokinin riboside 5'-monophosphate phosphoribohydrolase LOG8 | Up |
| BraA02g004290.3C | PUB34 | U-box domain-containing protein 34 | Up |
| BraA02g004640.3C | ABAP1 | ARMADILLO BTB ARABIDOPSIS PROTEIN 1-like | Up |
| BraA02g004800.3C | ERF113 | ethylene-responsive transcription factor ERF113-like | Up |
| BraA02g005190.3C | CHS3 | chalcone synthase 3-like | Up |
| BraA02g005910.3C |  | uncharacterized LOC103850974 | Up |
| BraA02g005950.3C | AVT1J | vacuolar amino acid transporter 1 | Up |
| BraA01g005660.3C | BT1 | adenine nucleotide transporter BT1, chloroplastic/mitochondrial | Up |
| BraA02g006290.3C | KIN2 | stress-induced protein KIN2 | Up |
| BraA02g006530.3C |  | uncharacterized LOC103851050 | Up |
| BraA01g005710.3C |  | uncharacterized LOC103851057 | Up |
| BraA02g006880.3C | GSTF12 | glutathione S-transferase F12 | Up |
| BraA02g007200.3C | At5g17760 | AAA-ATPase At5g17760-like | Up |
| BraA02g007270.3C | CCX2 | cation/calcium exchanger 2-like | Up |
| BraA02g007720.3C | At5g18550 | zinc finger CCCH domain-containing protein 58-like | Up |
| BraA02g007270.3C | CCX2 | cation/calcium exchanger 2-like | Up |
| BraA02g008230.3C | ACL5 | thermospermine synthase ACAULIS5 | Up |
| BraA03g009450.3C |  | uncharacterized LOC103851327 | Up |
| BraA01g005980.3C | At1g80640 | probable receptor-like protein kinase At1g80640 | Up |
| BraA10g019290.3C |  | ELL-associated factor 1-like | Up |
| BraA01g006160.3C | WIN2 | probable protein phosphatase 2C 59 | Up |
| BraA02g011050.3C |  | uncharacterized LOC103851582 | Up |
| BraA02g012500.3C | ACA8 | calcium-transporting ATPase 8, plasma membrane-type | Up |
| BraA02g013450.3C | At5g56590 | glucan endo-1,3-beta-glucosidase 13-like | Up |
| BraA02g013680.3C | THI1 | thiamine thiazole synthase, chloroplastic-like | Up |
| BraA02g013890.3C | MIP1B | nucleolin-like | Up |
| BraA01g006680.3C | At4g31140 | glucan endo-1,3-beta-glucosidase 5 | Up |
| BraA02g015440.3C | LTI65 | low-temperature-induced 65 kDa protein-like | Up |
| BraA02g015410.3C |  | uncharacterized LOC103852106 | Up |
| BraA02g015450.3C | LTI65 | low-temperature-induced 65 kDa protein-like | Up |
| BraA01g006750.3C | CBP60F | calmodulin-binding protein 60 F | Up |
| BraA02g017770.3C | pol12 | DNA polymerase alpha subunit B | Up |
| BraA02g017880.3C | SUFE2 | sufE-like protein 2, chloroplastic | Up |
| BraA02g017890.3C | At1g67820 | probable protein phosphatase 2C 14 | Up |
| BraA02g018440.3C |  | uncharacterized LOC103852488 | Up |
| BraA01g007180.3C | At4g30650 | UPF0057 membrane protein At4g30650 | Up |
| BraA02g019460.3C | NPF2.13 | protein NRT1/ PTR FAMILY 2.13 | Up |
| BraA02g019780.3C | BLUS1 | serine/threonine-protein kinase BLUS1-like | Up |
| BraA02g020110.3C | MLP28 | MLP-like protein 31 | Up |
| BraA02g020150.3C | ATHB-X | homeobox-leucine zipper protein ATHB-X-like | Up |
| BraA02g020160.3C | SPAC4H3.01 | chaperone protein DnaJ-like | Up |
| BraA02g020740.3C | WSD1 | O-acyltransferase WSD1-like | Up |
| BraA02g021100.3C | At1g72540 | putative receptor-like protein kinase At1g72540 | Up |
| BraA02g021190.3C | TIF4A-3 | eukaryotic initiation factor 4A-3 | Up |
| BraA02g021570.3C | YPR091C | uncharacterized LOC103852830 | Up |
| BraA02g021970.3C | TTM1 | uridine-cytidine kinase C | Up |
| BraA02g022900.3C | SAUR50 | auxin-induced protein 15A-like | Up |
| BraA07g039660.3C | ERD14 | dehydrin ERD14-like | Up |
| BraA02g023690.3C | GSH2 | glutathione synthetase, chloroplastic-like | Up |
| BraA02g024390.3C | NIA2 | nitrate reductase [NADH], clone PBNBR1412-like | Up |
| BraA02g025230.3C | 2-Oct | organic cation/carnitine transporter 2 | Up |
| BraA02g025920.3C | VQ11 | VQ motif-containing protein 11-like | Up |
| BraA02g025960.3C | CYCA2-4 | cyclin-A2-4-like | Up |
| BraA01g008110.3C | PRMT11 | protein arginine N-methyltransferase 1.1 | Up |
| BraA06g009270.3C | At4g12090 | protein cornichon homolog 5 | Up |
| BraA09g063580.3C | At5g45960 | GDSL esterase/lipase At5g45960 | Up |
| BraA02g032740.3C | MCM3 | DNA replication licensing factor MCM3-like | Up |
| BraA02g033040.3C | ALMT13 | aluminum-activated malate transporter 13 | Up |
| BraA02g036260.3C | SOT12 | cytosolic sulfotransferase 12-like | Up |
| BraA02g036180.3C | AGL3 | agamous-like MADS-box protein AGL3 | Up |
| BraA02g035900.3C | RNS1 | ribonuclease 1 | Up |
| BraA02g035830.3C | ARPN | basic blue protein | Up |
| BraA02g035380.3C | PDF2.3 | defensin-like protein 1 | Up |
| BraA02g038730.3C | PHS1 | alpha-glucan phosphorylase 1-like | Up |
| BraA02g038630.3C | KIN11 | SNF1-related protein kinase catalytic subunit alpha KIN11-like | Up |
| BraA02g037710.3C | At3g27400 | putative pectate lyase 11 | Up |
| BraA09g002920.3C | LIR1 | light-regulated protein | Up |
| BraA05g039360.3C |  | uncharacterized LOC103854441 | Up |
| BraA02g039990.3C |  | uncharacterized protein DDB_G0271670-like | Up |
| BraA01g000880.3C | SRK2F | serine/threonine-protein kinase SRK2F | Up |
| BraA02g040860.3C |  | uncharacterized LOC103854672 | Up |
| BraA02g041180.3C | WRKY50 | probable WRKY transcription factor 50 | Up |
| BraA02g041220.3C | UBP12 | uncharacterized LOC103854707 | Up |
| BraA01g004450.3C | DAP | LL-diaminopimelate aminotransferase, chloroplastic | Up |
| BraA02g039560.3C | RLP7 | receptor-like protein 12 | Up |
| BraA02g042140.3C | APRR5 | two-component response regulator-like APRR5 | Up |
| BraA02g042210.3C | ATXR6 | histone-lysine N-methyltransferase ATXR6-like | Up |
| BraA02g042390.3C |  | uncharacterized LOC103854883 | Up |
| BraA02g043500.3C | CXE20 | probable carboxylesterase 120 | Up |
| BraA02g043580.3C | ATS3 | espin | Up |
| BraA02g043630.3C | PMEI11 | 21 kDa protein-like | Up |
| BraA02g043670.3C | COR2 | non-functional NADPH-dependent codeinone reductase 2-like | Up |
| BraA02g043760.3C | UBC3 | ubiquitin-conjugating enzyme E2 3 | Up |
| BraA05g041590.3C | HSFA1E | heat stress transcription factor A-1e | Up |
| BraA05g041610.3C | SPCC24B10.10c | ATPase family AAA domain-containing protein 3-A-like | Up |
| BraA05g041700.3C |  | uncharacterized LOC103855137 | Up |
| BraA05g042280.3C | At1g64760 | glucan endo-1,3-beta-glucosidase 8 | Up |
| BraA02g044360.3C | TOP3A | DNA topoisomerase 3-alpha | Up |
| BraA02g044540.3C |  | classical arabinogalactan protein 1-like | Up |
| BraA02g044640.3C |  | uncharacterized LOC103855246 | Up |
| BraA02g044760.3C | ABR1 | ethylene-responsive transcription factor ABR1-like | Up |
| BraA02g045300.3C | WAVH2 | uncharacterized LOC103855312 | Up |
| BraA03g000150.3C | LSC30 | ferritin-1, chloroplastic | Up |
| BraA03g000100.3C | LECRK62 | L-type lectin-domain containing receptor kinase VI.2 | Up |
| BraA03g000040.3C | GT-3A | trihelix transcription factor GT-3a-like | Up |
| BraA03g000940.3C | At5g02910 | F-box/LRR-repeat protein At5g02910-like | Up |
| BraA03g001500.3C |  | uncharacterized LOC103855560 | Up |
| BraA03g003120.3C | MYB29 | transcription factor MYB29-like | Up |
| BraA03g003200.3C | At3g50280 | uncharacterized acetyltransferase At3g50280-like | Up |
| BraA03g003250.3C | RPA1B | replication protein A 70 kDa DNA-binding subunit B | Up |
| BraA03g003880.3C | PUMP6 | mitochondrial uncoupling protein 6 | Up |
| BraA03g004620.3C | At5g11400 | putative inactive serine/threonine-protein kinase At5g11400 | Up |
| BraA03g005760.3C | IQD1 | protein IQ-DOMAIN 1-like | Up |
| BraA03g005990.3C | CHS3 | chalcone synthase 3-like | Up |
| BraA03g006400.3C | PNP2 | polyribonucleotide nucleotidyltransferase 2, mitochondrial | Up |
| BraA03g006720.3C |  | uncharacterized LOC103856121 | Up |
| BraA03g007270.3C | DET2 | very-long-chain enoyl-CoA reductase-like | Up |
| BraA03g008060.3C | CHAT | (Z)-3-hexen-1-ol acetyltransferase | Up |
| BraA03g008360.3C |  | uncharacterized LOC103856286 | Up |
| BraA10g020680.3C | STT3A | dolichyl-diphosphooligosaccharide--protein glycosyltransferase subunit STT3A-like | Up |
| BraA03g009390.3C | At5g20050 | probable receptor-like protein kinase At5g20050 | Up |
| BraA03g009710.3C | GER3 | germin-like protein subfamily 3 member 3 | Up |
| BraA03g009760.3C | TBL16 | protein trichome birefringence-like 16 | Up |
| BraA03g009850.3C | RAD51 | DNA repair protein RAD51 homolog 1 | Up |
| BraA03g009980.3C |  | hyccin-like | Up |
| BraA03g010330.3C | FAR4 | putative fatty acyl-CoA reductase 7 | Up |
| BraA03g011340.3C | SDT1 | uncharacterized protein C24B11.05-like | Up |
| BraA03g012460.3C | VIN3 | protein VERNALIZATION INSENSITIVE 3-like | Up |
| BraA03g012900.3C | MKK6 | mitogen-activated protein kinase kinase 6 | Up |
| BraA03g013160.3C | At4g11680 | E3 ubiquitin-protein ligase At4g11680-like | Up |
| BraA01g010420.3C | ILK1 | serine/threonine-protein kinase STY8 | Up |
| BraA01g001150.3C | PCKA | phosphoenolpyruvate carboxykinase [ATP] | Up |
| BraA03g013630.3C | At5g55050 | GDSL esterase/lipase At5g55050-like | Up |
| BraA03g013750.3C | THI1 | thiamine thiazole synthase, chloroplastic-like | Up |
| BraA03g013900.3C | GH3.6 | indole-3-acetic acid-amido synthetase GH3.6-like | Up |
| BraA03g014480.3C | COL5 | zinc finger protein CONSTANS-LIKE 9-like | Up |
| BraA03g014570.3C | CRF3 | ethylene-responsive transcription factor CRF3-like | Up |
| BraA03g015170.3C | DTX50 | protein DETOXIFICATION 50 | Up |
| BraA03g015390.3C | HIR4 | hypersensitive-induced response protein 4 | Up |
| BraA03g015770.3C | BHLH137 | transcription factor bHLH137 | Up |
| BraA03g016110.3C | WRKY25 | probable WRKY transcription factor 25 | Up |
| BraA01g010720.3C |  | uncharacterized LOC103857348 | Up |
| BraA03g017710.3C | PXG3 | probable peroxygenase 3 | Up |
| BraA03g018550.3C |  | uncharacterized LOC103857576 | Up |
| BraA03g018730.3C | FLA16 | fasciclin-like arabinogalactan protein 16 | Up |
| BraA09g043880.3C | SAP4 | zinc finger A20 and AN1 domain-containing stress-associated protein 4-like | Up |
| BraA03g018980.3C | RKF3 | probable LRR receptor-like serine/threonine-protein kinase RKF3 | Up |
| BraA03g019170.3C | XTH32 | probable xyloglucan endotransglucosylase/hydrolase protein 32 | Up |
| BraA03g015740.3C | MHF1 | centromere protein S | Up |
| BraA03g019670.3C | LECRK41 | L-type lectin-domain containing receptor kinase IV.1 | Up |
| BraA03g019700.3C | AKR4C9 | aldo-keto reductase family 4 member C9 | Up |
| BraA03g019820.3C | At3g10130 | uncharacterized LOC103857751 | Up |
| BraA03g020050.3C | PRA1B4 | PRA1 family protein B4 | Up |
| BraA03g020740.3C | MLO12 | MLO-like protein 12 | Up |
| BraA03g021240.3C | ELF4 | protein EARLY FLOWERING 4-like | Up |
| BraA04g019410.3C | CAM2 | calmodulin-5 | Up |
| BraA03g022310.3C | SD16 | receptor-like serine/threonine-protein kinase SD1-6 | Up |
| BraA03g022340.3C | COR15B | cold-regulated protein | Up |
| BraA03g022330.3C | COR15A | protein COLD-REGULATED 15B, chloroplastic-like | Up |
| BraA01g011310.3C |  | uncharacterized LOC103858030 | Up |
| BraA03g022910.3C | At2g43620 | endochitinase At2g43620 | Up |
| BraA03g023250.3C | MAP2A | methionine aminopeptidase 2A | Up |
| BraA03g023360.3C | Os03g0405500 | uncharacterized LOC103858132 | Up |
| BraA03g024500.3C | MYB12 | transcription factor MYB12-like | Up |
| BraA03g024780.3C | XBAT31 | putative E3 ubiquitin-protein ligase XBAT31 | Up |
| BraA03g024830.3C | PBL6 | probable serine/threonine-protein kinase RLCKVII | Up |
| BraA03g024840.3C | RAP2-7 | ethylene-responsive transcription factor RAP2-7-like | Up |
| BraA01g011510.3C | At4g20740 | pentatricopeptide repeat-containing protein At4g20740 | Up |
| BraA01g011540.3C | CML42 | calcium-binding protein CML42 | Up |
| BraA03g025530.3C | FLZ11 | uncharacterized LOC103858365 | Up |
| BraA03g025880.3C |  | protein MCM10 homolog | Up |
| BraA03g027930.3C | AZI1 | lipid transfer protein EARLI 1-like | Up |
| BraA03g028090.3C | OSCA1 | protein OSCA1 | Up |
| BraA03g028880.3C | SMD1B | small nuclear ribonucleoprotein Sm D1 | Up |
| BraA03g029500.3C |  | uncharacterized LOC103858800 | Up |
| BraA01g012000.3C |  | uncharacterized LOC103858880 | Up |
| BraA03g030840.3C | RGP1 | UDP-arabinopyranose mutase 1-like | Up |
| BraA01g012100.3C | SD18 | receptor-like serine/threonine-protein kinase SD1-8 | Up |
| BraA03g031170.3C | PUB21 | U-box domain-containing protein 21-like | Up |
| BraA03g031480.3C | RLP46 | LRR receptor-like serine/threonine-protein kinase GSO1 | Up |
| BraA03g032330.3C | RCI2A | hydrophobic protein RCI2A-like | Up |
| BraA03g032340.3C | RCI2A | hydrophobic protein RCI2A-like | Up |
| BraA03g032380.3C |  | uncharacterized LOC103859126 | Up |
| BraA03g032540.3C | MUTE | transcription factor MUTE | Up |
| BraA03g033240.3C | AIR12 | auxin-induced in root cultures protein 12-like | Up |
| BraA03g033340.3C | At3g07570 | cytochrome b561 and DOMON domain-containing protein At3g07570-like | Up |
| BraA03g034060.3C |  | probable L-type lectin-domain containing receptor kinase VI.1 | Up |
| BraA03g034320.3C | SCPL49 | serine carboxypeptidase-like 49 | Up |
| BraA03g034870.3C | TBL8 | protein trichome birefringence-like 8 | Up |
| BraA03g035980.3C | IQM2 | IQ domain-containing protein IQM2-like | Up |
| BraA03g036940.3C | BRIX1-1 | ribosome biogenesis protein BRX1-like | Up |
| BraA03g037080.3C | CNBF2260 | acyl-protein thioesterase 2-like | Up |
| BraA03g037790.3C |  | uncharacterized LOC103859698 | Up |
| BraA01g012730.3C | WRKY31 | probable WRKY transcription factor 31 | Up |
| BraA03g038140.3C |  | uncharacterized LOC103859728 | Up |
| BraA05g026570.3C | BHLH123 | transcription factor bHLH123-like | Up |
| BraA03g039730.3C | ELF3-2 | protein EARLY FLOWERING 3-like | Up |
| BraA03g040550.3C | ELIP1 | early light-induced protein 1, chloroplastic-like | Up |
| BraA03g041720.3C |  | dehydrin HIRD11 | Up |
| BraA03g042000.3C | GRP3S | glycine-rich protein 3 short isoform-like | Up |
| BraA03g042100.3C | MCM5 | DNA replication licensing factor MCM5 | Up |
| BraA03g042470.3C | At1g59620 | probable disease resistance protein At1g59620 | Up |
| BraA03g043240.3C | NFD4 | protein NUCLEAR FUSION DEFECTIVE 4 | Up |
| BraA03g043430.3C |  | tetracycline resistance protein, class E | Up |
| BraA03g043570.3C | DJC76 | uncharacterized LOC103860517 | Up |
| BraA03g044360.3C | APRR1 | two-component response regulator-like APRR1 | Up |
| BraA03g044380.3C | MYB28 | transcription factor MYB28 | Up |
| BraA09g007070.3C |  | putative glycine-rich cell wall structural protein 1 | Up |
| BraA03g044950.3C | LBD38 | LOB domain-containing protein 38 | Up |
| BraA03g045150.3C | SRK2D | serine/threonine-protein kinase SRK2D | Up |
| BraA03g045180.3C |  | nodulation protein H-like | Up |
| BraA03g045220.3C | GATL2 | probable galacturonosyltransferase-like 2 | Up |
| BraA03g045230.3C | CML41 | probable calcium-binding protein CML41 | Up |
| BraA03g045650.3C |  | macrophage migration inhibitory factor homolog | Up |
| BraA03g046070.3C | PUB22 | E3 ubiquitin-protein ligase PUB22-like | Up |
| BraA03g046230.3C | CYP74B2 | linolenate hydroperoxide lyase, chloroplastic | Up |
| BraA03g046340.3C | ARALYDRAFT_915236 | CASP-like protein 1D1 | Up |
| BraA03g046350.3C | At4g15630 | CASP-like protein 1E1 | Up |
| BraA03g047720.3C |  | uncharacterized LOC103860940 | Up |
| BraA03g047970.3C | IP5P2 | type I inositol polyphosphate 5-phosphatase 2 | Up |
| BraA07g019480.3C | PR5K | LEAF RUST 10 DISEASE-RESISTANCE LOCUS RECEPTOR-LIKE PROTEIN KINASE-like 2.1 | Up |
| BraA01g009760.3C |  | uncharacterized LOC103860982 | Up |
| BraA03g048600.3C | CYP707A1 | abscisic acid 8'-hydroxylase 1 | Up |
| BraA03g048740.3C | ALD1 | aminotransferase ALD1 | Up |
| BraA01g011400.3C | rnh202 | ribonuclease H2 subunit B-like | Up |
| BraA03g049330.3C | At4g20830 | reticuline oxidase-like protein | Up |
| BraA03g049360.3C |  | uncharacterized LOC103861205 | Up |
| BraA03g049390.3C | BHLH162 | transcription factor bHLH36-like | Up |
| BraA03g049440.3C | CAT1 | cationic amino acid transporter 1 | Up |
| BraA01g014320.3C | RTNLB1 | reticulon-like protein B1 | Up |
| BraA03g052550.3C | DREB1C | dehydration-responsive element-binding protein 1C-like | Up |
| BraA03g053740.3C | At4g27520 | vegetative cell wall protein gp1-like | Up |
| BraA03g053830.3C |  | uncharacterized LOC103861710 | Up |
| BraA01g014980.3C | VEP1 | 3-oxo-Delta(4,5)-steroid 5-beta-reductase | Up |
| BraA03g054960.3C | At4g29190 | zinc finger CCCH domain-containing protein 49 | Up |
| BraA03g055140.3C | PAO5 | probable polyamine oxidase 5 | Up |
| BraA03g055200.3C | CYP82A3 | cytochrome P450 CYP82D47-like | Up |
| BraA03g055250.3C | At3g55350 | putative nuclease HARBI1 | Up |
| BraA08g018130.3C | XTH18 | probable xyloglucan endotransglucosylase/hydrolase protein 18 | Up |
| BraA03g055670.3C | XTH17 | probable xyloglucan endotransglucosylase/hydrolase protein 17 | Up |
| BraA01g006900.3C | MYOB4 | myosin-binding protein 7-like | Up |
| BraA01g015240.3C | CLT2 | protein CLT2, chloroplastic | Up |
| BraA03g058690.3C | PGDH1 | D-3-phosphoglycerate dehydrogenase 1, chloroplastic-like | Up |
| BraA03g059560.3C | CST | probable serine/threonine-protein kinase Cx32, chloroplastic | Up |
| BraA03g059720.3C | NIP1 | NEP1-interacting protein 1-like | Up |
| BraA03g059910.3C | CYP84A1 | cytochrome P450 84A1-like | Up |
| BraA03g060300.3C | At4g36820 | calcium uniporter protein 1, mitochondrial-like | Up |
| BraA03g061090.3C |  | uncharacterized LOC103862588 | Up |
| BraA03g061240.3C | APK2 | adenylyl-sulfate kinase 2, chloroplastic-like | Up |
| BraA04g000120.3C | SMT | selenocysteine Se-methyltransferase | Up |
| BraA01g015650.3C | HVA22D | HVA22-like protein d | Up |
| BraA04g000450.3C | GRXC14 | glutaredoxin-C14-like | Up |
| BraA01g015680.3C | TPD1 | protein TAPETUM DETERMINANT 1 | Up |
| BraA04g001330.3C | BAP1 | BON1-associated protein 1-like | Up |
| BraA04g001850.3C |  | uncharacterized LOC103862884 | Up |
| BraA02g017800.3C | LECRK55 | L-type lectin-domain containing receptor kinase V.5-like | Up |
| BraA04g002470.3C | PRN1 | pirin-1-like | Up |
| BraA04g003170.3C | LPAT2 | 1-acyl-sn-glycerol-3-phosphate acyltransferase 2-like | Up |
| BraA04g004580.3C | PBL1 | probable receptor-like protein kinase At3g55450 | Up |
| BraA04g004730.3C | ABCB20 | ABC transporter B family member 20 | Up |
| BraA04g004780.3C | CLKR27 | 3-oxoacyl-[acyl-carrier-protein] reductase FabG-like | Up |
| BraA01g016210.3C |  | uncharacterized LOC103863220 | Up |
| BraA04g005440.3C | AED3 | aspartyl protease AED3-like | Up |
| BraA04g005640.3C | CDKB1-1 | cyclin-dependent kinase B1-1 | Up |
| BraA04g005660.3C | NPF8.1 | protein NRT1/ PTR FAMILY 8.1 | Up |
| BraA04g005760.3C | BSK6 | probable serine/threonine-protein kinase At5g41260 | Up |
| BraA04g000790.3C | At3g62230 | F-box protein At3g62230 | Up |
| BraA09g044640.3C | At3g53650 | histone H2B.8-like | Up |
| BraA04g005990.3C | PPA4 | soluble inorganic pyrophosphatase 4-like | Up |
| BraA01g016470.3C | GDU2 | protein GLUTAMINE DUMPER 2 | Up |
| BraA09g048330.3C | ZED1 | non-functional pseudokinase ZED1-like | Up |
| BraA04g007810.3C | SQP1,1 | squalene monooxygenase 1,1-like | Up |
| BraA04g010210.3C |  | microsomal glutathione S-transferase 3-like | Up |
| BraA04g010410.3C |  | uncharacterized LOC103863730 | Up |
| BraA01g016690.3C | PSD3 | phosphatidylserine decarboxylase proenzyme 3 | Up |
| BraA04g011510.3C |  | uncharacterized LOC103863822 | Up |
| BraA04g011960.3C |  | uncharacterized LOC103863898 | Up |
| BraA04g011990.3C |  | uncharacterized LOC103863900 | Up |
| BraA04g012170.3C |  | uncharacterized LOC103863916 | Up |
| BraA01g016880.3C | A70 | uncharacterized LOC103863917 | Up |
| BraA04g012470.3C | D6PK | serine/threonine-protein kinase D6PKL1 | Up |
| BraA04g013370.3C | LHT1 | lysine histidine transporter 1 | Up |
| BraA04g013980.3C |  | uncharacterized LOC103864083 | Up |
| BraA04g014140.3C | PGD3 | 6-phosphogluconate dehydrogenase, decarboxylating 2, chloroplastic | Up |
| BraA04g014610.3C | ABIL4 | protein ABIL4-like | Up |
| BraA04g015160.3C | At4g15540 | WAT1-related protein At4g15540 | Up |
| BraA04g015370.3C | At2g21120 | probable magnesium transporter NIPA6 | Up |
| BraA04g015750.3C | SFH3 | phosphatidylinositol/phosphatidylcholine transfer protein SFH3 | Up |
| BraA04g015840.3C | RL2 | protein RADIALIS-like 2 | Up |
| BraA09g055350.3C |  | glycine-rich RNA-binding protein GRP2A | Up |
| BraA04g016090.3C |  | uncharacterized LOC103864317 | Up |
| BraA04g016450.3C |  | classical arabinogalactan protein 2-like | Up |
| BraA04g016820.3C | ACS4 | 1-aminocyclopropane-1-carboxylate synthase 4 | Up |
| BraA07g037470.3C | SCPL12 | serine carboxypeptidase-like 13 | Up |
| BraA09g054330.3C | At2g23200 | probable receptor-like protein kinase At2g23200 | Up |
| BraA01g001890.3C | HHO5 | myb family transcription factor EFM | Up |
| BraA04g019780.3C | FD3 | ferredoxin, root R-B2 | Up |
| BraA04g019980.3C | AXS1 | UDP-D-apiose/UDP-D-xylose synthase 1 | Up |
| BraA10g009410.3C | ROG1 | putative lipase ROG1 | Up |
| BraA04g020370.3C | LBD11 | LOB domain-containing protein 11 | Up |
| BraA04g020920.3C | At2g29320 | tropinone reductase homolog At2g29310-like | Up |
| BraA04g020930.3C | CHY1 | 3-hydroxyisobutyryl-CoA hydrolase 1-like | Up |
| BraA04g021040.3C | TR | tropinone reductase-like | Up |
| BraA04g021180.3C | At2g29580 | zinc finger CCCH domain-containing protein 25 | Up |
| BraA04g021220.3C |  | uncharacterized LOC103864982 | Up |
| BraA04g021240.3C |  | uncharacterized LOC103864985 | Up |
| BraA04g021350.3C | MAPKKK17 | protein kinase byr2-like | Up |
| BraA05g013880.3C | CYP73A5 | trans-cinnamate 4-monooxygenase-like | Up |
| BraA03g016670.3C | ADS2 | delta-9 acyl-lipid desaturase 2-like | Up |
| BraA05g012760.3C | RLP28 | receptor-like protein 12 | Up |
|  | CLE42 | CLAVATA3/ESR (CLE)-related protein 42-like | Up |
| BraA04g025330.3C | PGSIP8 | putative glucuronosyltransferase PGSIP8 | Up |
| BraA09g006590.3C | DRT100 | probable LRR receptor-like serine/threonine-protein kinase At1g34110 | Up |
| BraA04g025900.3C | SBE2.1 | 1,4-alpha-glucan-branching enzyme 2-1, chloroplastic/amyloplastic-like | Up |
| BraA04g026160.3C | XTH32 | probable xyloglucan endotransglucosylase/hydrolase protein 32 | Up |
| BraA04g026350.3C | CP29B | RNA-binding protein CP29B, chloroplastic-like | Up |
| BraA04g026910.3C | At3g10130 | uncharacterized LOC103865641 | Up |
| BraA04g027060.3C |  | uncharacterized LOC103865654 | Up |
| BraA04g027110.3C | ANS | protein SRG1-like | Up |
| BraA04g027280.3C | At2g38640 | protein LURP-one-related 8-like | Up |
| BraA04g027390.3C | BASS2 | sodium/pyruvate cotransporter BASS2, chloroplastic | Up |
| BraA04g027480.3C | PHT1-4 | inorganic phosphate transporter 1-4 | Up |
| BraA04g027580.3C | MLO12 | MLO-like protein 12 | Up |
| BraA04g027700.3C | CSE | caffeoylshikimate esterase-like | Up |
| BraA04g027800.3C | ARALYDRAFT_903204 | CASP-like protein 4D2 | Up |
| BraA04g027810.3C | ARALYDRAFT_903205 | CASP-like protein 4D1 | Up |
| BraA04g027900.3C |  | uncharacterized LOC103865736 | Up |
| BraA09g006350.3C |  | protein SRC2 | Up |
| BraA04g028520.3C | AVT6D | putative sodium-coupled neutral amino acid transporter 7 | Up |
| BraA04g029300.3C | PER25 | peroxidase 25 | Up |
| BraA04g030230.3C | ATTI6 | defensin-like protein 197 | Up |
| BraA05g004340.3C | BGLU15 | beta-glucosidase 15 | Up |
| BraA01g017270.3C | FBA5 | fructose-bisphosphate aldolase 7, cytosolic | Up |
| BraA04g032300.3C | APRR9 | two-component response regulator-like APRR9 | Up |
| BraA05g000150.3C | COL13 | zinc finger protein CONSTANS-LIKE 13 | Up |
| BraA05g000480.3C | MYB12 | transcription factor MYB12 | Up |
| BraA05g000860.3C | ASY3 | uncharacterized LOC103866413 | Up |
| BraA05g001070.3C | APRR9 | two-component response regulator-like APRR9 | Up |
| BraA01g017420.3C | TIM22-3 | mitochondrial import inner membrane translocase subunit TIM22-2 | Up |
| BraA05g001940.3C | MYBC1 | transcription factor PCL1 | Up |
| BraA05g002180.3C | ARR8 | two-component response regulator ARR8-like | Up |
| BraA05g002850.3C | ATL41 | E3 ubiquitin-protein ligase ATL41 | Up |
| BraA05g003640.3C | NLP8 | protein NLP8-like | Up |
| BraA05g003700.3C | At2g43620 | endochitinase At2g43620 | Up |
| BraA05g003680.3C | CHI | endochitinase CHI-like | Up |
| BraA05g003970.3C | At1g64065 | late embryogenesis abundant protein At1g64065 | Up |
| BraA05g004260.3C |  | uncharacterized LOC103866789 | Up |
| BraA05g004350.3C | BGLU15 | beta-glucosidase 15-like | Up |
| BraA05g004340.3C | BGLU15 | beta-glucosidase 15-like | Up |
| BraA05g005370.3C | SOC1 | MADS-box protein SOC1 | Up |
| BraA05g006160.3C | At2g39920 | uncharacterized protein At2g39920 | Up |
| BraA05g006350.3C | PCS1 | aspartic proteinase PCS1-like | Up |
| BraA01g018050.3C | MIP1B | zinc finger protein HD1 | Up |
| BraA05g006850.3C | PHT1-4 | inorganic phosphate transporter 1-4-like | Up |
| BraA05g006880.3C | PHT1-4 | inorganic phosphate transporter 1-4-like | Up |
| BraA05g007080.3C | FAX3 | protein FATTY ACID EXPORT 3, chloroplastic | Up |
| BraA05g007410.3C | GT-3B | trihelix transcription factor GT-3b-like | Up |
| BraA05g007580.3C | NCER2 | neutral ceramidase | Up |
| BraA05g007780.3C | AKR4C9 | aldo-keto reductase family 4 member C9-like | Up |
| BraA05g007850.3C | At2g37700 | protein CER1-like 2 | Up |
| BraA01g002210.3C | RPT5 | ATPase family AAA domain-containing protein 3C | Up |
| BraA05g008200.3C | ABCG33 | ABC transporter G family member 33-like | Up |
| BraA05g008470.3C |  | uncharacterized LOC103867246 | Up |
| BraA05g008540.3C | UGT73C3 | UDP-glycosyltransferase 73C3-like | Up |
| BraA05g009890.3C | EDL3 | EID1-like F-box protein 3 | Up |
| BraA05g010210.3C | At2g34850 | putative UDP-arabinose 4-epimerase 2 | Up |
| BraA03g053850.3C |  | uncharacterized LOC103867478 | Up |
| BraA05g010950.3C | YNL011C | uncharacterized protein YNL011C | Up |
| BraA05g010990.3C | TBL37 | protein trichome birefringence-like 37 | Up |
| BraA05g011140.3C | DRMH1 | dormancy-associated protein homolog 1 | Up |
| BraA05g011290.3C | BUBR1 | mitotic spindle checkpoint protein BUBR1 | Up |
| BraA04g023600.3C | SUMO5 | small ubiquitin-related modifier 5-like | Up |
| BraA05g012620.3C |  | uncharacterized LOC103867632 | Up |
| BraA01g018740.3C | PYL10 | abscisic acid receptor PYL10 | Up |
| BraA04g025080.3C |  | uncharacterized LOC103867801 | Up |
| BraA05g013250.3C | At2g31390 | probable fructokinase-1 | Up |
| BraA01g019010.3C | EXPB3 | expansin-B3 | Up |
| BraA05g014490.3C | PH1 | pleckstrin homology domain-containing protein 1 | Up |
| BraA05g014510.3C |  | uncharacterized LOC103868016 | Up |
| BraA05g014810.3C | At2g29380 | probable protein phosphatase 2C 24 | Up |
| BraA05g014990.3C | WSD1 | O-acyltransferase WSD1 | Up |
| BraA08g031920.3C | GWD1 | alpha-glucan water dikinase 1, chloroplastic | Up |
| BraA08g032070.3C | UGT90A2 | UDP-glycosyltransferase 90A2 | Up |
| BraA02g027180.3C | MLO1 | MLO-like protein 1 | Up |
| BraA05g015170.3C | TAT | tyrosine aminotransferase | Up |
| BraA05g015310.3C | RBG2 | glycine-rich RNA-binding protein 2, mitochondrial-like | Up |
| BraA05g016930.3C | NTMC2T6.1 | C2 domain-containing protein At1g53590 | Up |
| BraA05g016960.3C | RHM2 | trifunctional UDP-glucose 4,6-dehydratase/UDP-4-keto-6-deoxy-D-glucose 3,5-epimerase/UDP-4-keto-L-rhamnose-reductase RHM2 | Up |
| BraA05g017350.3C | DRP5A | dynamin-related protein 5A | Up |
| BraA05g018070.3C | IOS1 | probable LRR receptor-like serine/threonine-protein kinase At1g51860 | Up |
| BraA05g018080.3C | At1g51860 | probable LRR receptor-like serine/threonine-protein kinase At1g51880 | Up |
| BraA05g018590.3C | At1g51060 | probable histone H2A.1 | Up |
| BraA05g018710.3C | TOR1L1 | microtubule-associated protein SPIRAL2-like | Up |
| BraA05g020050.3C |  | non-specific lipid-transfer protein 2 | Up |
| BraA05g020320.3C | CIPK17 | CBL-interacting serine/threonine-protein kinase 17-like | Up |
| BraA01g002360.3C | BGAL3 | beta-galactosidase 3 | Up |
| BraA02g019710.3C | RFK1 | probable LRR receptor-like serine/threonine-protein kinase At1g29720 | Up |
| BraA05g024380.3C | ELIP1 | early light-induced protein 1, chloroplastic | Up |
| BraA09g040610.3C | UGT85A2 | UDP-glycosyltransferase 85A2 | Up |
| BraA05g024940.3C | RH9 | DEAD-box ATP-dependent RNA helicase 9 | Up |
| BraA05g025070.3C | POP2 | gamma-aminobutyrate transaminase POP2, mitochondrial-like | Up |
| BraA05g025100.3C | POP2 | gamma-aminobutyrate transaminase POP2, mitochondrial | Up |
| BraA05g025150.3C | TPRP-F1 | 36.4 kDa proline-rich protein-like | Up |
| BraA05g025440.3C | spg1 | septum-promoting GTP-binding protein 1-like | Up |
| BraA05g026520.3C | At3g20670 | probable histone H2A.2 | Up |
| BraA05g026700.3C | GDPDL5 | glycerophosphodiester phosphodiesterase GDPDL5 | Up |
| BraA05g027150.3C | CYP705A22 | cytochrome P450 705A5-like | Up |
| BraA05g027650.3C |  | uncharacterized LOC103869433 | Up |
| BraA05g028150.3C | CHR25 | protein CHROMATIN REMODELING 25 | Up |
| BraA05g028780.3C | At4g11060 | single-stranded DNA-binding protein, mitochondrial | Up |
| BraA07g028670.3C | NMT1 | phosphoethanolamine N-methyltransferase 1 | Up |
| BraA05g030140.3C |  | uncharacterized LOC103869690 | Up |
| BraA05g030220.3C | Os05g0583200 | zinc finger BED domain-containing protein RICESLEEPER 1-like | Up |
| BraA05g030280.3C | C/VIF1 | cell wall / vacuolar inhibitor of fructosidase 1-like | Up |
| BraA05g030200.3C | Os03g0733400 | zinc finger BED domain-containing protein RICESLEEPER 2-like | Up |
| BraA05g031220.3C |  | uncharacterized LOC103869869 | Up |
| BraA05g031270.3C |  | uncharacterized LOC103869875 | Up |
| BraA05g031300.3C | At3g15720 | probable polygalacturonase At3g15720 | Up |
| BraA05g031920.3C |  | uncharacterized LOC103869945 | Up |
| BraA05g032420.3C | CYP72A13 | cytochrome P450 72A13-like | Up |
| BraA05g032670.3C |  | uncharacterized LOC103870031 | Up |
| BraA01g020680.3C |  | uncharacterized LOC103870066 | Up |
| BraA05g033100.3C | At3g13930 | dihydrolipoyllysine-residue acetyltransferase component 2 of pyruvate dehydrogenase complex, mitochondrial | Up |
| BraA01g020730.3C |  | proline-rich antigen homolog | Up |
| BraA01g002470.3C | CSA1 | disease resistance-like protein CSA1 | Up |
| BraA05g033880.3C | ATJ11 | chaperone protein dnaJ 11, chloroplastic-like | Up |
| BraA01g020860.3C | LEA41 | late embryogenesis abundant protein Lea5 | Up |
| BraA05g035550.3C | UGT76B1 | UDP-glycosyltransferase 76C2 | Up |
| BraA01g021060.3C | GRXS4 | monothiol glutaredoxin-S4 | Up |
| BraA01g021070.3C | GRXS4 | monothiol glutaredoxin-S4-like | Up |
| BraA05g036830.3C | CCR1 | serine/threonine-protein kinase-like protein CCR1 | Up |
| BraA05g036980.3C | At5g55720 | putative pectate lyase 21 | Up |
| BraA05g037060.3C |  | metallothionein-like protein type 2 | Up |
| BraA05g037190.3C | LECRK13 | flt3 receptor-interacting lectin-like | Up |
| BraA05g038000.3C | NSP2 | nitrile-specifier protein 5 | Up |
| BraA05g038020.3C | At3g07690 | glycerol-3-phosphate dehydrogenase [NAD(+)] At3g07690, cytosolic | Up |
| BraA05g038270.3C | AIR12 | auxin-induced in root cultures protein 12 | Up |
| BraA05g038570.3C |  | uncharacterized LOC103870706 | Up |
| BraA05g038630.3C | RH50 | DEAD-box ATP-dependent RNA helicase 50 | Up |
| BraA01g042550.3C | At3g05730 | defensin-like protein 205 | Up |
| BraA05g039750.3C | At3g05500 | REF/SRPP-like protein At3g05500 | Up |
| BraA05g041140.3C | OFUT21 | uncharacterized protein At1g04910 | Up |
| BraA06g000690.3C | DIR20 | dirigent protein 20-like | Up |
| BraA06g001340.3C | HEI10 | E3 ubiquitin-protein ligase CCNB1IP1 homolog | Up |
| BraA01g036690.3C | TSJT1 | stem-specific protein TSJT1-like | Up |
| BraA06g001730.3C |  | uncharacterized LOC103871178 | Up |
| BraA06g001810.3C | RPT3 | root phototropism protein 3 | Up |
| BraA06g002140.3C | PCR8 | protein PLANT CADMIUM RESISTANCE 8 | Up |
| BraA06g002280.3C | F-ATMBP | myrosinase-binding protein 2 | Up |
| BraA06g002360.3C | At1g51820 | probable LRR receptor-like serine/threonine-protein kinase At1g51820 | Up |
| BraA06g002370.3C | ILL4 | IAA-amino acid hydrolase ILR1-like 4 | Up |
| BraA06g002550.3C | MTPC4 | metal tolerance protein C4-like | Up |
| BraA06g003670.3C | PREP2 | presequence protease 2, chloroplastic/mitochondrial | Up |
| BraA06g003720.3C | CXE4 | probable carboxylesterase 4 | Up |
| BraA06g003730.3C | CXE5 | probable carboxylesterase 5 | Up |
| BraA06g003820.3C | ABF1 | ABSCISIC ACID-INSENSITIVE 5-like protein 4 | Up |
| BraA06g004760.3C |  | uncharacterized LOC103871493 | Up |
| BraA06g004790.3C | At1g48100 | polygalacturonase At1g48100 | Up |
| BraA06g004890.3C | BCA3 | beta carbonic anhydrase 3-like | Up |
| BraA06g005240.3C | GATA11 | GATA transcription factor 11 | Up |
| BraA07g003060.3C | TLP1 | thaumatin-like protein 1b | Up |
| BraA06g005750.3C | THA1 | probable low-specificity L-threonine aldolase 1 | Up |
| BraA06g005770.3C | PPCK1 | phosphoenolpyruvate carboxylase kinase 1 | Up |
| BraA06g006860.3C | DOGL4 | transcription factor TGA1-like | Up |
| BraA06g007090.3C | GRP23 | pentatricopeptide repeat-containing protein At1g10270-like | Up |
| BraA06g007200.3C | GSTU17 | glutathione S-transferase U17 | Up |
| BraA06g007900.3C |  | uncharacterized LOC103871884 | Up |
| BraA06g008060.3C | CMT1 | putative DNA (cytosine-5)-methyltransferase CMT1 | Up |
| BraA06g010070.3C | WRKY4 | probable WRKY transcription factor 4 | Up |
| BraA04g006730.3C | BIC1 | uncharacterized LOC103872141 | Up |
| BraA06g010930.3C | At1g14780 | MACPF domain-containing protein At1g14780 | Up |
| BraA06g011110.3C |  | uncharacterized LOC103872292 | Up |
| BraA06g011190.3C | At5g38780 | probable S-adenosylmethionine-dependent methyltransferase At5g38780 | Up |
| BraA06g012720.3C |  | uncharacterized LOC103872470 | Up |
| BraA06g012970.3C | GSTU24 | glutathione S-transferase U24 | Up |
| BraA06g012980.3C | GSTU25 | glutathione S-transferase U25-like | Up |
| BraA06g014610.3C | CPK10 | calcium-dependent protein kinase 10-like | Up |
| BraA06g014650.3C | MCH1 | uncharacterized LOC103872699 | Up |
| BraA06g014980.3C | At1g75140 | uncharacterized membrane protein At1g75140 | Up |
| BraA06g015050.3C | At1g19450 | sugar transporter ERD6-like 4 | Up |
| BraA06g015220.3C | CLH1 | chlorophyllase-1 | Up |
| BraA06g015970.3C | COR47 | dehydrin ERD10-like | Up |
| BraA06g016190.3C | RTEL1 | Fanconi anemia group J protein homolog | Up |
| BraA01g000270.3C | RD19A | cysteine protease RD19A | Up |
| BraA06g017950.3C | PER34 | peroxidase 34-like | Up |
| BraA06g018050.3C | AAE3 | oxalate--CoA ligase | Up |
| BraA06g018610.3C | CEP3 | KDEL-tailed cysteine endopeptidase CEP3 | Up |
| BraA06g019150.3C | PUB39 | U-box domain-containing protein 39 | Up |
| BraA06g019940.3C | PHS2 | alpha-glucan phosphorylase 2, cytosolic | Up |
| BraA06g020660.3C | At5g59910 | histone H2B.7 | Up |
| BraA01g023850.3C | BIA1 | BAHD acyltransferase At5g47980-like | Up |
| BraA06g024520.3C | At3g48460 | GDSL esterase/lipase At3g48460-like | Up |
| BraA06g023570.3C | ZAT7 | zinc finger protein ZAT8-like | Up |
| BraA01g025170.3C |  | A-agglutinin anchorage subunit | Up |
| BraA06g027110.3C | SRK2H | serine/threonine-protein kinase SRK2H | Up |
| BraA06g027460.3C | SAL3 | probable SAL3 phosphatase | Up |
| BraA06g027770.3C |  | classical arabinogalactan protein 1-like | Up |
| BraA06g028200.3C | ABR1 | ethylene-responsive transcription factor ABR1 | Up |
| BraA06g028820.3C | DTX27 | protein DETOXIFICATION 27-like | Up |
| BraA06g029570.3C | BT4 | BTB/POZ and TAZ domain-containing protein 4 | Up |
| BraA06g031540.3C | SQP1,2 | squalene monooxygenase 1,2 | Up |
| BraA06g031850.3C | APRR5 | two-component response regulator-like APRR5 | Up |
| BraA06g032430.3C | CIPK25 | CBL-interacting serine/threonine-protein kinase 25 | Up |
| BraA06g032750.3C | At5g25470 | B3 domain-containing protein At5g25470-like | Up |
| BraA06g033580.3C | LON1 | lon protease homolog 1, mitochondrial | Up |
| BraA06g034100.3C |  | myosin-10 | Up |
| BraA06g034230.3C | At5g27980 | late embryogenesis abundant protein 47-like | Up |
| BraA01g024260.3C | DCD1 | deoxycytidylate deaminase | Up |
| BraA05g023210.3C | PKP3 | plastidial pyruvate kinase 3, chloroplastic | Up |
| BraA06g035600.3C | GLR1.3 | glutamate receptor 1.2-like | Up |
| BraA06g035420.3C | RIN4 | RPM1-interacting protein 4 | Up |
| BraA06g035030.3C |  | uncharacterized LOC103875013 | Up |
| BraA06g035000.3C | ATJ49 | chaperone protein dnaJ 49 | Up |
| BraA06g036970.3C | At3g28510 | AAA-ATPase At3g28510-like | Up |
| BraA06g037440.3C | cdt2 | denticleless protein homolog | Up |
| BraA06g037620.3C | At3g27400 | putative pectate lyase 11 | Up |
| BraA06g037640.3C | SDH2-2 | succinate dehydrogenase [ubiquinone] iron-sulfur subunit 1, mitochondrial-like | Up |
| BraA06g038620.3C | NIMIN-2 | protein NIM1-INTERACTING 2 | Up |
| BraA06g038930.3C | At2g04925 | defensin-like protein 292 | Up |
| BraA06g039490.3C | RNS1 | ribonuclease 1-like | Up |
| BraA01g034860.3C | PYD1 | dihydropyrimidine dehydrogenase (NADP(+)), chloroplastic-like | Up |
| BraA03g043740.3C | MAM1 | methylthioalkylmalate synthase 1, chloroplastic | Up |
| BraA03g040840.3C | MYB90 | transcription factor MYB114 | Up |
| BraA04g027230.3C | WRKY33 | probable WRKY transcription factor 33 | Up |
| BraAnng003710.3C | WRKY23 | probable WRKY transcription factor 23 | Up |
| BraA05g014020.3C | WRKY25 | probable WRKY transcription factor 25 | Up |
| BraA03g048120.3C | WRKY28 | probable WRKY transcription factor 28 | Up |
| BraA06g034700.3C | WRKY48 | probable WRKY transcription factor 48 | Up |
| BraA05g000580.3C | WRKY23 | probable WRKY transcription factor 23 | Up |
| BraA07g018740.3C | ARF10 | auxin response factor 10 | Down |
| BraA05g007500.3C | AUX1 | auxin transporter protein 1 | Down |
| BraA06g013980.3C | RVE7 | protein REVEILLE 7 | Down |
| BraA03g021530.3C | EXPA8 | expansin | Down |
| BraA03g005120.3C | HSP17.6 | 17.6 kDa class II heat shock protein | Down |
| BraA05g038050.3C | COL9 | zinc finger protein CONSTANS-LIKE 9 | Down |
| BraA02g035350.3C | IDD4 | protein indeterminate-domain 4, chloroplastic | Down |
| BraA06g040190.3C |  | uncharacterized LOC103827552 | Down |
| BraA06g040630.3C |  | uncharacterized LOC103827593 | Down |
| BraA01g023460.3C | PP2A10 | protein PHLOEM PROTEIN 2-LIKE A10 | Down |
| BraA06g041230.3C | ERF2 | ethylene-responsive transcription factor 2 | Down |
| BraA06g043250.3C | GLK2 | transcription activator GLK2-like | Down |
| BraA06g044700.3C | CRK26 | cysteine-rich receptor-like protein kinase 26 | Down |
| BraA06g044690.3C |  | auxin-induced protein X15-like | Down |
| BraA01g022980.3C | ACLA_070510 | 3-oxoacyl-[acyl-carrier-protein] reductase FabG-like | Down |
| BraA01g043450.3C | P23-2 | uncharacterized protein At3g03773 | Down |
| BraA07g001540.3C | HSP18.5 | 18.5 kDa class IV heat shock protein | Down |
| BraA07g002430.3C | HBI1 | transcription factor HBI1-like | Down |
| BraA07g002590.3C | BZIP2 | bZIP transcription factor 2 | Down |
| BraA07g005530.3C | KCS8 | 3-ketoacyl-CoA synthase 8 | Down |
| BraA07g008580.3C | MBF1C | multiprotein-bridging factor 1c | Down |
| BraA07g008820.3C |  | golgin subfamily A member 6-like protein 2 | Down |
| BraA01g027130.3C | MED37E | probable mediator of RNA polymerase II transcription subunit 37e | Down |
| BraA07g009310.3C | SBP3 | selenium-binding protein 3 | Down |
| BraA07g009350.3C | TMK4 | receptor-like kinase TMK4 | Down |
| BraA04g007650.3C | CBK1 | serine/threonine-protein kinase tricorner | Down |
| BraA01g027230.3C | BCA6 | beta carbonic anhydrase 6, mitochondrial | Down |
| BraA07g010650.3C |  | uncharacterized LOC103828817 | Down |
| BraA07g012750.3C | MYB62 | transcription factor MYB24-like | Down |
|  |  | uncharacterized LOC103829175 | Down |
| BraA08g025670.3C |  | uncharacterized LOC103829176 | Down |
| BraA08g025930.3C | MPSR1 | E3 ubiquitin-protein ligase RING1-like | Down |
| BraA09g039860.3C | GGAT1 | glutamate--glyoxylate aminotransferase 1-like | Down |
| BraA07g014390.3C | PUB11 | U-box domain-containing protein 11-like | Down |
| BraA07g015500.3C | ARP1 | RNA-binding protein 24-like | Down |
| BraA07g015920.3C | SBT5.1 | subtilisin-like protease SBT5.1 | Down |
| BraA06g015100.3C | RL5 | protein RADIALIS-like 5 | Down |
| BraA07g016780.3C | EPS1 | uncharacterized acetyltransferase At3g50280 | Down |
| BraA07g017240.3C | STY17 | serine/threonine-protein kinase HT1 | Down |
| BraA07g018970.3C | KCS12 | 3-ketoacyl-CoA synthase 12-like | Down |
| BraA01g028550.3C |  | uncharacterized LOC103830077 | Down |
| BraA07g024180.3C | ATL4 | E3 ubiquitin-protein ligase ATL4-like | Down |
| BraA07g024750.3C | PYL7 | abscisic acid receptor PYL9-like | Down |
| BraA07g025620.3C | APT2 | adenine phosphoribosyltransferase 2 | Down |
| BraA07g026390.3C | PSBR | photosystem II 10 kDa polypeptide, chloroplastic | Down |
| BraA07g026730.3C |  | uncharacterized LOC103830535 | Down |
| BraA07g026770.3C |  | ABC transporter F family member 4-like | Down |
| BraA07g027110.3C | ACR3 | ACT domain-containing protein ACR3-like | Down |
| BraA07g027230.3C |  | uncharacterized LOC103830595 | Down |
| BraA07g027360.3C | PILS3 | protein PIN-LIKES 3-like | Down |
| BraA07g027490.3C | RLP15 | receptor like protein 30-like | Down |
| BraA07g027870.3C | At1g01500 | uncharacterized protein At1g01500-like | Down |
| BraA07g028020.3C | FLZ13 | protein MARD1-like | Down |
| BraA07g028030.3C | ERF018 | ethylene-responsive transcription factor ERF018-like | Down |
| BraA07g028060.3C | ARR15 | two-component response regulator ARR15 | Down |
| BraA07g038220.3C | CLPB1 | chaperone protein ClpB1-like | Down |
| BraA07g028720.3C | CSE | monoglyceride lipase-like | Down |
| BraA07g029030.3C | SAMC2 | solute carrier family 25 member 44-like | Down |
| BraA07g029460.3C | IP5P3 | type IV inositol polyphosphate 5-phosphatase 3-like | Down |
| BraA07g030750.3C | COL6 | zinc finger protein CONSTANS-LIKE 6-like | Down |
| BraA01g029450.3C |  | uncharacterized LOC103830973 | Down |
| BraA07g030850.3C | At1g68190 | putative zinc finger protein At1g68190 | Down |
| BraA07g031060.3C |  | uncharacterized LOC103831001 | Down |
| BraA07g031160.3C | UVR8 | ultraviolet-B receptor UVR8-like | Down |
| BraA07g031170.3C | PSBY | photosystem II core complex proteins psbY, chloroplastic-like | Down |
| BraA07g031340.3C | At1g67360 | REF/SRPP-like protein At1g67360 | Down |
| BraA07g033220.3C | ABCI17 | ABC transporter I family member 17 | Down |
| BraA07g033570.3C |  | uncharacterized LOC103831257 | Down |
| BraA07g034110.3C | At1g69160 | protein BIG GRAIN 1-like E | Down |
| BraA07g034970.3C | WDL7 | protein WVD2-like 7 | Down |
| BraA07g035110.3C | POT6 | potassium transporter 6 | Down |
| BraA07g035850.3C | At5g08430 | uncharacterized protein At5g08430-like | Down |
| BraA02g020610.3C | SBT3.4 | subtilisin-like protease SBT3.16 | Down |
| BraA01g003270.3C | PIP2-7 | aquaporin PIP2-7 | Down |
| BraA02g020930.3C | PSY1R | tyrosine-sulfated glycopeptide receptor 1 | Down |
| BraA07g036810.3C | GFA2 | chaperone protein DnaJ | Down |
| BraA07g036910.3C |  | uncharacterized LOC103831761 | Down |
| BraA07g037000.3C | DRG2 | developmentally-regulated G-protein 2 | Down |
| BraA07g037140.3C | N | TMV resistance protein N-like | Down |
| BraA07g037480.3C | KTI1 | bark lectin-like | Down |
| BraA07g038220.3C | CLPB1 | chaperone protein ClpB1 | Down |
| BraA07g038560.3C |  | transcription factor MYB1R1 | Down |
| BraA07g038650.3C | FLZ13 | uncharacterized LOC103831953 | Down |
| BraA07g039240.3C | GASA1 | gibberellin-regulated protein 1 | Down |
| BraA07g039320.3C | AFP4 | defensin-like protein 4 | Down |
| BraA07g039970.3C | ATL8 | RING-H2 finger protein ATL8 | Down |
| BraA07g040200.3C | CP12-3 | calvin cycle protein CP12-3, chloroplastic | Down |
| BraA07g013850.3C |  | uncharacterized LOC103832147 | Down |
| BraA07g041310.3C |  | uncharacterized LOC103832299 | Down |
| BraA07g041840.3C |  | pathogen-related protein | Down |
| BraA07g042620.3C | HDR1 | uncharacterized LOC103832442 | Down |
| BraA07g042640.3C | ATJ8 | chaperone protein dnaJ 8, chloroplastic-like | Down |
| BraA08g001230.3C | HSP17.4B | 17.4 kDa class III heat shock protein-like | Down |
| BraA08g001380.3C | SRF6 | protein STRUBBELIG-RECEPTOR FAMILY 6 | Down |
| BraA08g001560.3C | GIL1 | uncharacterized LOC103832669 | Down |
| BraA08g000300.3C | CSLE1 | cellulose synthase-like protein E1 | Down |
| BraA08g003090.3C | At1g50280 | BTB/POZ domain-containing protein At1g50280 | Down |
| BraA08g003960.3C | ATL76 | E3 ubiquitin-protein ligase ATL76-like | Down |
| BraA08g003970.3C | ATL76 | E3 ubiquitin-protein ligase ATL76-like | Down |
| BraA08g004530.3C | CIPK17 | CBL-interacting serine/threonine-protein kinase 17-like | Down |
| BraA08g006250.3C | ATJ20 | chaperone protein dnaJ 20, chloroplastic-like | Down |
| BraA10g022630.3C |  | uncharacterized LOC103833162 | Down |
| BraA08g006680.3C |  | caldesmon-like | Down |
| BraA08g002600.3C |  | myrosinase MA1-like | Down |
| BraA08g007700.3C | SHM7 | serine hydroxymethyltransferase 7 | Down |
| BraA08g007990.3C | BHLH80 | transcription factor bHLH80-like | Down |
| BraA01g030530.3C | DTX19 | protein DETOXIFICATION 19-like | Down |
| BraA02g020520.3C |  | uncharacterized LOC103833614 | Down |
| BraA03g014940.3C | CYP96A15 | alkane hydroxylase MAH1-like | Down |
| BraA08g009110.3C | G6PD1 | glucose-6-phosphate 1-dehydrogenase 1, chloroplastic | Down |
| BraA08g009190.3C |  | uncharacterized LOC103833701 | Down |
| BraA01g030750.3C | Os08g0127700 | pre-mRNA-splicing factor SLU7-like | Down |
| BraA08g009600.3C | ZPR1 | zinc finger protein ZPR1 | Down |
| BraA08g009760.3C | XTH15 | xyloglucan endotransglucosylase/hydrolase protein 15 | Down |
| BraA01g031080.3C |  | uncharacterized LOC103833968 | Down |
| BraA08g012840.3C | PUP10 | probable purine permease 9 | Down |
| BraA08g013360.3C |  | uncharacterized LOC103834108 | Down |
| BraA08g014750.3C | MSRB9 | peptide methionine sulfoxide reductase B9-like | Down |
| BraA01g031390.3C | EPFL5 | EPIDERMAL PATTERNING FACTOR-like protein 5 | Down |
| BraA03g054010.3C | BOB2 | protein BOBBER 2-like | Down |
| BraA08g015490.3C | PIP2-7 | aquaporin PIP2-7-like | Down |
| BraA08g015620.3C |  | PI-PLC X-box domain-containing protein DDB_G0293730 | Down |
| BraA08g015840.3C | CIF2 | uncharacterized LOC103834402 | Down |
| BraA08g016620.3C | PER47 | peroxidase 47-like | Down |
| BraA08g016980.3C |  | ABC transporter F family member 4-like | Down |
| BraA08g017930.3C |  | uncharacterized LOC103834656 | Down |
| BraA09g037520.3C |  | uncharacterized LOC103834672 | Down |
| BraA08g018140.3C | XTH24 | xyloglucan endotransglucosylase/hydrolase protein 24-like | Down |
| BraA08g018210.3C | AHA2 | ATPase 2, plasma membrane-type | Down |
| BraA08g018600.3C | At4g29360 | glucan endo-1,3-beta-glucosidase 12-like | Down |
| BraA08g019130.3C | RMA2 | E3 ubiquitin-protein ligase RMA2-like | Down |
| BraA08g019150.3C |  | uncharacterized LOC103834773 | Down |
| BraA08g019320.3C |  | uncharacterized LOC103834795 | Down |
| BraA08g019330.3C |  | uncharacterized LOC103834798 | Down |
| BraA08g019420.3C | TSJT1 | stem-specific protein TSJT1-like | Down |
| BraA08g019700.3C | VTC2 | GDP-L-galactose phosphorylase 1-like | Down |
| BraA08g019950.3C | PR-1 | pathogenesis-related protein PR-1-like | Down |
| BraA08g020320.3C | At4g24780 | probable pectate lyase 18 | Down |
| BraA08g020380.3C | REN1 | rho GTPase-activating protein REN1-like | Down |
| BraA08g020520.3C | FD | protein FD-like | Down |
| BraA08g020710.3C |  | uncharacterized LOC103834943 | Down |
| BraA08g021100.3C | BEE2 | transcription factor BEE 2 | Down |
| BraA08g021090.3C |  | uncharacterized LOC103834974 | Down |
| BraA08g021120.3C | RL3 | protein RADIALIS-like 3 | Down |
| BraA08g021310.3C | At4g37220 | cold-regulated 413 plasma membrane protein 4 | Down |
| BraA08g021360.3C |  | uncharacterized LOC103835014 | Down |
| BraA08g021440.3C | SPT | transcription factor SPATULA-like | Down |
| BraA08g022000.3C | ATHB-16 | homeobox-leucine zipper protein ATHB-16-like | Down |
| BraA08g022310.3C |  | uncharacterized LOC103835106 | Down |
| BraA08g023450.3C | INT2 | probable inositol transporter 2 | Down |
| BraA08g023760.3C | RFK1 | probable LRR receptor-like serine/threonine-protein kinase At1g29720 | Down |
| BraA08g023860.3C | SAUR64 | auxin-responsive protein SAUR64-like | Down |
| BraA08g025490.3C | COL16 | zinc finger protein CONSTANS-LIKE 16-like | Down |
| BraA08g019890.3C | KRP3 | cyclin-dependent kinase inhibitor 3-like | Down |
| BraA08g025830.3C |  | uncharacterized LOC103835604 | Down |
| BraA08g025870.3C | PHO1-H8 | phosphate transporter PHO1 homolog 8-like | Down |
| BraA08g026220.3C | WDL7 | protein WVD2-like 7 | Down |
| BraA08g026660.3C | MLP28 | MLP-like protein 31 | Down |
| BraA08g026680.3C | MLP34 | MLP-like protein 31 | Down |
| BraA08g027950.3C | ARAC4 | rac-like GTP-binding protein ARAC4 | Down |
| BraA06g014310.3C | PDCB3 | PLASMODESMATA CALLOSE-BINDING PROTEIN 3-like | Down |
| BraA08g028670.3C | LNG2 | protein LONGIFOLIA 2-like | Down |
| BraA08g029420.3C | VQ1 | VQ motif-containing protein 1-like | Down |
| BraA08g029700.3C | HSP70-5 | heat shock 70 kDa protein 5-like | Down |
| BraA08g030080.3C | PAP3 | purple acid phosphatase 3 | Down |
| BraA08g030480.3C | SOT1 | cytosolic sulfotransferase 1-like | Down |
|  |  | uncharacterized LOC103836116 | Down |
| BraA08g031250.3C |  | uncharacterized LOC103836193 | Down |
| BraA08g033700.3C |  | loricrin | Down |
| BraA08g034670.3C | At1g04770 | protein SULFUR DEFICIENCY-INDUCED 2-like | Down |
|  |  | leucine-rich repeat extensin-like protein 3 | Down |
| BraA09g001500.3C | At1g56220 | dormancy-associated protein homolog 3-like | Down |
| BraA07g001680.3C |  | uncharacterized LOC103836826 | Down |
| BraA02g026820.3C | PPA1 | soluble inorganic pyrophosphatase 1 | Down |
| BraA09g000900.3C | SDG41 | protein SET DOMAIN GROUP 41 | Down |
| BraA09g000120.3C | UGT72B1 | UDP-glycosyltransferase 72B1 | Down |
| BraA09g000350.3C | At1g01540 | probable serine/threonine-protein kinase At1g01540 | Down |
| BraA09g000460.3C |  | uncharacterized protein At3g61260-like | Down |
| BraA01g033400.3C | At3g19850 | BTB/POZ domain-containing protein At3g19850 | Down |
| BraA09g005910.3C |  | uncharacterized LOC103837086 | Down |
| BraA09g005890.3C | EBF2 | EIN3-binding F-box protein 2-like | Down |
| BraA09g005760.3C |  | uncharacterized LOC103837104 | Down |
| BraA09g003790.3C |  | UPF0301 protein Plut_0637-like | Down |
| BraA01g003740.3C | BHLH63 | transcription factor bHLH63-like | Down |
| BraA09g004460.3C | DI19-7 | protein DEHYDRATION-INDUCED 19 homolog 7-like | Down |
| BraA09g006850.3C | PDCB2 | PLASMODESMATA CALLOSE-BINDING PROTEIN 1-like | Down |
| BraA09g008070.3C | PSAN | photosystem I reaction center subunit N, chloroplastic-like | Down |
| BraA09g008220.3C | RPT3 | root phototropism protein 3-like | Down |
| BraA01g003770.3C | At4g34480 | glucan endo-1,3-beta-glucosidase 7 | Down |
| BraA09g008930.3C | AZF1 | zinc finger protein AZF1-like | Down |
| BraA09g009290.3C |  | L-ascorbate oxidase homolog | Down |
| BraA07g017320.3C | DAR6 | protein DA1-related 6-like | Down |
| BraA07g004540.3C | KCS9 | 3-ketoacyl-CoA synthase 9-like | Down |
| BraA01g034160.3C | ATL78 | RING-H2 finger protein ATL78-like | Down |
| BraA01g034170.3C | ATL77 | RING-H2 finger protein ATL77-like | Down |
| BraA09g011090.3C | MCA2 | protein MID1-COMPLEMENTING ACTIVITY 2-like | Down |
| BraA09g011350.3C | HBI1 | transcription factor HBI1-like | Down |
| BraA09g011810.3C | TS1 | threonine synthase 1, chloroplastic-like | Down |
| BraA09g014950.3C | BLT | protein BRANCHLESS TRICHOME | Down |
| BraA09g013990.3C | BDG1 | uncharacterized LOC103838188 | Down |
| BraA09g013030.3C | TET10 | tetraspanin-10 | Down |
| BraA09g015830.3C |  | probable serine/threonine-protein kinase kinX | Down |
| BraA05g028520.3C | ACT2 | actin-2 | Down |
| BraA09g017130.3C | At1g48100 | polygalacturonase At1g48100 | Down |
| BraA09g017410.3C |  | uncharacterized LOC103838563 | Down |
| BraA09g036020.3C | NCL | uncharacterized LOC103838933 | Down |
| BraA09g037210.3C | AGL9 | agamous-like MADS-box protein AGL9 homolog | Down |
| BraA09g021230.3C | At5g45370 | WAT1-related protein At5g45370-like | Down |
| BraA09g023290.3C |  | uncharacterized LOC103839466 | Down |
| BraA01g035610.3C | At3g16900 | protein LURP-one-related 13 | Down |
|  | At3g17050 | glycine-rich cell wall structural protein | Down |
| BraA09g025320.3C | ALN | allantoinase | Down |
| BraA09g025690.3C | 1-Apr | 5'-adenylylsulfate reductase 1, chloroplastic | Down |
| BraA03g037610.3C | TCTP | translationally-controlled tumor protein homolog | Down |
| BraA09g025610.3C | CPK31 | calcium-dependent protein kinase 31-like | Down |
| BraA09g031570.3C | PHYL1.2 | phytolongin Phyl1.2 | Down |
| BraA09g031600.3C | At1g33340 | putative clathrin assembly protein At1g33340 | Down |
| BraA01g036030.3C | TIP2-1 | aquaporin TIP2-1-like | Down |
| BraA01g036040.3C |  | uncharacterized LOC103840105 | Down |
| BraA09g032080.3C | NQR | NADPH:quinone oxidoreductase-like | Down |
| BraA09g033440.3C | UAF30 | upstream activation factor subunit UAF30 | Down |
| BraA09g033480.3C | SPBC13G1.09 | bystin | Down |
| BraA09g033660.3C | EDA2 | lysosomal Pro-X carboxypeptidase-like | Down |
| BraA09g033740.3C | AGO2 | protein argonaute 2 | Down |
| BraA01g036180.3C | PDX12 | pyridoxal 5'-phosphate synthase-like subunit PDX1.2 | Down |
| BraA08g009810.3C | CYP97A3 | protein LUTEIN DEFICIENT 5, chloroplastic | Down |
| BraA09g034800.3C |  | uncharacterized LOC103840420 | Down |
| BraA09g034960.3C |  | calcyclin-binding protein | Down |
| BraA09g037920.3C | COL16 | zinc finger protein CONSTANS-LIKE 16-like | Down |
| BraA01g036650.3C | NAC056 | NAC transcription factor 56-like | Down |
| BraA09g039720.3C | At1g23390 | F-box/kelch-repeat protein At1g23390 | Down |
| BraA09g040010.3C | MLP31 | MLP-like protein 43 | Down |
| BraA09g040040.3C |  | leucine-rich repeat extensin-like protein 3 | Down |
| BraA09g040060.3C | PUB11 | U-box domain-containing protein 11 | Down |
| BraA09g040320.3C | P23-1 | uncharacterized protein OsI_027940-like | Down |
| BraA09g040470.3C | AGL80 | agamous-like MADS-box protein AGL80 | Down |
| BraA09g040490.3C | NPF5.16 | protein NRT1/ PTR FAMILY 5.16 | Down |
| BraA09g041650.3C | HR3 | RPW8-like protein 3 | Down |
| BraA09g041760.3C | CYP90B1 | cytochrome P450 90B1 | Down |
| BraA09g042060.3C | COR413PM2 | cold-regulated 413 plasma membrane protein 2 | Down |
| BraA09g042630.3C |  | uncharacterized LOC103841060 | Down |
| BraA06g036070.3C |  | uncharacterized LOC103841086 | Down |
| BraA09g043680.3C | OFP18 | transcription repressor OFP18 | Down |
| BraA09g043790.3C | ACA1 | alpha carbonic anhydrase 1, chloroplastic | Down |
| BraA09g046700.3C | PSR2 | CTD small phosphatase-like protein | Down |
| BraA09g047180.3C | SCAB3 | stomatal closure-related actin-binding protein 3 | Down |
| BraA09g049350.3C | CML4 | calmodulin-like protein 4 | Down |
| BraA09g050970.3C | PIP1.1 | aquaporin PIP1-1 | Down |
| BraA01g037260.3C | CID9 | polyadenylate-binding protein-interacting protein 9-like | Down |
| BraA09g051630.3C | GSVIVT00026920001 | probable polygalacturonase | Down |
| BraA09g051820.3C | At5g03970 | F-box protein At5g03970-like | Down |
| BraA04g000700.3C | At3g01520 | universal stress protein A-like protein | Down |
| BraA09g052510.3C | HSFA7B | heat stress transcription factor A-7b | Down |
| BraA09g053310.3C | CLPB4 | chaperone protein ClpB4, mitochondrial | Down |
| BraA09g053780.3C | ALDH11A3 | NADP-dependent glyceraldehyde-3-phosphate dehydrogenase-like | Down |
| BraA09g053890.3C | HHP3 | heptahelical transmembrane protein 3 | Down |
| BraA09g054150.3C |  | uncharacterized LOC103842404 | Down |
| BraA09g054170.3C | MES2 | methylesterase 2-like | Down |
| BraA09g054200.3C | SDT | spermidine sinapoyl-CoA acyltransferase-like | Down |
| BraA09g055600.3C | FBA1 | fructose-bisphosphate aldolase 1, chloroplastic-like | Down |
| BraA09g055750.3C |  | uncharacterized LOC103842551 | Down |
| BraA09g056160.3C | psi1 | dnaJ homolog subfamily B member 13 | Down |
| BraA09g056680.3C | BZR2 | protein BRASSINAZOLE-RESISTANT 2-like | Down |
| BraA09g057180.3C | MYB51 | transcription factor MYB51-like | Down |
| BraA09g057440.3C | OPR2 | putative 12-oxophytodienoate reductase-like protein 2A | Down |
| BraA01g044610.3C | PTB | uncharacterized LOC103842783 | Down |
| BraA09g057860.3C | At1g17200 | CASP-like protein 2A1 | Down |
| BraA09g058830.3C |  | uncharacterized LOC103842910 | Down |
| BraA09g059600.3C | MPSR1 | E3 ubiquitin-protein ligase RING1 | Down |
| BraA09g060000.3C | PGL1 | probable 6-phosphogluconolactonase 1 | Down |
| BraA09g060310.3C | RFL1 | disease resistance protein RFL1-like | Down |
| BraA09g059700.3C | FUT8 | probable fucosyltransferase 8 | Down |
| BraA09g061510.3C |  | lipase | Down |
| BraA09g062030.3C | BHLH74 | transcription factor bHLH74-like | Down |
| BraA09g062460.3C | RBOHB | respiratory burst oxidase homolog protein B | Down |
| BraA09g063280.3C |  | covalently-linked cell wall protein 14-like | Down |
| BraA09g064590.3C | GLR3.4 | glutamate receptor 3.4-like | Down |
| BraA09g065130.3C |  | uncharacterized LOC103843676 | Down |
| BraA09g065870.3C | HFR1 | transcription factor HFR1-like | Down |
| BraA10g000750.3C | KCS1 | 3-ketoacyl-CoA synthase 1-like | Down |
| BraAnng003690.3C | UPB1 | transcription factor UPBEAT1-like | Down |
| BraA09g066210.3C | PIP1-3 | aquaporin PIP1-3 | Down |
| BraAnng003750.3C | GOLS1 | galactinol synthase 1-like | Down |
| BraA09g000880.3C | ERF1 | ethylene-responsive transcription factor 1-like | Down |
| BraA10g006270.3C | TRM32 | protein TRM32-like | Down |
| BraA10g006050.3C | SR45A | serine/arginine-rich splicing factor SR45a | Down |
| BraA10g005580.3C |  | uncharacterized LOC103843997 | Down |
| BraA10g004480.3C | IP5P13 | type I inositol polyphosphate 5-phosphatase 13-like | Down |
| BraA10g003870.3C | PER3 | peroxidase 3-like | Down |
| BraA08g034370.3C |  | traB domain-containing protein-like | Down |
| BraA10g003270.3C | At1g04770 | protein SULFUR DEFICIENCY-INDUCED 2 | Down |
| BraA10g002850.3C | At4g14450 | uncharacterized protein At4g14450, chloroplastic | Down |
| BraA10g002450.3C | FLA9 | fasciclin-like arabinogalactan protein 9 | Down |
| BraA01g026480.3C | At1g54730 | sugar transporter ERD6-like 5 | Down |
| BraA10g001640.3C | At1g03010 | BTB/POZ domain-containing protein At1g03010 | Down |
| BraA09g002090.3C |  | uncharacterized LOC103844573 | Down |
| BraA10g001080.3C | HFR1 | transcription factor HFR1 | Down |
| BraA10g000950.3C | NAC005 | NAC domain-containing protein 5-like | Down |
| BraA10g000710.3C |  | uncharacterized LOC103844630 | Down |
| BraA05g003520.3C |  | uncharacterized LOC103844769 | Down |
| BraA04g032060.3C | TRY | transcription factor TRY | Down |
| BraA10g011850.3C | OPT9 | oligopeptide transporter 9 | Down |
| BraA10g012990.3C | At4g27130 | uncharacterized LOC103844945 | Down |
| BraA10g014420.3C |  | uncharacterized LOC103845089 | Down |
| BraA10g015880.3C | IBL1 | uncharacterized LOC103845212 | Down |
| BraA10g016120.3C |  | uncharacterized LOC103845246 | Down |
| BraA01g004460.3C |  | uncharacterized LOC103845257 | Down |
| BraA10g016660.3C | At5g58770 | dehydrodolichyl diphosphate synthase 2-like | Down |
| BraA10g017390.3C | UGT76E2 | UDP-glycosyltransferase 76E2 | Down |
| BraA01g038810.3C | MED37C | probable mediator of RNA polymerase II transcription subunit 37c | Down |
| BraA01g038840.3C | TRP3 | telomere repeat-binding protein 3 | Down |
| BraA10g018450.3C |  | uncharacterized LOC103845482 | Down |
| BraA10g019360.3C |  | putative protein TPRXL | Down |
| BraA10g019670.3C | AAO1 | indole-3-acetaldehyde oxidase | Down |
| BraA10g020960.3C |  | uncharacterized LOC103845761 | Down |
| BraA10g021080.3C | TSJT1 | stem-specific protein TSJT1-like | Down |
| BraA10g021480.3C | BAM9 | inactive beta-amylase 9 | Down |
| BraA10g022120.3C | SAUR20 | auxin-responsive protein SAUR21-like | Down |
| BraA02g006960.3C | RVE1 | protein REVEILLE 1-like | Down |
| BraA10g023170.3C | MYB20 | protein ODORANT1 | Down |
| BraA10g023670.3C | TBL19 | protein trichome birefringence-like 19 | Down |
| BraA10g024150.3C | ZHD8 | zinc-finger homeodomain protein 8 | Down |
| BraA09g002820.3C | CYP71B4 | cytochrome P450 71B4-like | Down |
| BraA10g024800.3C | At3g03770 | probable inactive leucine-rich repeat receptor-like protein kinase At3g03770 | Down |
| BraA02g005120.3C | PNS1 | protein PNS1-like | Down |
| BraA10g026190.3C | At5g12110 | elongation factor 1-beta 1 | Down |
| BraA10g026240.3C | HSP17.6 | 17.6 kDa class II heat shock protein-like | Down |
| BraA10g027040.3C |  | uncharacterized LOC103846755 | Down |
| BraA10g027130.3C | iqw1 | WD and tetratricopeptide repeats protein 1 | Down |
| BraA10g027140.3C | CIPK25 | CBL-interacting serine/threonine-protein kinase 5 | Down |
| BraA10g027630.3C | DOT3 | BTB/POZ domain-containing protein DOT3 | Down |
| BraA10g027690.3C | SULTR2 | sulfate transporter 2.1-like | Down |
| BraA10g027740.3C | TPPI | probable trehalose-phosphate phosphatase I | Down |
| BraA10g029870.3C |  | uncharacterized LOC103847066 | Down |
| BraA10g030120.3C | SDT | spermidine sinapoyl-CoA acyltransferase | Down |
| BraA03g003740.3C | CML12 | calumenin-A | Down |
| BraA10g031270.3C |  | uncharacterized protein C6C3.02c | Down |
| BraA10g031910.3C | BSMT1 | salicylate/benzoate carboxyl methyltransferase-like | Down |
| BraA10g031990.3C | At2g29580 | zinc finger CCCH domain-containing protein 25 | Down |
| BraA10g033000.3C | At5g02760 | probable protein phosphatase 2C 67 | Down |
| BraA10g033710.3C | LAX1 | auxin transporter-like protein 1 | Down |
| BraA10g024070.3C | CGS1 | cystathionine gamma-synthase 1, chloroplastic | Down |
| BraA06g041440.3C | At4g16560 | uncharacterized LOC103847617 | Down |
| BraA08g012010.3C | PPD | pheophorbidase-like | Down |
| BraA02g033660.3C | LIL3.2 | uncharacterized LOC103847919 | Down |
| BraA01g040680.3C | RVE8 | protein REVEILLE 8-like | Down |
| BraA05g020450.3C |  | uncharacterized LOC103848074 | Down |
| BraA03g064230.3C | 2-Apr | 5'-adenylylsulfate reductase 2, chloroplastic | Down |
| BraA03g063330.3C |  | 14 kDa proline-rich protein DC2.15-like | Down |
| BraA02g031420.3C |  | uncharacterized LOC103848479 | Down |
| BraA09g031890.3C | GTL1 | trihelix transcription factor GTL1 | Down |
| BraA06g000010.3C | UBC11 | ubiquitin-conjugating enzyme E2 11-like | Down |
| BraA02g030540.3C | GDPD3 | glycerophosphodiester phosphodiesterase GDPD3-like | Down |
| BraA02g030920.3C | VAB | VAN3-binding protein-like | Down |
| BraA02g031110.3C | GLK2 | transcription activator GLK2-like | Down |
| BraA05g040300.3C | DRT102 | DNA-damage-repair/toleration protein DRT102 | Down |
| BraA03g061960.3C | RXW8 | CSC1-like protein RXW8 | Down |
| BraA03g062560.3C | TPS21 | alpha-humulene/(-)-(E)-beta-caryophyllene synthase-like | Down |
| BraA01g029340.3C | At1g61610 | putative G-type lectin S-receptor-like serine/threonine-protein kinase At1g61610 | Down |
| BraA09g014290.3C | CBP60G | uncharacterized LOC103849694 | Down |
| BraA01g038390.3C |  | phosphatidylcholine transfer protein | Down |
| BraA02g039040.3C | COBL2 | COBRA-like protein 2 | Down |
| BraA01g041960.3C | GAL1 | galactokinase-like | Down |
| BraA08g011900.3C |  | uncharacterized LOC103849883 | Down |
| BraA01g042900.3C |  | uncharacterized LOC103850026 | Down |
| BraA07g019560.3C | RBL9 | RHOMBOID-like protein 9, chloroplastic | Down |
| BraA07g020140.3C |  | trichohyalin | Down |
| BraA07g020350.3C | CLC-A | chloride channel protein CLC-a-like | Down |
| BraA03g004250.3C | DTX28 | protein DETOXIFICATION 28-like | Down |
| BraA02g003260.3C |  | uncharacterized LOC103850350 | Down |
| BraA02g003050.3C | HSP70-10 | heat shock 70 kDa protein 10, mitochondrial | Down |
| BraA02g000110.3C | CIPK14 | CBL-interacting serine/threonine-protein kinase 14 | Down |
| BraA02g000130.3C | At5g01750 | protein LURP-one-related 15 | Down |
| BraA02g000140.3C | WUN1 | wound-induced protein 1 | Down |
| BraA02g000300.3C | DCR | uncharacterized acetyltransferase At3g50280-like | Down |
| BraA02g000590.3C | At5g02760 | probable protein phosphatase 2C 67 | Down |
| BraA02g000900.3C | HIPP06 | heavy metal-associated isoprenylated plant protein 3 | Down |
| BraA02g001870.3C |  | uncharacterized LOC103850602 | Down |
| BraA02g001960.3C |  | uncharacterized LOC103850614 | Down |
| BraA02g002040.3C | MYOB7 | myosin-binding protein 7-like | Down |
| BraA02g002140.3C | PHL5 | myb family transcription factor PHL5-like | Down |
| BraA10g030470.3C |  | uncharacterized LOC103850683 | Down |
| BraA02g002770.3C | SRM1 | transcription factor DIVARICATA-like | Down |
| BraA02g004150.3C | KEA6 | K(+) efflux antiporter 6-like | Down |
| BraA02g004310.3C | HSP17.6 | 17.6 kDa class II heat shock protein | Down |
| BraA02g004420.3C | F6'H2 | feruloyl CoA ortho-hydroxylase 1-like | Down |
| BraA02g004420.3C | F6'H2 | feruloyl CoA ortho-hydroxylase 1-like | Down |
| BraA02g004850.3C | NPF6.1 | protein NRT1/ PTR FAMILY 6.1 | Down |
| BraA01g005600.3C |  | uncharacterized LOC103850912 | Down |
| BraA02g006220.3C | BZIP43 | basic leucine zipper 43-like | Down |
| BraA02g006310.3C |  | mental retardation GTPase activating protein homolog 4-like | Down |
| BraA02g006570.3C | MYB20 | myb-related protein 308-like | Down |
| BraA02g006960.3C | RVE1 | protein REVEILLE 1-like | Down |
| BraA10g021510.3C |  | probable feruloyl esterase A | Down |
| BraA02g009660.3C |  | uncharacterized LOC103851430 | Down |
| BraA02g009680.3C | NAC089 | NAC domain-containing protein 89-like | Down |
| BraA02g010710.3C | MYB59 | transcription factor MYB59-like | Down |
| BraA02g011090.3C | UBC7 | ubiquitin-conjugating enzyme E2 7 | Down |
| BraA02g011180.3C |  | uncharacterized LOC103851594 | Down |
| BraA01g006200.3C | IGMT4 | indole glucosinolate O-methyltransferase 4-like | Down |
| BraA02g011440.3C | At5g58770 | dehydrodolichyl diphosphate synthase 2-like | Down |
| BraA02g011790.3C | PHOT2 | phototropin-2-like | Down |
| BraA01g006340.3C | CYP71B2 | cytochrome P450 71B2-like | Down |
| BraA02g012770.3C |  | uncharacterized LOC103851817 | Down |
| BraA02g013050.3C | HSP90-2 | heat shock protein 90-2-like | Down |
| BraA02g016910.3C |  | protein OPI10 homolog | Down |
| BraA02g016950.3C |  | uncharacterized LOC103852314 | Down |
| BraA02g017230.3C | LRK10L-2.8 | LEAF RUST 10 DISEASE-RESISTANCE LOCUS RECEPTOR-LIKE PROTEIN KINASE-like 2.8 | Down |
| BraA02g017750.3C |  | uncharacterized LOC103852412 | Down |
| BraA01g007110.3C |  | uncharacterized LOC103852450 | Down |
| BraA02g018130.3C | NPF2.3 | protein NRT1/ PTR FAMILY 2.3-like | Down |
|  |  | uncharacterized LOC103852463 | Down |
| BraA02g018470.3C | COL6 | zinc finger protein CONSTANS-LIKE 6 | Down |
| BraA02g019000.3C | At1g69160 | protein BIG GRAIN 1-like E | Down |
| BraA07g034300.3C | PAT22 | probable protein S-acyltransferase 22 | Down |
| BraA02g019240.3C | EXPA1 | expansin-A1 | Down |
| BraA07g029580.3C | RLP11 | receptor-like protein 12 | Down |
| BraA01g007420.3C | XTH17 | probable xyloglucan endotransglucosylase/hydrolase protein 17 | Down |
| BraA02g021080.3C |  | uncharacterized LOC103852786 | Down |
| BraA02g021620.3C | KTI1 | alpha-amylase/subtilisin inhibitor-like | Down |
| BraA02g022450.3C | GASA6 | gibberellin-regulated protein 6 | Down |
| BraA02g022640.3C | At1g01500 | uncharacterized protein At1g01500-like | Down |
| BraA02g022740.3C | BZIP44 | bZIP transcription factor 44-like | Down |
| BraA02g023030.3C |  | uncharacterized LOC103852976 | Down |
| BraA07g039290.3C | PR5K | thaumatin-like protein 1b | Down |
| BraA02g023780.3C | GT-2 | trihelix transcription factor GT-2-like | Down |
| BraA02g023950.3C | SKIP | SNW/SKI-interacting protein-like | Down |
| BraA02g024450.3C |  | uncharacterized LOC103853102 | Down |
| BraA02g024630.3C | UGT85A4 | UDP-glycosyltransferase 85A4 | Down |
| BraA02g026680.3C | ETC3 | MYB-like transcription factor ETC3 | Down |
| BraA02g026690.3C | UGT72B1 | UDP-glycosyltransferase 72B1-like | Down |
| BraA09g020900.3C | CRK41 | putative receptor-like protein kinase At4g00960 | Down |
| BraA02g021150.3C |  | uncharacterized LOC103853384 | Down |
| BraA02g033740.3C | ERF2 | ethylene-responsive transcription factor 2-like | Down |
| BraA06g038910.3C | NIK2 | protein NSP-INTERACTING KINASE 2-like | Down |
| BraA02g035550.3C | SAUR20 | auxin-responsive protein SAUR21-like | Down |
| BraA02g035400.3C |  | chromatin assembly factor 1 subunit A-like | Down |
| BraA02g034640.3C | PIN4 | auxin efflux carrier component 4 | Down |
| BraA02g034590.3C | At1g66480 | uncharacterized protein At1g66480-like | Down |
| BraA02g038860.3C | At3g29680 | BAHD acyltransferase At3g29680-like | Down |
| BraA09g003560.3C | CYP81D11 | cytochrome P450 81D11-like | Down |
| BraA02g038020.3C | At3g28100 | WAT1-related protein At3g28100 | Down |
| BraA02g036970.3C |  | uncharacterized LOC103854316 | Down |
| BraA05g039460.3C |  | probable serine/threonine-protein kinase kinX | Down |
| BraA05g039270.3C |  | uncharacterized LOC103854451 | Down |
| BraA05g039200.3C |  | uncharacterized LOC103854458 | Down |
| BraA02g039280.3C |  | uncharacterized LOC103854504 | Down |
| BraA02g039750.3C | GCH1 | GTP cyclohydrolase 1-like | Down |
| BraA02g039880.3C | SDI1 | protein SULFUR DEFICIENCY-INDUCED 1-like | Down |
| BraA02g040010.3C | GEX2 | protein GAMETE EXPRESSED 2 | Down |
| BraA02g040140.3C | BXL1 | beta-D-xylosidase 1-like | Down |
| BraA02g040260.3C | At1g06900 | nardilysin-like | Down |
| BraA02g040390.3C | BZIP63 | basic leucine zipper 63-like | Down |
| BraA02g041000.3C |  | uncharacterized LOC103854687 | Down |
| BraA08g002600.3C |  | myrosinase | Down |
| BraA02g041550.3C | RZFP34 | E3 ubiquitin-protein ligase MIEL1 | Down |
| BraA02g041780.3C | KNAT3 | homeobox protein knotted-1-like 3 | Down |
| BraA08g002600.3C |  | myrosinase-like | Down |
| BraA02g042480.3C | At5g08430 | uncharacterized protein At5g08430 | Down |
| BraA02g042560.3C | TCP7 | transcription factor TCP7 | Down |
| BraA02g042740.3C | MYB34 | transcription factor MYB34 | Down |
| BraA02g043000.3C |  | uncharacterized LOC103854948 | Down |
| BraA02g043360.3C | HSFB2A | heat stress transcription factor B-2a-like | Down |
| BraA02g043370.3C | HSFB2A | heat stress transcription factor B-2a-like | Down |
| BraA02g043810.3C | NPF2.11 | protein NRT1/ PTR FAMILY 2.11 | Down |
| BraA02g044050.3C | CYP94B1 | cytochrome P450 94B1-like | Down |
| BraA02g044290.3C |  | uncharacterized LOC103855064 | Down |
| BraA02g042730.3C | MYB34 | transcription factor MYB34-like | Down |
| BraA02g044390.3C | SAL2 | SAL2 phosphatase-like | Down |
| BraA02g044420.3C | PSAN | photosystem I reaction center subunit N, chloroplastic-like | Down |
| BraA07g006380.3C | At3g55350 | putative nuclease HARBI1 | Down |
| BraA03g000780.3C |  | uncharacterized LOC103855476 | Down |
| BraA03g001470.3C | BHLH101 | transcription factor bHLH101 | Down |
| BraA03g001610.3C | VTE5 | phytol kinase 1, chloroplastic-like | Down |
| BraA01g001020.3C |  | uncharacterized LOC103855772 | Down |
| BraA03g003820.3C | AAP2 | amino acid permease 2 | Down |
| BraA10g027530.3C | At5g10370 | ATP-dependent RNA helicase DEAH12, chloroplastic | Down |
| BraA03g004600.3C | TAO1 | probable disease resistance protein RPP1 | Down |
| BraA03g005670.3C | CRN | inactive leucine-rich repeat receptor-like protein kinase CORYNE | Down |
| BraA03g005870.3C | SMT1 | cycloartenol-C-24-methyltransferase | Down |
| BraA03g005950.3C | XTH5 | probable xyloglucan endotransglucosylase/hydrolase protein 5 | Down |
| BraA03g006820.3C | At5g15350 | lamin-like protein | Down |
| BraA03g006890.3C | BAD1 | ankyrin repeat-containing protein NPR4 | Down |
| BraA03g007280.3C |  | uncharacterized LOC103856178 | Down |
| BraA10g023420.3C | CSLA15 | probable mannan synthase 11 | Down |
| BraA03g007960.3C |  | uncharacterized LOC103856244 | Down |
| BraA03g008710.3C | MIEL1 | E3 ubiquitin-protein ligase MIEL1 | Down |
| BraA03g008720.3C | BAM9 | inactive beta-amylase 9 | Down |
| BraA03g008810.3C | At5g18770 | F-box/FBD/LRR-repeat protein At5g18770-like | Down |
| BraA03g009030.3C |  | uncharacterized LOC103856371 | Down |
| BraA03g011150.3C | MYB59 | transcription factor MYB59 | Down |
| BraA03g011320.3C | At5g59540 | 1-aminocyclopropane-1-carboxylate oxidase homolog 12-like | Down |
| BraA03g011630.3C | AIRP2 | uncharacterized LOC103856664 | Down |
| BraA03g012150.3C | At5g25090 | early nodulin-like protein 1 | Down |
| BraA03g012240.3C | CER3 | protein ECERIFERUM 3-like | Down |
| BraA03g012760.3C | GATA21 | GATA transcription factor 21-like | Down |
| BraA03g012770.3C |  | uncharacterized LOC103856793 | Down |
| BraA03g012780.3C | KUA1 | uncharacterized LOC103856794 | Down |
| BraA03g012920.3C |  | uncharacterized LOC103856812 | Down |
| BraA03g003600.3C | SRM1 | transcription factor DIVARICATA-like | Down |
| BraA03g012240.3C | CER3 | protein ECERIFERUM 3-like | Down |
| BraA03g013050.3C | WRKY2 | probable WRKY transcription factor 2 | Down |
| BraA03g013240.3C | NMNAT | nicotinamide/nicotinic acid mononucleotide adenylyltransferase | Down |
| BraA03g013530.3C |  | uncharacterized LOC103857001 | Down |
| BraA03g013680.3C | At4g27130 | protein translation factor SUI1 homolog | Down |
| BraA01g010560.3C | CCD4 | probable carotenoid cleavage dioxygenase 4, chloroplastic | Down |
| BraA03g015480.3C | HSP23.5 | 23.5 kDa heat shock protein, mitochondrial-like | Down |
| BraA03g016250.3C | CYP73A5 | trans-cinnamate 4-monooxygenase-like | Down |
| BraA03g016290.3C | RPT2 | root phototropism protein 2-like | Down |
| BraAnng000210.3C | ERF15 | ethylene-responsive transcription factor 15-like | Down |
| BraA03g017150.3C | PHO1-3 | SPX and EXS domain-containing protein 3 | Down |
| BraA03g017990.3C |  | uncharacterized LOC103857523 | Down |
| BraA03g018150.3C | AIR9 | 187-kDa microtubule-associated protein AIR9 | Down |
| BraA01g010920.3C |  | transmembrane protein 56 | Down |
| BraA03g019360.3C | PIP2-2 | aquaporin PIP2-2-like | Down |
| BraA03g016830.3C | REM14 | putative B3 domain-containing protein REM15 | Down |
| BraA05g009260.3C |  | glycine-rich protein DOT1-like | Down |
| BraA03g021010.3C | RCA | ribulose bisphosphate carboxylase/oxygenase activase, chloroplastic-like | Down |
| BraA03g021440.3C | At2g40450 | putative BTB/POZ domain-containing protein At2g40450 | Down |
| BraA03g021670.3C | RHC1A | probable E3 ubiquitin-protein ligase RHC1A | Down |
| BraA03g021820.3C | AVT1A | vacuolar amino acid transporter 1-like | Down |
| BraA03g022190.3C |  | uncharacterized LOC103858008 | Down |
| BraA03g022440.3C | PAR1 | transcription factor PAR1-like | Down |
| BraA03g022510.3C | PIF4 | transcription factor PIF4-like | Down |
| BraA03g023450.3C | CYCU4-1 | cyclin-U4-1-like | Down |
| BraA03g024000.3C | PIP1-2 | aquaporin PIP1-2-like | Down |
| BraA03g024640.3C | EFL1 | protein ELF4-LIKE 1 | Down |
| BraA03g025600.3C | HSFA2 | heat stress transcription factor A-2 | Down |
| BraA01g011600.3C | At4g20820 | cannabidiolic acid synthase-like 1 | Down |
| BraA03g027160.3C | HSP22.0 | 22.0 kDa heat shock protein | Down |
| BraA03g027650.3C | HSFB2B | heat stress transcription factor B-2b-like | Down |
| BraA03g027750.3C | PGRL1B | PGR5-like protein 1B, chloroplastic | Down |
| BraA03g027960.3C | EARLI1 | lipid transfer protein EARLI 1-like | Down |
| BraA03g028320.3C | MSRB5 | peptide methionine sulfoxide reductase B5 | Down |
| BraA03g028660.3C | XTH9 | xyloglucan endotransglucosylase/hydrolase protein 9 | Down |
| BraA03g028670.3C | GRH1 | GRR1-like protein 1 | Down |
| BraA03g028960.3C | At1g56220 | dormancy-associated protein homolog 3 | Down |
| BraA03g029800.3C | EDR4 | uncharacterized LOC103858833 | Down |
| BraA01g012030.3C | PSBQ2 | oxygen-evolving enhancer protein 3-1, chloroplastic | Down |
| BraA03g030660.3C | KIN10 | SNF1-related protein kinase catalytic subunit alpha KIN10-like | Down |
| BraA03g031240.3C | At5g17165 | uncharacterized LOC103858995 | Down |
| BraA03g031820.3C |  | pathogenesis-related protein PR-4-like | Down |
| BraA05g040100.3C |  | coiled-coil domain-containing protein 12-like | Down |
| BraA03g034180.3C | LTP6 | non-specific lipid-transfer protein 6-like | Down |
| BraA03g035360.3C | MED37C | probable mediator of RNA polymerase II transcription subunit 37c | Down |
| BraA01g012540.3C | DER2.1 | derlin-2.1 | Down |
| BraA03g035680.3C |  | uncharacterized LOC103859489 | Down |
| BraA03g035940.3C | CPN60B2 | chaperonin 60 subunit beta 2, chloroplastic | Down |
| BraA03g035960.3C |  | uncharacterized LOC103859520 | Down |
| BraA03g036080.3C | TMN7 | transmembrane 9 superfamily member 7-like | Down |
| BraA03g036450.3C | GLO2 | peroxisomal (S)-2-hydroxy-acid oxidase GLO2 | Down |
| BraA03g037010.3C |  | uncharacterized LOC103859629 | Down |
| BraA03g037440.3C | TIP2-1 | aquaporin TIP2-1-like | Down |
| BraA03g037560.3C |  | protein IQ-DOMAIN 14-like | Down |
| BraA01g012700.3C | 3-Apr | 5'-adenylylsulfate reductase 3, chloroplastic | Down |
| BraA03g037650.3C | RAP2-3 | ethylene-responsive transcription factor RAP2-3-like | Down |
|  | At3g17050 | glycine-rich cell wall structural protein-like | Down |
| BraA03g038060.3C | CNGC20 | probable cyclic nucleotide-gated ion channel 20, chloroplastic | Down |
| BraA03g038170.3C |  | uncharacterized LOC103859730 | Down |
| BraA03g038270.3C | At4g28100 | uncharacterized GPI-anchored protein At4g28100-like | Down |
| BraA03g038300.3C | At1g22950 | uncharacterized PKHD-type hydroxylase At1g22950-like | Down |
| BraA03g038530.3C | ATL77 | RING-H2 finger protein ATL77-like | Down |
| BraA03g038660.3C |  | uncharacterized LOC103859788 | Down |
| BraA03g038810.3C | CYP707A4 | abscisic acid 8'-hydroxylase 4-like | Down |
| BraA03g038820.3C | ABF4 | ABSCISIC ACID-INSENSITIVE 5-like protein 7 | Down |
| BraA03g038910.3C | AZF2 | zinc finger protein AZF2-like | Down |
| BraA03g041150.3C | NAKR2 | uncharacterized LOC103860035 | Down |
| BraA01g013250.3C | At4g22670 | FAM10 family protein At4g22670 | Down |
| BraA03g041520.3C | FKBP62 | peptidyl-prolyl cis-trans isomerase FKBP62 | Down |
| BraA03g042020.3C | GRP3 | glycine-rich protein 3 short isoform | Down |
| BraA03g042140.3C |  | uncharacterized LOC103860346 | Down |
| BraA01g013460.3C | TPX2 | protein TPX2-like | Down |
| BraA03g042690.3C | GASA7 | gibberellin-regulated protein 7-like | Down |
| BraA03g042980.3C |  | uncharacterized LOC103860440 | Down |
| BraA09g031720.3C | CRK11 | cysteine-rich receptor-like protein kinase 11 | Down |
| BraA03g044170.3C | PDCB1 | PLASMODESMATA CALLOSE-BINDING PROTEIN 1 | Down |
| BraA03g044400.3C | At5g61440 | thioredoxin-like 1-2, chloroplastic | Down |
| BraA08g001230.3C | HSP17.4B | 17.4 kDa class III heat shock protein-like | Down |
| BraA03g045590.3C | DGAT2 | diacylglycerol O-acyltransferase 2-like | Down |
| BraA03g045680.3C | SD25 | G-type lectin S-receptor-like serine/threonine-protein kinase SD2-5 | Down |
| BraA03g046120.3C | ABCG43 | ABC transporter G family member 42-like | Down |
| BraA03g046580.3C | SWEET17 | bidirectional sugar transporter SWEET17 | Down |
| BraA08g011490.3C |  | dynein light chain 2, cytoplasmic-like | Down |
| BraA03g047100.3C | CSLA1 | probable mannan synthase 1 | Down |
| BraA03g047260.3C | EXLB1 | expansin-like B1 | Down |
| BraA03g047290.3C | EXLB1 | expansin-like B1 | Down |
| BraA03g048180.3C | At4g18970 | GDSL esterase/lipase At4g18970 | Down |
| BraA03g042430.3C |  | uncharacterized LOC103861102 | Down |
| BraA01g014070.3C |  | uncharacterized LOC103861113 | Down |
| BraA03g043790.3C |  | shematrin-like protein 2 | Down |
| BraA03g049500.3C | HSA32 | protein HEAT-STRESS-ASSOCIATED 32 | Down |
| BraA03g049520.3C | CRK29 | cysteine-rich receptor-like protein kinase 29 | Down |
| BraA03g049730.3C | BGLU47 | beta-glucosidase 47-like | Down |
| BraA03g049900.3C | 3-Apr | 5'-adenylylsulfate reductase 3, chloroplastic-like | Down |
| BraA03g050250.3C | APT3 | adenine phosphoribosyltransferase 3 | Down |
| BraA03g050630.3C |  | uncharacterized LOC103861350 | Down |
| BraA09g027280.3C | SGT1A | protein SGT1 homolog A | Down |
| BraA03g051510.3C |  | uncharacterized LOC103861442 | Down |
| BraA03g051520.3C |  | uncharacterized LOC103861443 | Down |
| BraA03g051570.3C |  | uncharacterized LOC103861449 | Down |
| BraA03g051610.3C | NLP7 | protein NLP7-like | Down |
| BraA03g052350.3C | PMEI7 | 21 kDa protein-like | Down |
| BraA03g052840.3C | CIA2 | protein CHLOROPLAST IMPORT APPARATUS 2-like | Down |
| BraA03g053590.3C |  | dihydroflavonol-4-reductase-like | Down |
| BraA08g019150.3C |  | wound-responsive protein-related | Down |
| BraA03g054310.3C | RMA2 | E3 ubiquitin-protein ligase RMA2-like | Down |
| BraA03g054350.3C |  | uncharacterized LOC103861824 | Down |
| BraA03g055650.3C | XTH24 | xyloglucan endotransglucosylase/hydrolase protein 24-like | Down |
| BraA01g015070.3C | HSP70-6 | heat shock 70 kDa protein 6, chloroplastic | Down |
| BraA03g056230.3C | UBP24 | ubiquitin carboxyl-terminal hydrolase 24-like | Down |
| BraA03g056640.3C |  | uncharacterized LOC103862060 | Down |
| BraA03g057240.3C |  | uncharacterized LOC103862117 | Down |
| BraA03g057860.3C | PER47 | peroxidase 47 | Down |
|  |  | cysteine-rich and transmembrane domain-containing protein A | Down |
| BraA03g058720.3C | DTX17 | protein DETOXIFICATION 17-like | Down |
| BraA03g060670.3C | CYP81D11 | cytochrome P450 81D11-like | Down |
| BraA03g060710.3C | SRFR1 | uncharacterized LOC103862682 | Down |
| BraA04g000460.3C | GRXC11 | glutaredoxin-C11 | Down |
| BraA04g001200.3C | PIP1.1 | aquaporin PIP1-1 | Down |
| BraA01g015910.3C | HSP23.6 | 23.6 kDa heat shock protein, mitochondrial | Down |
| BraA04g002990.3C |  | uncharacterized LOC103863016 | Down |
| BraA04g003520.3C | ARR9 | two-component response regulator ARR9-like | Down |
| BraA04g003800.3C | SIB1 | sigma factor binding protein 1, chloroplastic-like | Down |
| BraA04g004260.3C | At3g56080 | probable methyltransferase PMT22 | Down |
| BraA04g004420.3C |  | uncharacterized LOC103863144 | Down |
| BraA04g007040.3C |  | uncharacterized LOC103863470 | Down |
| BraA04g007250.3C |  | uncharacterized LOC103863493 | Down |
| BraA03g045760.3C | HSFA7A | heat stress transcription factor A-7a | Down |
| BraA04g007830.3C | CID2 | polyadenylate-binding protein-interacting protein 2 | Down |
| BraA04g008090.3C | RALFL32 | protein RALF-like 32 | Down |
| BraA04g008220.3C | ATJ20 | chaperone protein dnaJ 20, chloroplastic | Down |
| BraA04g010120.3C | At5g03795 | probable glycosyltransferase At3g07620 | Down |
| BraA04g010550.3C | HSP15.7 | 15.7 kDa heat shock protein, peroxisomal | Down |
| BraA04g011940.3C |  | uncharacterized LOC103863895 | Down |
| BraA04g013400.3C |  | uncharacterized LOC103864027 | Down |
| BraA04g018960.3C | At2g25737 | uncharacterized LOC103864686 | Down |
| BraA04g018980.3C | TIP4-1 | aquaporin TIP4-1 | Down |
| BraA04g019040.3C | RLP32 | receptor-like protein 12 | Down |
| BraA04g019100.3C | DEGP3 | putative protease Do-like 3, mitochondrial | Down |
| BraA04g019180.3C | IQM4 | IQ domain-containing protein IQM4 | Down |
| BraA04g020490.3C | KCS12 | 3-ketoacyl-CoA synthase 12 | Down |
| BraA04g022180.3C | NIT2 | nitrilase 2-like | Down |
| BraA04g023100.3C | IP5P7 | type IV inositol polyphosphate 5-phosphatase 7 | Down |
| BraA04g023430.3C | RLP22 | receptor-like protein 12 | Down |
| BraA04g024570.3C |  | uncharacterized LOC103865329 | Down |
| BraA04g024660.3C | RLP24 | receptor-like protein 12 | Down |
| BraA04g026080.3C | UGT73C2 | UDP-glycosyltransferase 73C2-like | Down |
| BraA04g026140.3C | TIP1-1 | aquaporin TIP1-1 | Down |
| BraA04g026210.3C | HIPP05 | heavy metal-associated isoprenylated plant protein 3-like | Down |
| BraA04g026970.3C | DET2 | steroid 5-alpha-reductase DET2 | Down |
| BraA04g027630.3C | JAL22 | jacalin-related lectin 22-like | Down |
| BraA04g028010.3C |  | uncharacterized LOC103865749 | Down |
| BraA04g028470.3C | LAC5 | laccase-5-like | Down |
| BraA04g028760.3C | EXPA8 | expansin-A8-like | Down |
| BraA04g028930.3C | SCAB2 | stomatal closure-related actin-binding protein 2-like | Down |
| BraA05g001920.3C | At2g40900 | WAT1-related protein At2g40900-like | Down |
| BraA04g029150.3C | SPAC7D4.05 | haloacid dehalogenase-like hydrolase domain-containing protein 3 | Down |
| BraA09g047740.3C |  | uncharacterized LOC103865875 | Down |
| BraA04g029900.3C | cao1 | copper methylamine oxidase | Down |
| BraA05g006640.3C | JAL22 | jacalin-related lectin 22-like | Down |
| BraA04g030510.3C | BHLH129 | transcription factor bHLH129-like | Down |
| BraA04g031370.3C | HOL3 | probable thiol methyltransferase 2 | Down |
| BraA03g023210.3C | ARL | ARGOS-like protein | Down |
| BraA04g030720.3C | TRX2 | thioredoxin H2-like | Down |
| BraA04g032260.3C | ROPGAP3 | rho GTPase-activating protein 3-like | Down |
| BraA05g000570.3C | UPB1 | transcription factor UPBEAT1 | Down |
| BraA05g000620.3C | DUF2 | DUF724 domain-containing protein 6-like | Down |
| BraA05g000680.3C | GOLS1 | galactinol synthase 1 | Down |
| BraA05g000900.3C | CYP709B2 | cytochrome P450 709B2 | Down |
| BraA05g001140.3C | ROPGAP3 | rho GTPase-activating protein 3 | Down |
| BraA05g001170.3C | CYP78A6 | cytochrome P450 78A6-like | Down |
| BraA05g001540.3C | BAG6 | BAG family molecular chaperone regulator 6 | Down |
| BraA05g001570.3C |  | uncharacterized LOC103866491 | Down |
| BraA03g021530.3C | EXPA8 | expansin-A8 | Down |
| BraA05g002650.3C |  | uncharacterized LOC103866608 | Down |
| BraA01g017540.3C | At4g26790 | GDSL esterase/lipase At4g26790 | Down |
| BraA05g003510.3C | INT1 | inositol transporter 1 | Down |
| BraA05g003870.3C | UGT74F1 | UDP-glycosyltransferase 74F1 | Down |
| BraA05g004160.3C |  | uncharacterized LOC103866778 | Down |
| BraA03g023450.3C | CYCU4-1 | cyclin-U4-1 | Down |
| BraA05g005590.3C | PIP1-2 | aquaporin PIP1-2 | Down |
| BraA01g018010.3C | GH3.5 | indole-3-acetic acid-amido synthetase GH3.5 | Down |
| BraA05g006280.3C | CYT1 | mannose-1-phosphate guanylyltransferase 1-like | Down |
| BraA05g006330.3C | RCA | ribulose bisphosphate carboxylase/oxygenase activase, chloroplastic-like | Down |
| BraA05g007870.3C | FHY1 | protein FAR-RED ELONGATED HYPOCOTYL 1-like | Down |
| BraA01g018220.3C | CBSX5 | CBS domain-containing protein CBSX5 | Down |
| BraA05g008190.3C | ABCG33 | ABC transporter G family member 33-like | Down |
| BraA05g008270.3C | PIP2-2 | aquaporin PIP2-2 | Down |
| BraA05g008410.3C | UGT86A1 | UDP-glycosyltransferase 86A1-like | Down |
| BraA05g008530.3C | UGT73C5 | UDP-glycosyltransferase 73C5-like | Down |
| BraA05g009320.3C | OFP15 | transcription repressor OFP15-like | Down |
| BraA05g009610.3C | ROPGEF14 | rop guanine nucleotide exchange factor 14-like | Down |
| BraA05g010470.3C | MTERF1 | transcription termination factor MTEF1, chloroplastic | Down |
| BraA05g010610.3C |  | uncharacterized LOC103867448 | Down |
| BraA01g018560.3C | HSP21 | 25.3 kDa heat shock protein, chloroplastic | Down |
| BraA05g011360.3C | NAC041 | NAC domain-containing protein 41-like | Down |
| BraA05g011830.3C | DSP2 | probable tyrosine-protein phosphatase At1g05000 | Down |
| BraA05g012280.3C | HSP70-8 | heat shock 70 kDa protein 8 | Down |
| BraA10g026070.3C |  | tubulin beta chain-like | Down |
| BraA05g013610.3C |  | uncharacterized LOC103867920 | Down |
| BraA01g002280.3C | SAMT | salicylate carboxymethyltransferase | Down |
| BraA01g019000.3C |  | uncharacterized LOC103867927 | Down |
| BraA05g013730.3C | PSBP1 | oxygen-evolving enhancer protein 2-1, chloroplastic-like | Down |
| BraA01g019020.3C | RMA2 | E3 ubiquitin-protein ligase RMA2-like | Down |
| BraA05g014670.3C | HSP17.6B | 17.6 kDa class I heat shock protein 2 | Down |
| BraA08g032240.3C | BHLH74 | transcription factor bHLH74-like | Down |
| BraA08g032300.3C |  | uncharacterized LOC103868126 | Down |
| BraA02g027900.3C | HOP3 | hsp70-Hsp90 organizing protein 3 | Down |
| BraA02g027270.3C | At1g56220 | dormancy-associated protein homolog 3-like | Down |
| BraA05g015000.3C | RVE2 | protein REVEILLE 2 | Down |
| BraA05g016150.3C | SIRP1 | E3 ubiquitin-protein ligase RING1-like | Down |
| BraA08g002150.3C |  | uncharacterized LOC103868510 | Down |
| BraA05g018250.3C |  | uncharacterized LOC103868562 | Down |
| BraA05g019750.3C |  | uncharacterized LOC103868647 | Down |
| BraA05g019900.3C | COL8 | zinc finger protein CONSTANS-LIKE 8 | Down |
| BraA05g024080.3C | IAA7 | auxin-responsive protein IAA7 | Down |
| BraA05g023600.3C | XTH30 | probable xyloglucan endotransglucosylase/hydrolase protein 30 | Down |
| BraA09g040640.3C | ARP1 | RNA-binding protein 24-like | Down |
| BraA09g040860.3C |  | uncharacterized LOC103869077 | Down |
| BraA01g019700.3C | HAT4 | homeobox-leucine zipper protein HAT4 | Down |
| BraA01g019890.3C | VAB | VAN3-binding protein | Down |
| BraA05g026890.3C | At3g58370 | uncharacterized LOC103869330 | Down |
| BraA04g017030.3C | PEN6 | seco-amyrin synthase-like | Down |
| BraA05g028270.3C |  | uncharacterized LOC103869497 | Down |
| BraA01g020190.3C | INT4 | inositol transporter 4-like | Down |
| BraA05g028420.3C | LPAT5 | probable 1-acyl-sn-glycerol-3-phosphate acyltransferase 5 | Down |
| BraA05g028640.3C | PUB29 | U-box domain-containing protein 29 | Down |
| BraA01g020250.3C |  | uncharacterized LOC103869594 | Down |
| BraA01g020370.3C |  | uncharacterized LOC103869692 | Down |
| BraA05g030370.3C | At3g17010 | B3 domain-containing protein At3g17010-like | Down |
| BraA05g030400.3C | AAE7 | acetate/butyrate--CoA ligase AAE7, peroxisomal | Down |
| BraA05g030530.3C | CYCA1-2 | cyclin-A1-2-like | Down |
| BraA05g030960.3C | TIP2-1 | aquaporin TIP2-1 | Down |
| BraA05g031060.3C |  | uncharacterized LOC103869856 | Down |
| BraA05g031600.3C | NAC056 | NAC transcription factor 56 | Down |
| BraA01g037070.3C | SWEET2 | bidirectional sugar transporter SWEET2 | Down |
| BraA05g032880.3C | P58A | dnaJ homolog subfamily B member 2-like | Down |
| BraA05g033000.3C | CCX3 | cation/calcium exchanger 3 | Down |
| BraA05g033270.3C | BGAL1 | beta-galactosidase 1 | Down |
| BraA05g033340.3C | DIR7 | dirigent protein 7-like | Down |
| BraA05g033540.3C |  | serine/arginine-rich SC35-like splicing factor SCL30A | Down |
| BraA05g033670.3C | CPN60B2 | chaperonin 60 subunit beta 2, chloroplastic | Down |
| BraA05g034710.3C |  | protein ABHD18 | Down |
| BraA05g034950.3C | SCL30A | serine/arginine-rich SC35-like splicing factor SCL30A | Down |
| BraA07g037470.3C | SCPL7 | serine carboxypeptidase-like 7 | Down |
| BraA05g036630.3C |  | uncharacterized LOC103870496 | Down |
| BraA05g036930.3C | RVE8 | protein REVEILLE 8 | Down |
| BraA05g036950.3C | PDS1 | phytoene dehydrogenase, chloroplastic/chromoplastic | Down |
| BraA05g037090.3C | fes1 | uncharacterized LOC103870549 | Down |
| BraA05g037290.3C | At4g19900 | lactosylceramide 4-alpha-galactosyltransferase-like | Down |
| BraA05g037360.3C | LHCB4.2 | chlorophyll a-b binding protein CP29.2, chloroplastic | Down |
| BraA05g038320.3C |  | uncharacterized LOC103870677 | Down |
| BraA05g038330.3C | BHLH62 | transcription factor bHLH62 | Down |
| BraA05g038610.3C | At3g07010 | probable pectate lyase 8 | Down |
| BraA05g040010.3C | At3g05165 | sugar transporter ERD6-like 11 | Down |
| BraA01g021300.3C | At4g15430 | CSC1-like protein At4g15430 | Down |
| BraA05g041050.3C | TCP13 | transcription factor TCP13 | Down |
| BraA05g040740.3C | HIR3 | hypersensitive-induced response protein 3 | Down |
| BraA05g040580.3C | STY13 | serine/threonine-protein kinase HT1 | Down |
| BraA06g000210.3C |  | uncharacterized LOC103871005 | Down |
| BraA06g000630.3C | SCL30A | serine/arginine-rich SC35-like splicing factor SCL33 | Down |
| BraA06g001500.3C | ERF8 | ethylene-responsive transcription factor 4-like | Down |
| BraA06g001970.3C | HSP26.5 | 26.5 kDa heat shock protein, mitochondrial | Down |
| BraA01g000250.3C | BBX20 | B-box zinc finger protein 20-like | Down |
| BraA06g002680.3C |  | photosystem II 5 kDa protein, chloroplastic | Down |
| BraA05g015780.3C | LIGB | extradiol ring-cleavage dioxygenase-like | Down |
| BraA06g003970.3C | BNM2A | BURP domain-containing protein BNM2A-like | Down |
| BraA06g003980.3C |  | uncharacterized LOC103871415 | Down |
| BraA06g004040.3C | ATL76 | E3 ubiquitin-protein ligase ATL76-like | Down |
| BraA06g004700.3C | CIPK17 | CBL-interacting serine/threonine-protein kinase 17-like | Down |
| BraA06g005720.3C | At1g08570 | thioredoxin-like 1-1, chloroplastic | Down |
| BraA06g006060.3C | SR30 | serine/arginine-rich splicing factor SR30 | Down |
| BraA06g007010.3C | PP2A9 | protein PHLOEM PROTEIN 2-LIKE A9 | Down |
| BraA01g021980.3C | TIFY4B | protein TIFY 4B | Down |
| BraA06g007440.3C | GH3.12 | 4-substituted benzoates-glutamate ligase GH3.12-like | Down |
| BraA06g007500.3C | AMSH2 | AMSH-like ubiquitin thioesterase 2 | Down |
| BraA06g008030.3C | CNR5 | uncharacterized LOC103871896 | Down |
| BraA06g009580.3C | At1g12010 | 1-aminocyclopropane-1-carboxylate oxidase 3 | Down |
| BraA06g010320.3C | MPSR1 | E3 ubiquitin-protein ligase RING1-like | Down |
| BraA06g010390.3C | PKS2 | protein PHYTOCHROME KINASE SUBSTRATE 2 | Down |
| BraA06g010680.3C | AHL17 | AT-hook motif nuclear-localized protein 28 | Down |
| BraA06g011800.3C |  | uncharacterized LOC103872365 | Down |
| BraA06g012060.3C | HSP70-5 | heat shock 70 kDa protein 5 | Down |
| BraA06g012940.3C | VQ1 | VQ motif-containing protein 1 | Down |
| BraA06g013450.3C | PEPR2 | leucine-rich repeat receptor-like protein kinase PEPR2 | Down |
| BraA06g013660.3C | EGY3 | probable zinc metallopeptidase EGY3, chloroplastic | Down |
| BraA03g065120.3C | At1g61480 | G-type lectin S-receptor-like serine/threonine-protein kinase At1g61480 | Down |
| BraA06g014110.3C | PCMP-H8 | pentatricopeptide repeat-containing protein At1g18485 | Down |
| BraA06g014310.3C | PDCB3 | PLASMODESMATA CALLOSE-BINDING PROTEIN 3-like | Down |
| BraA01g022780.3C | PSBO2 | oxygen-evolving enhancer protein 1-2, chloroplastic | Down |
| BraA06g015440.3C | SAUR50 | auxin-responsive protein SAUR22 | Down |
| BraA06g016050.3C | AAE1 | probable acyl-activating enzyme 1, peroxisomal | Down |
| BraA06g016090.3C | CAT3 | catalase-3 | Down |
| BraA08g027730.3C | PBL21 | serine/threonine-protein kinase CDL1-like | Down |
| BraA07g015700.3C | DPMS1 | probable dolichol-phosphate mannosyltransferase | Down |
| BraA06g017470.3C | RAX3 | transcription factor RAX3 | Down |
| BraA06g017870.3C | WSD1 | O-acyltransferase WSD1-like | Down |
| BraA06g018360.3C | NFYC1 | nuclear transcription factor Y subunit C-1 | Down |
| BraA06g019540.3C | LHCA4 | chlorophyll a-b binding protein 4, chloroplastic | Down |
| BraA06g019580.3C | TIP5-1 | probable aquaporin TIP5-1 | Down |
| BraA06g020450.3C | HSP17.6C | 17.4 kDa class I heat shock protein | Down |
| BraA06g020530.3C | MYB48 | transcription factor MYB48 | Down |
| BraA06g020950.3C | LECRK14 | L-type lectin-domain containing receptor kinase I.3 | Down |
| BraA06g024440.3C | CYP71A26 | cytochrome P450 71A26 | Down |
| BraA06g024430.3C | WNK3 | probable serine/threonine-protein kinase WNK3 | Down |
| BraA06g024180.3C | ABCA8 | ABC transporter A family member 8 | Down |
| BraA06g026770.3C | BT1 | BTB/POZ and TAZ domain-containing protein 1 | Down |
| BraA06g026800.3C | EIF(ISO)4G1 | uncharacterized LOC103873741 | Down |
| BraA06g027970.3C |  | uncharacterized LOC103873874 | Down |
| BraA06g027990.3C | NAC104 | NAC domain-containing protein 104-like | Down |
| BraA06g028320.3C | PEP1 | elicitor peptide 1 | Down |
| BraA06g028930.3C |  | uncharacterized LOC103873983 | Down |
| BraA06g029730.3C | VIL2 | VIN3-like protein 2 | Down |
| BraA06g029890.3C | HBI1 | transcription factor HBI1-like | Down |
| BraA06g030460.3C | ATL44 | probable E3 ubiquitin-protein ligase ATL44 | Down |
| BraA06g030660.3C | HIPP27 | heavy metal-associated isoprenylated plant protein 27 | Down |
| BraA06g030710.3C | TCP7 | transcription factor TCP7-like | Down |
| BraA06g031150.3C | REM1.4 | remorin-like | Down |
| BraA06g031610.3C | GMI1 | uncharacterized LOC103874295 | Down |
| BraA06g032560.3C |  | uncharacterized LOC103874386 | Down |
| BraA06g032600.3C |  | uncharacterized LOC103874389 | Down |
| BraA06g033010.3C | KO | ent-kaurene oxidase, chloroplastic | Down |
| BraA06g033230.3C | MAKR1 | probable membrane-associated kinase regulator 1 | Down |
| BraA06g034510.3C | BZIP63 | basic leucine zipper 63 | Down |
| BraA06g034760.3C | BXL1 | beta-D-xylosidase 1 | Down |
| BraA06g025490.3C | HSFB2A | heat stress transcription factor B-2a | Down |
| BraA02g043490.3C | AGL42 | MADS-box protein AGL42-like | Down |
| BraA05g023200.3C | NPF7.3 | protein NRT1/ PTR FAMILY 7.3 | Down |
| BraA05g023030.3C | MYC2 | transcription factor MYC2 | Down |
| BraA06g026380.3C | NPF4.7 | protein NRT1/ PTR FAMILY 4.7 | Down |
| BraA06g035490.3C | FKBP65 | peptidyl-prolyl cis-trans isomerase FKBP65 | Down |
| BraA06g035270.3C | SDI1 | protein SULFUR DEFICIENCY-INDUCED 1 | Down |
| BraA08g032140.3C | RPP8 | disease resistance protein RPP8-like | Down |
| BraA06g036270.3C | COBL2 | COBRA-like protein 2 | Down |
| BraA06g036490.3C |  | UPF0301 protein TC_0483 | Down |
| BraA02g038020.3C | At5g40210 | WAT1-related protein At5g40210 | Down |
| BraA06g037470.3C | At5g66910 | probable disease resistance protein At5g66900 | Down |
| BraA06g038050.3C |  | cysteine-rich and transmembrane domain-containing protein A-like | Down |
| BraA06g038250.3C | TIP1-2 | aquaporin TIP1-2 | Down |
| BraA06g037170.3C |  | uncharacterized LOC103875294 | Down |
| BraA02g036820.3C | CYP71B26 | cytochrome P450 71B26-like | Down |
| BraA06g038960.3C | At2g03980 | GDSL esterase/lipase At2g03980-like | Down |
| BraA06g038970.3C | PI4KG7 | phosphatidylinositol 4-kinase gamma 7 | Down |
| BraA06g039400.3C | FUT2 | fucosyltransferase 2 | Down |
| BraA06g039540.3C | PKS1 | protein PHYTOCHROME KINASE SUBSTRATE 1 | Down |
| BraA04g022920.3C | RLP24 | receptor-like protein 12 | Down |
| BraA06g037470.3C | At5g66900 | probable disease resistance protein At5g66900 | Down |
| BraA07g022780.3C |  | uncharacterized LOC108868968 | Down |
| BraA02g040460.3C | AHL14 | AT-hook motif nuclear-localized protein 14-like | Down |
| BraA07g030360.3C | NAC029 | NAC transcription factor 29 | Down |
| BraA06g034460.3C | WRKY74 | probable WRKY transcription factor 74 | Down |
| BraA09g001170.3C | XTH9 | xyloglucan endotransglucosylase/hydrolase protein 9 | Down |

**Table S4** Top30 up GO enrichment analysis in CT(LTvsNT).

| **id** | **Term** | **Category** | **ListHits** |
| --- | --- | --- | --- |
| GO:0009651 | response to salt stress | biological_process | 94 |
| GO:0009409 | response to cold | biological_process | 86 |
| GO:0006952 | defense response | biological_process | 71 |
| GO:0006355 | regulation of transcription, DNA-templated | biological_process | 68 |
| GO:0009737 | response to abscisic acid | biological_process | 66 |
| GO:0009414 | response to water deprivation | biological_process | 62 |
| GO:0009611 | response to wounding | biological_process | 60 |
| GO:0050832 | defense response to fungus | biological_process | 47 |
| GO:0046686 | response to cadmium ion | biological_process | 47 |
| GO:0009753 | response to jasmonic acid | biological_process | 40 |
| GO:0005634 | nucleus | cellular_component | 396 |
| GO:0016021 | integral component of membrane | cellular_component | 301 |
| GO:0005886 | plasma membrane | cellular_component | 226 |
| GO:0005737 | cytoplasm | cellular_component | 186 |
| GO:0009507 | chloroplast | cellular_component | 163 |
| GO:0005829 | cytosol | cellular_component | 163 |
| GO:0016020 | membrane | cellular_component | 95 |
| GO:0009506 | plasmodesma | cellular_component | 94 |
| GO:0005739 | mitochondrion | cellular_component | 91 |
| GO:0005783 | endoplasmic reticulum | cellular_component | 85 |
| GO:0046872 | metal ion binding | molecular_function | 183 |
| GO:0003700 | DNA-binding transcription factor activity | molecular_function | 169 |
| GO:0005524 | ATP binding | molecular_function | 157 |
| GO:0003677 | DNA binding | molecular_function | 136 |
| GO:0043565 | sequence-specific DNA binding | molecular_function | 90 |
| GO:0020037 | heme binding | molecular_function | 45 |
| GO:0044212 | transcription regulatory region DNA binding | molecular_function | 44 |
| GO:0008270 | zinc ion binding | molecular_function | 40 |
| GO:0046983 | protein dimerization activity | molecular_function | 34 |
| GO:0005506 | iron ion binding | molecular_function | 33 |

**Table S5** Top30 up GO enrichment analysis in CS(LTvsNT).

| **id** | **Term** | **Category** | **ListHits** |
| --- | --- | --- | --- |
| GO:0009651 | response to salt stress | biological_process | 89 |
| GO:0006355 | regulation of transcription, DNA-templated | biological_process | 85 |
| GO:0006952 | defense response | biological_process | 78 |
| GO:0009409 | response to cold | biological_process | 75 |
| GO:0009737 | response to abscisic acid | biological_process | 71 |
| GO:0051301 | cell division | biological_process | 62 |
| GO:0009414 | response to water deprivation | biological_process | 61 |
| GO:0005975 | carbohydrate metabolic process | biological_process | 51 |
| GO:0050832 | defense response to fungus | biological_process | 44 |
| GO:0009738 | abscisic acid-activated signaling pathway | biological_process | 43 |
| GO:0005634 | nucleus | cellular_component | 538 |
| GO:0016021 | integral component of membrane | cellular_component | 315 |
| GO:0005886 | plasma membrane | cellular_component | 277 |
| GO:0005737 | cytoplasm | cellular_component | 257 |
| GO:0005829 | cytosol | cellular_component | 183 |
| GO:0009507 | chloroplast | cellular_component | 149 |
| GO:0009506 | plasmodesma | cellular_component | 121 |
| GO:0005576 | extracellular region | cellular_component | 94 |
| GO:0005794 | Golgi apparatus | cellular_component | 93 |
| GO:0016020 | membrane | cellular_component | 93 |
| GO:0046872 | metal ion binding | molecular_function | 232 |
| GO:0005524 | ATP binding | molecular_function | 203 |
| GO:0003677 | DNA binding | molecular_function | 200 |
| GO:0003700 | DNA-binding transcription factor activity | molecular_function | 197 |
| GO:0043565 | sequence-specific DNA binding | molecular_function | 100 |
| GO:0008270 | zinc ion binding | molecular_function | 50 |
| GO:0004674 | protein serine/threonine kinase activity | molecular_function | 49 |
| GO:0044212 | transcription regulatory region DNA binding | molecular_function | 47 |
| GO:0020037 | heme binding | molecular_function | 39 |
| GO:0046982 | protein heterodimerization activity | molecular_function | 38 |

**Table S6** Primer pairs used to detect the expression of selected genes.

| **gene symbol** | **gene_id** | **Primer pairs** | |
| --- | --- | --- | --- |
|  |  | **Forward** | **Reverse** |
| APX1 | BraA08g033370.3C | GGAAATGCCACAAGGATAG | GTCCAATAGCGCCTTGTC |
| At4g31140 | BraA01g006680.3C | ATCTAGCGCAGAATGTTTCGT | ACCACCCGAAGAATCAGAC |
| BAM2 | BraA09g000540.3C | TTACGACAGAGAAGGCTACAAC | GATCCGAAACAGCTTCACCA |
| LEA14 | BraA08g035760.3C | AGTCACCAATCCTTACGGTCA | ACCAACGTCTCGAGCCAA |
| TIFY11B | BraA07g036860.3C | TCGTCGATCTAAGTGAACC | GATTTGCTTCTTTGGCTGCTT |
| TIFY6B | BraA01g034810.3C | CTCATCAACAAGTGGTCCATC | CTCCGAACCCATTTGAGCTAC |
| TIFY10A | BraA09g056830.3C | CGCTCCTATCCCAAACCAAC | TGCCTTTGAAGTAACCCTGTCC |
| TIFY7 | BraA07g029850.3C | GCAGAAGCCAGTATAGCGGAG | TTTCAAACCATTCCCGGCACA |
| TIFY9 | BraA03g005650.3C | GATCCCTATTTCTCCGGTCCA | CTTCAGAATATCATCAGCCTGGT |
| TIFY7 | BraA02g020000.3C | CATTCTTCTTTCCGTCCT | AAGACGCTAACAGTTCCACC |
| TIFY9 | BraA10g025540.3C | AAGGTGCGATTTCTAAGATGG | AGGATCCGTTGATGATCTTCCTT |
| SPS1 | BraA10g020290.3C | GATGCTCGCCCTTCGCTT | CTCGTAGCAACAGCCTTGGTC |
| AOC2 | BraA06g038770.3C | AACTAGACCGACACAGCCCAA | CTTTCTTCTCCGGGACGTGTG |
| bHLH27 | BraA03g055360.3C | AGAGAAGACTAGAAGCAGAGA | CTCTCCCATCCACGTCACC |
| WRKY29 | BraA03g051140.3C | ATCCGCTGATGAATCTTCCGTTG | ATGGTAAACTGGTACTCATGCCT |
| BGLU10 | BraA04g000610.3C | CGACCGCAAAATCATAGAAGAC | CTGTAAGCACCAATGGCGAA |
| BGLU15 | BraA05g004340.3C | TCCGTGGCGCAGAAATTGT | ATCACACCTGCAACGTACCC |
| BGLU25 | BraA01g043570.3C | AGGACAAGGGAGTGAGTGAAA | ACTCAAGAAACCGCCATATTCA |
| BGLU27 | BraA09g049950.3C | ACAAAGAAGCATGTGAACGAG | AGATTGAGCTGCGGTAAATGA |
| Actin | BraA02g003190.3C | TGGGTTTGCTGGTGACGAT | TGCCTAGGACGACCAACAATACT |
